# Supplementary material for: Wdr4 regulates ribosome biogenesis and intestinal homeostasis via let-7
Source: EMBO Rep. 2026 Feb 9;27(8):1870–903. doi: 10.1038/s44319-026-00701-y (PMC13121520; doi:10.1038/s44319-026-00701-y)
Supplement: Supplementary file 1 — Appendix [file 44319_2026_701_MOESM1_ESM.pdf]

Appendix for Wdr4 Regulates Ribosome Biogenesis and Intestinal Homeostasis via *let-7*

Table of Contents

Appendix Figure S1 Lower WDR4 expression in stomach and rectal cancer is associated with poor patient prognosis. pg.2

Appendix Figure S2 dWdr4 mutant males do not show leaky gut phenotype pg. 3

Appendix Figure S3 Wdr4 loss decreases male gut length but does not affect the thickness of the anterior and posterior midguts. pg. 4

Appendix Table S1-S3 Genes involved in immune response, lipid metabolism, gut digestion, ROS removal and production, and ribosome protein altered upon Wdr4 loss pg. 5

Appendix Table S4 Genotypes used in each figure and figure EV pg. 8

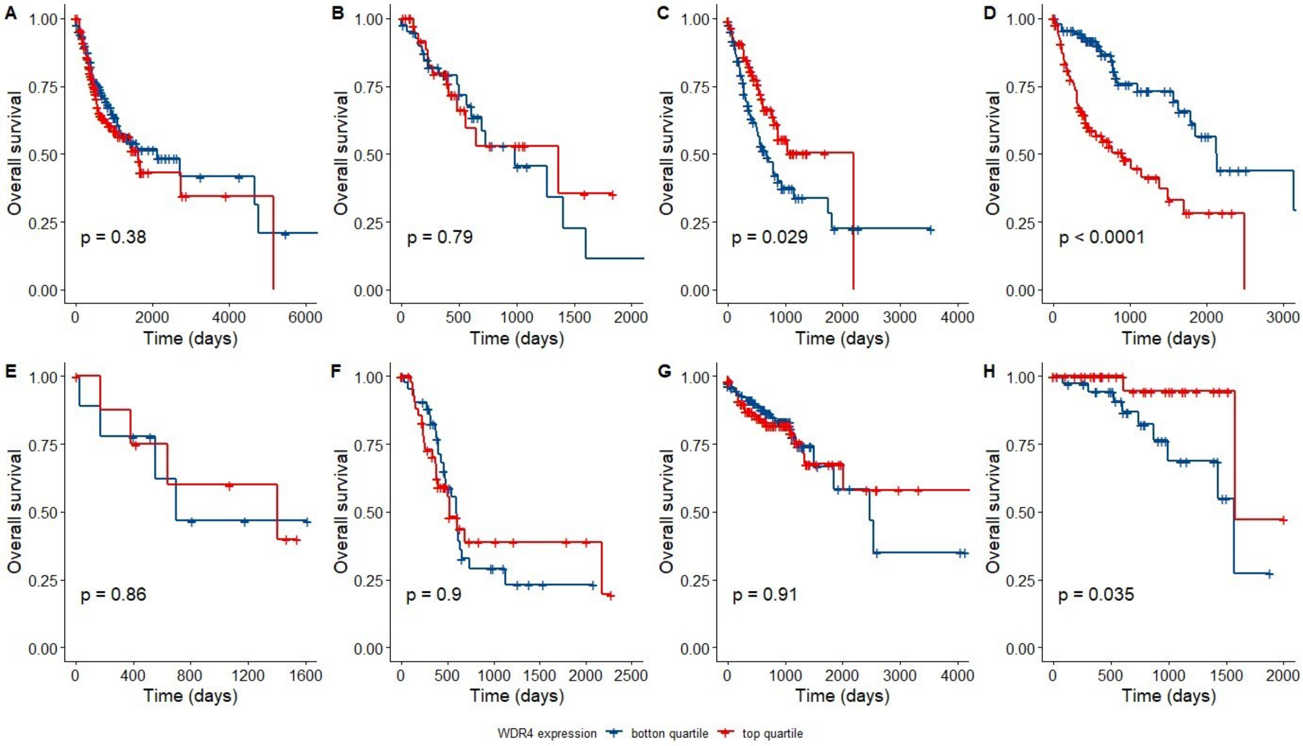

Appendix Figure S1 Lower WDR4 expression in stomach and rectal cancer is associated with poor patient prognosis. RNA sequencing data from the primary tumors of gastrointestinal (GI) cancers, including head and neck squamous cell carcinoma (HNSC), esophageal carcinoma (ESCA), stomach adenocarcinoma (STAD), liver hepatocellular carcinoma (LIHC), cholangiocarcinoma (CHOL), pancreatic adenocarcinoma (PAAD), colon adenocarcinoma (COAD), and rectum adenocarcinoma (READ), were obtained from The Cancer Genome Atlas (TCGA) database using the TCGAbiolinks R package (Colaprico et al., 2016). To test the prognostic value of WDR4 expression in GI cancers, the Kaplan-Meier method and log-rank test were used to compare the overall survival of patients in the highest quartile of WDR4 expression and those in the lowest quartile for each cancer type. All statistical analyses were performed in R version 4.0.5. Kaplan-Meier curves represent the overall survival of patient groups selected according to the highest and lowest quartile of WDR4 expression (red: top, blue: bottom) in tumor samples from the TCGA dataset. A, Head and Neck squamous cell carcinoma; B, Esophageal carcinoma; C, Stomach adenocarcinoma; D, Liver hepatocellular carcinoma; E, Cholangiocarcinoma; F, Pancreatic adenocarcinoma; G, Colon adenocarcinoma; H, Rectum adenocarcinoma. Statistically significant differences in were analyzed by Chi-square, in C′ and E′ by non-parametric Mann–Whitney U test. The prognostic value of WDR4 expression in GI cancers, the Kaplan-Meier method and log-rank test were used to compare the overall survival of patients in the highest quartile of WDR4 expression and those in the lowest quartile for each cancer type. A *P*-value < 0.05 was considered statistically significant.

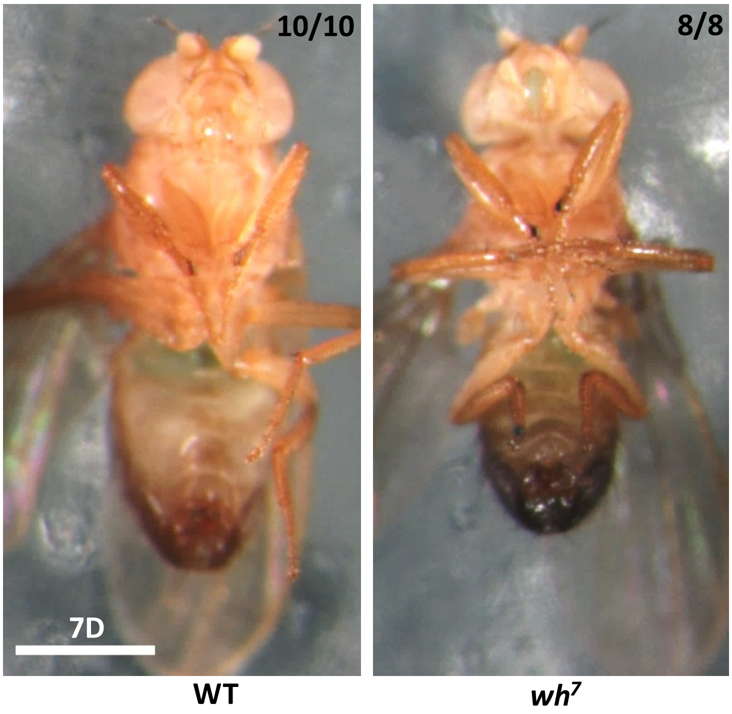

Appendix Figure S2 dWdr4 mutant males do not show leaky gut phenotype. Smurf assay of wild-type (WT) and dwdr4 mutant (*wh*^{7}) male flies. The fraction of flies exhibiting the depicted pattern is provided in each panel.

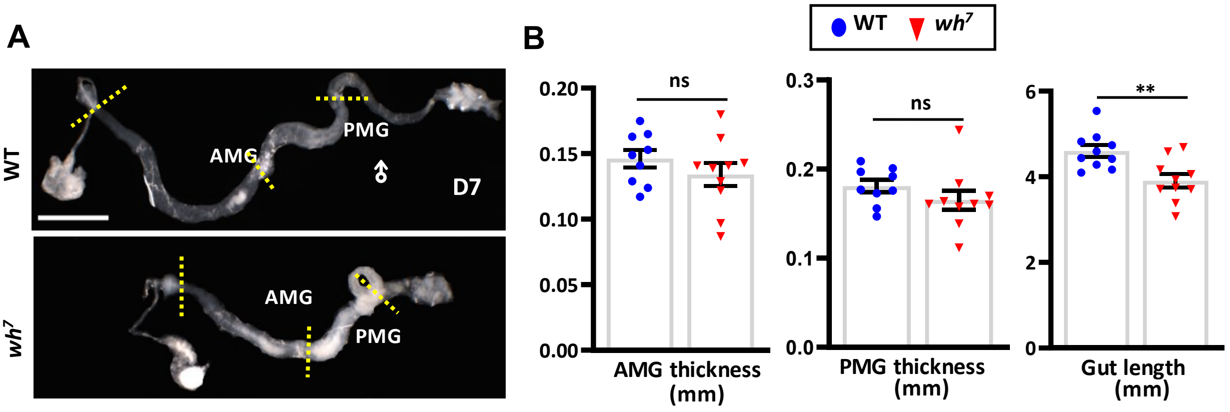

Appendix Figure S3 Wdr4 loss decreases male gut length but does not affect the thickness of the anterior and posterior midguts. (A) Gut morphology of wild-type (WT), and wh7 mutants. Dashed lines roughly define the region of AMG and PMG. (B) Gut length and thickness of anterior midgut (AMG) and posterior midgut (PMG) in 7-day-old wild-type and *dwdr4* mutant (*wh*^{7}) male flies. Statistically significant differences were identified by non-parametric Mann–Whitney U test. **, *P*<0.01. ns., not significant. Error bars, SD.

Appendix Table S1 RNA-seq analysis of dwdr4 mutant guts reveals altered genes in immune response, lipid metabolism, and digestion

Function	Gene	Symbol	*wh*^{7} vs WT log2FC	P value	padj (FDR)
			**Diverse Immune Effectors**		
**Antiviral Response**	FBgn0043841	vir-1	-0.7305	0.00124	0.031399362
	FBgn0004428	LysE	-1.4762	1.1E-07	1.01548E-05
	FBgn0004425	LysB	-0.1957	0.0159	0.232868427
**Humoral Immune Defense**	FBgn0004429	LysP	-0.348	0.02366	0.307180604
	FBgn0004430	LysS	-8.2046	4E-109	2.365E-105
	FBgn0035806	PGRP-SD	-1.7005	0.00015	0.005721369
	FBgn0037906	PGRP-LB	-1.1799	3.9E-05	0.001856967
			**Lipid Homeostasis**		
**Neuropeptide**	FBgn0037976	Tk	-0.2517	0.01164	0.183605402
**Tk receptor**	FBgn0004622	TkR99D	-0.2475	0.02247	0.296471034
**Insulin**	FBgn0044050	Ilp3	-0.8321	0.00365	0.075191067
			**Digestion**		
	FBgn0035886	Jon66Ci	2.62308	6.6E-11	1.1705E-08
**Protease**	FBgn0031653	Jon25Biii	0.50828	0.00472	0.091207142
	FBgn0031654	Jon25Bii	0.42901	0.00471	0.091073829
	FBgn0003863	alphaTry	-0.0415	0.09986	0.733192126
	FBgn0010358	deltaTry	-0.3527	0.00645	0.114860834
**Peptidase**	FBgn0010359	gammaTry	-0.0929	0.04131	0.444121457
	FBgn0010425	epsilonTry	-0.071	0.05818	0.552142764
	FBgn0011555	thetaTry	-0.1479	0.02348	0.305896774
**Amino Acid Storage**	FBgn0002563	Lsp1beta	-2.5844	2.4E-07	1.97542E-05
	FBgn0002564	Lsp1gamma	-4.1245	1.9E-27	1.36343E-24
**Sterol Transport**	FBgn0039800	Npc2g	-0.1177	0.05734	0.547175356
	FBgn0051410	Npc2e	-1.3443	7.9E-06	0.000468642
**Carbohydrate Digestion**	FBgn0020506	Amyrel	3.14937	1.4E-23	7.67342E-21
	FBgn0041630	Hexo1	-0.2056	0.01621	0.236154778

Appendix Table S2 RNA-seq analysis of dwdr4 mutant guts reveals altered ribosomal protein genes

Transcript_ID	Gene	Symbol	*wh*^{7} vs WT Log2FC	P_value	padj (FDR)_
FBtr0070155	FBgn0002579	RpL36	0.397772714	0.049243702	0.36649981
FBtr0070800	FBgn0029785	RpL35	0.725106064	7.93028E-06	0.000261089
FBtr0070982	FBgn0029897	RpL17	3.547526805	1.43933E-07	7.06417E-06
FBtr0071592	FBgn0016726	RpL29	0.785767461	4.1589E-05	0.001182943
FBtr0072174	FBgn0034968	RpL12	0.523313987	0.006207748	0.083092908
FBtr0072185	FBgn0023170	RpL39	0.59563357	0.000262649	0.005899141
FBtr0072406	FBgn0285950	RpL19	0.430767847	0.011303345	0.132612907
FBtr0073098	FBgn0035422	RpL28	2.092138458	0.001476291	0.025757828

FBtr0073691	FBgn0030433	mRpL49	7.439733698	8.17137E-09	5.45506E-07
FBtr0075066	FBgn0036825	RpL26	0.447817537	0.022971096	0.222353165
FBtr0077452	FBgn0285948	RpL27A	0.504934745	0.003217902	0.049268659
FBtr0077470	FBgn0003941	RpL40	0.429336728	0.021809573	0.21392269
FBtr0078056	FBgn0002593	RpLP1	0.472457582	0.003104008	0.047773249
FBtr0079016	FBgn0261608	RpL37A	0.510830504	0.010035003	0.12099986
FBtr0079546	FBgn0031980	RpL36A	0.499966323	0.007837075	0.100730359
FBtr0079946	FBgn0005593	RpL7	0.334192899	0.039107457	0.315418421
FBtr0081181	FBgn0086710	RpL30	0.387234934	0.007286692	0.09503898
FBtr0082346	FBgn0020910	RpL3	0.311849759	0.042693118	0.334960244
FBtr0085594	FBgn0002626	RpL32	1.439097032	0.036409725	0.300145237
FBtr0088525	FBgn0285949	RpL31	0.425841701	0.025047217	0.235817546
FBtr0111120	FBgn0040007	RpL38	0.511148259	0.012842601	0.145518365
FBtr0302570	FBgn0261608	RpL37A	0.746137826	0.007867793	0.101040494
FBtr0308333	FBgn0028697	RpL15	4.741835864	0.004311324	0.062541334
FBtr0310482	FBgn0011272	RpL13	2.169932176	0.000171994	0.004077254
FBtr0330403	FBgn0030616	RpL37a	0.415641091	0.007692296	0.099302927
FBtr0333370	FBgn0035753	RpL18	0.766798935	0.000872618	0.016638121
FBtr0334787	FBgn0003941	RpL40	0.858092223	0.000445047	0.00933155
FBtr0335135	FBgn0053002	mRpL27	2.766083181	2.07755E-07	9.52616E-06
FBtr0343328	FBgn0014026	RpL7A	1.364904913	0.090408192	0.516732435
FBtr0345292	FBgn0029897	RpL17	2.843801971	3.4825E-05	0.001005412
FBtr0474640	FBgn0026372	RpL23A	2.205156028	0.000339605	0.007353394
FBtr0071094	FBgn0004403	RpS14a	0.485131862	0.005426077	0.074899643
FBtr0071096	FBgn0004404	RpS14b	0.480479353	0.011954	0.13834797
FBtr0071360	FBgn0030136	RpS28b	0.649761507	0.001662034	0.028272709
FBtr0071855	FBgn0034743	RpS16	0.411040308	0.014790017	0.161175914
FBtr0073792	FBgn0010198	RpS15Aa	1.221876106	0.001097208	0.02022205
FBtr0074732	FBgn0285947	RpS10b	1.706920486	2.13446E-07	9.7534E-06
FBtr0075878	FBgn0286213	RpS12	0.460196393	0.021835347	0.214017086
FBtr0076423	FBgn0010408	RpS9	0.364763632	0.041125136	0.326426916
FBtr0076479	FBgn0005533	RpS17	0.544311384	0.001332433	0.023700324
FBtr0081089	FBgn0261597	RpS26	0.473300162	0.024815081	0.23497757
FBtr0081091	FBgn0261597	RpS26	2.154254848	2.43142E-22	8.41679E-20
FBtr0082136	FBgn0261599	RpS29	0.485070482	0.009859693	0.119532401
FBtr0082370	FBgn0086472	RpS25	0.382581744	0.03088072	0.271363299
FBtr0084410	FBgn0002622	RpS3	0.514390122	0.020274259	0.202918691
FBtr0086273	FBgn0010411	RpS18	0.575863353	0.016294915	0.173578831
FBtr0087123	FBgn0034138	RpS15	0.497819425	0.001364704	0.024193146
FBtr0089422	FBgn0039757	RpS7	0.739196187	0.006003872	0.081101229
FBtr0089425	FBgn0039757	RpS7	0.688837162	0.023094882	0.223143876
FBtr0300828	FBgn0010198	RpS15Aa	0.443056371	0.035230033	0.294171882
FBtr0304693	FBgn0039300	RpS27	0.479207608	0.002609543	0.041167093
FBtr0308296	FBgn0017545	RpS3A	0.920902802	0.001811332	0.030538005
FBtr0339970	FBgn0033912	RpS23	0.527055436	0.013454296	0.150379227
FBtr0346158	FBgn0039713	RpS8	0.456873756	0.014667345	0.160366606
FBtr0346591	FBgn0261597	RpS26	1.99752251	0.001407432	0.024777804

Appendix Table S3 RNA-seq analysis of dwdr4 mutant female guts altered genes in redox activity

Function	Gene	Symbol	*wh*[7] vs WT log2FC	P value	padj (FDR)
**Glutathione Transferase**	FBgn0001149	GstD1	1.668038376	9.39057E-09	1.14323E-06
	FBgn0010038	GstD2	3.53317387	1.04069E-25	6.54593E-23
	FBgn0010040	GstD4	1.399659234	0.001019884	0.027301045
	FBgn0010042	GstD6	1.107945402	0.002629187	0.058139947
	FBgn0010043	GstD7	1.680179562	3.11571E-06	0.000200432
	FBgn0010044	GstD8	0.364677822	0.01639339	0.237956361
	FBgn0063493	GstE7	2.052386042	1.26054E-07	1.11498E-05
**ROS Production**	FBgn0283531	Duox	0.231733687	0.037798364	0.418831599
	FBgn0010019	Cyp4g1	1.785315494	1.73939E-06	0.000119354
	FBgn0031432	Cyp309a1	1.718181131	7.31134E-08	6.89825E-06

Gray marks the FDR value is not smaller than *P<0.05*.

Appendix Table S4. Phenotypes of files used in each figure and Figure EV

**Figure 1**	**A**	yw and wh7/wh7 and wh7/wh7;dwdr4-gfp/+
	**B-C**	yw and wh7/wh7
	**E**	dwdr4-gfp
	**F-F'**	yw and wh7/wh7 and wh7/wh7;dwdr4-gfp/+
**Figure 2**	**A-C'**	w^-;esg-GAL4/UAS-mcD8::gfp and wh7/wh7;esg-GAL4/UAS-mcD8::gfp
	**D-E**	yw and wh7/wh7
	**E'**	yw and wh7/wh7 and wh7/wh7;dwdr4-gfp/+
**Figure 3**	**A-A''**	FRT19A/hs-flpGAL80FRT19A;actGAL4/UAS-mcD8::gfp and wh7FRT19A/hs-flpGAL80FRT19A;actGAL4/UAS-mcD8::gfp
	**B-B'**	Dl-GAL4/UAS-egfpRNAi and dwdr4RNAi$^{(P)}$/+;Dl-GAL4/+
	**C**	w^-;tub-GAL80TS/+;Dl-Gal4 UAS-gfp/UAS-mcherryRNAi and w^-;tub-GAL80TS/dwdr4RNAi$^{(P)}$
	**C'**	w^-;tub-GAL80^{TS}/+;Dl-Gal4 UAS-gfp/UAS-mcherryRNAi and w^-;tub-GAL80TS/dwdr4RNAi$^{(P)}$;Dl-GAL4 UAS-gfp/+ and w^-;tub-GAL80TS/dwdr4RNAi$^{(t)}$;Dl-GAL4 UAS-gfp/+
**Figure 4**	**B-B'**	yw;esg-GAL4/UAS-mcD8::gfp and wh7/wh7;esg-GAL4/UAS-mcD8::gfp
	**C-C'**	yw and wh7/wh7
	**D**	w^-;esg-GAL4 UAS-gfp/dwdr4RNAi$^{(P)}$; Su(H)GBE-lacZ tubGAL80TS/+
	**D'**	w^-;esg-GAL4 UAS-gfp/+; Su(H)GBE-lacZ,tubGAL80TS/UAS-mcherryRNAi and w^-;esg-GAL4 UAS-gfp/dwdr4RNAi$^{(P)}$; Su(H)GBE-lacZ tubGAL80TS/+
**Figure 5**	**A**	w^-;tub-GAL80^{TS}/+;Dl-GAL4 UAS-gfp/UAS-mcherryRNAi and w^-;tub-GAL80TS/dwdr4RNAi$^{(P)}$;Dl-GAL4 UAS-gfp/+
	**B**	w^-;esg-GAL4 UAS-gfp/dwdr4RNAi$^{(P)}$; Su(H)GBE-lacZ tubGAL80TS/+ and w^-;esg-GAL4 UAS-gfp/dwdr4RNAi$^{(P)}$; Su(H)GBE-lacZ tubGAL80TS/UAS-TORRNAi
	**B'**	w^-;esg-GAL4 UAS-gfp/+; Su(H)GBE-lacZ,tubGAL80TS/UAS-mcherryRNAi and w^-;esg-GAL4 UAS-gfp/dwdr4RNAi$^{(P)}$; Su(H)GBE-lacZ tubGAL80TS/+ and w^-;esg-GAL4 UAS-gfp/dwdr4RNAi$^{(P)}$; Su(H)GBE-lacZ tubGAL80TS/UAS-TORRNAi and w^-;esg-GAL4 UAS-gfp/+; Su(H)GBE-lacZ tubGAL80TS/UAS-TORRNAi
	**C**	wh7/wh7
	**C'**	yw and wh7/wh7
	**D**	w^-;tub-GAL80TS/dwdr4RNAi$^{(P)}$;Dl-GAL4 UAS-gfp/+
	**D'**	w^-;tub-GAL80^{TS}/+;Dl-GAL4 UAS-gfp/UAS-mcherryRNAi and w^-;tub-GAL80TS/dwdr4RNAi$^{(P)}$;Dl-GAL4 UAS-gfp/+
	**E-E'**	w^-;esg-GAL4 UAS-gfp/+; Su(H)GBE-lacZ,tubGAL80^{TS}/UAS-mcherryRNAi and

		w^{-};esg-GAL4 UAS-gfp/dwdr4RNAi$^{(P)}$; Su(H)GBE-lacZ tubGAL80TS/+
	**F**	*w^{-};esg-GAL4 UAS-gfp/dwdr4RNAi$^{(P)}$; Su(H)GBE-lacZ tubGAL80TS/+ and dmyc4/+;esg-Gal4,UAS-gfp/dwdr4RNAi$^{(P)}$; Su(H)GBE-lacZ tubGAL80TS/+*
	**F'**	*w^{-};esg-GAL4 UAS-gfp/+; Su(H)GBE-lacZ,tubGAL80TS/UAS-mcherryRNAi and w^{-};esg-GAL4 UAS-gfp/dwdr4RNAi$^{(P)}$; Su(H)GBE-lacZ tubGAL80TS/+ and dmyc4/+;esg-Gal4,UAS-gfp/dwdr4RNAi$^{(P)}$; Su(H)GBE-lacZ tubGAL80TS/+ and dmyc4/+;esg-Gal4,UAS-gfp/+; Su(H)GBE-lacZ tubGAL80TS/+*
**Figure 6**	**A**	*w^{-};esg-GAL4/UAS-mcD8::gfp and wh7/wh7;esg-GAL4/UAS-mcD8::gfp*
	**B**	*wh7/wh7;esg-Gal4/UAS-mcD8::gfp and wh7/wh7;esg-GAL4/+;UAS-mcD8::gfp/UAS-hepRNAi*
	**B'**	*w^{-};esg-GAL4/UAS-mcD8::gfp and wh7/wh7;esg-GAL4/UAS-mcD8::gfp and wh7/wh7;esg-GAL4/+;UAS-mcD8::gfp/UAS-hepRNAi*
	**C-C'**	*w^{-};esg-Gal4,Uas-gfp/ dwdr4RNAi$^{(P)}$; Su(H)GBE-lacZ,tubGal80TS/+*
	**D**	*wh7/wh7 ;esg-GAL4 UAS-gfp/+;Su(H)GBE-lacZ tubGAL80TS/+ and wh7/wh7 ;esg-GAL4,UAS-gfp/+;Su(H)GBE-lacZ tubGAL80TS/UAS-hepRNAi*
	**D'**	*w^{-};esg-GAL4 UAS-gfp/+; Su(H)GBE-lacZ,tubGAL80TS/UAS-mcherryRNAi and wh7/wh7 ;esg-GAL4 UAS-gfp/+;Su(H)GBE-lacZ tubGAL80TS/+ and wh7/wh7 ;esg-GAL4,UAS-gfp/+;Su(H)GBE-lacZ tubGAL80TS/UAS-hepRNAi*
**Figure 7**	**A**	*w^{-};tub-GAL80^{TS}/+;Dl-GAL4 UAS-gfp/UAS-mcherryRNAi and w^{-};tub-GAL80TS/+;Dl-GAL4 UAS-gfp/let-7-12p decoy*
	**A'**	*w^{-};tub-GAL80TS/+;Dl-GAL4 UAS-gfp/UAS-mcherryRNAi and w^{-};tub-GAL80TS/dwdr4RNAi$^{(P)}$;Dl-GAL4 UAS-gfp/+ and w^{-};tub-GAL80TS/+;Dl-GAL4 UAS-gfp/let-7-12p decoy and w^{-};tub-GAL80TS/mettl1RNAi;Dl-GAL4 UAS-gfp/+ and w^{-};tub-GAL80TS/+;Dl-GAL4 UAS-gfp/let-7 sponge*
	**B**	*w^{-};esg-GAL4 UAS-gfp/+; Su(H)GBE-lacZ,tubGAL80TS/UAS-mcherryRNAi and w^{-};esg-GAL4 UAS-gfp/dwdr4RNAi$^{(P)}$; Su(H)GBE-lacZ tubGAL80TS/+*
	**B'**	*w^{-};esg-GAL4 UAS-gfp/+; Su(H)GBE-lacZ,tubGAL80TS/UAS-mcherryRNAi*
	**C-E**	*w^{-};esg-Gal4 UAS-gfp/dwdr4RNAi; Su(H)GBE-lacZ,tubGAL80TS/+ and w^{-};esg-GAL4 UAS-gfp/dwdr4RNAi$^{(P)}$; Su(H)GBE-lacZ,tubGAL80TS/UAS-let7*
	**C'-E'**	*w^{-};esg-GAL4 UAS-gfp/+; Su(H)GBE-lacZ,tubGAL80TS/UAS-mcherryRNAi and w^{-};esg-Gal4 UAS-gfp/dwdr4RNAi$^{(P)}$; Su(H)GBE-lacZ,tubGAL80TS/+ and w^{-};esg-GAL4 UAS-gfp/dwdr4RNAi$^{(P)}$; Su(H)GBE-lacZ,tubGAL80TS/UAS-let7*
**Fig. EV1**	**A-D**	*yw* and *wh7/wh7* and *wh7/wh7;dwdr4-gfp/+*
	**E-F**	*yw and wh7/wh7*
**Fig. EV2**	**A**	*w^{-};esg-GAL4/UAS-mcD8::gfp* and *wh7/wh7;esg-GAL4/UAS-mcD8::gfp*
	**B**	*FRT19A/hs-flpGAL80FRT19A;actGAL4/UAS-mcD8::gfp and wh7FRT19A/hs-flpGAL80FRT19A;actGAL4/UAS-mcD8::gfp*

	**C**	w^-;esg-GAL4 UAS-gfp/ +; Su(H)GBE-lacZ,tubGAL80^{TS}/UAS-mcherryRNAi; and w^-;esg-GAL4 UAS-gfp/ dwdr4RNAi$^{(P)}$; Su(H)GBE-lacZ tubGAL80TS/+
	**D**	w^-;esg-GAL4/UAS-egfpRNAi and w^-;esg-GAL4/UAS-dwdr4RNAi$^{(P)}$
	**E**	w^-;Su(H)GBE-GAL4 UAS-mcD8::gfp,tubGAL80^{TS}/+;UAS-mcherryRNAi/+ and w^-;Su(H)GBE-GAL4 UAS-mcD8::gfp,tubGAL80TS/UAS-dwdr4RNAi$^{(P)}$;+/+
	**F**	w^-;NP1-GAL4/UAS-egfpRNAi and w^-;NP1-GAL4/UAS-dwdr4RNAi$^{(P)}$
	**G**	w^-;tub-GAL80^{TS}/+;Dl-GAL4 UAS-gfp/UAS-mcherryRNAi and w^-;tub-GAL80TS/dwdr4RNAi$^{(P)}$;Dl-GAL4 UAS-gfp/+ and w^-;How-GAL4/+;UAS-mcherryRNAi/+and w^-;How-GAL4/dwdr4RNAi$^{(P)}$;+/+ and w^-;How-GAL4/dwdr4RNAi$^{(t)}$;+/+
	**H**	w^-;esg-GAL4/UAS-lucRNAi and w^-;esg-GAL4/UAS-dwdr4RNAi$^{(P)}$
**Fig. EV3**	**A**	FRT19A/hs-flpGAL80FRT19A;actGAL4/UAS-mcD8::gfp and wh7FRT19A/hs-flpGAL80FRT19A;actGAL4/UAS-mcD8::gfp
	**B**	w^-;tub-GAL80TS/dwdr4RNAi$^{(P)}$;Dl-GAL4 UAS-gfp/+
	**B'**	w^-;tub-GAL80TS/+;Dl-GAL4 UAS-gfp/UAS-mcherryRNAi and w^-;tub-GAL80TS/dwdr4RNAi$^{(P)}$;Dl-GAL4 UAS-gfp/+
	**C**	w^-;esg-GAL4 UAS-gfp/dwdr4RNAi$^{(P)}$; Su(H)GBE-lacZ tubGAL80TS/+ and dmyc4/+;esg-Gal4,UAS-gfp/dwdr4RNAi$^{(P)}$; Su(H)GBE-lacZ tubGAL80TS/+
	**C'**	w^-;esg-GAL4 UAS-gfp/+; Su(H)GBE-lacZ,tubGAL80TS/UAS-mcherryRNAi and w^-;esg-GAL4 UAS-gfp/dwdr4RNAi$^{(P)}$; Su(H)GBE-lacZ tubGAL80TS/+ and dmyc4/+;esg-Gal4,UAS-gfp/dwdr4RNAi$^{(P)}$; Su(H)GBE-lacZ tubGAL80TS/+ and dmyc4/+;esg-Gal4,UAS-gfp/+; Su(H)GBE-lacZ tubGAL80TS/+
**Fig. EV4**	**A**	yw and wh7/wh7
	**B-B'**	yw;esg-Gal4/Uas-mcD8::gfp and wh7/wh7;esg-Gal4/Uas-mcD8::gfp
	**C**	yw;+/+;puc-lacZ/+ and wh7/wh7;+/+;puc-lacZ/+
	**D-E**	w^-;esg-Gal4 UAS-gfp/dwdr4RNAi$^{(P)}$; Su(H)GBE-lacZ,tubGAL80TS/+ and w^-;esg-GAL4 UAS-gfp/dwdr4RNAi$^{(P)}$; Su(H)GBE-lacZ,tubGAL80TS/UAS-Catalase
	**D'-E'**	w^-;esg-GAL4 UAS-gfp/+; Su(H)GBE-lacZ,tubGAL80TS/UAS-mcherryRNAi and w^-;esg-GAL4 UAS-gfp/dwdr4RNAi$^{(P)}$; Su(H)GBE-lacZ tubGAL80TS/+ and w^-;esg-GAL4 UAS-gfp/dwdr4RNAi$^{(P)}$; Su(H)GBE-lacZ,tubGAL80TS/UAS-Catalase
	**F-G**	w^-;esg-GAL4 UAS-gfp/dwdr4RNAi$^{(P)}$; Su(H)GBE-lacZ tubGAL80TS/+ and dmyc4/+;esg-GAL4 UAS-gfp/dwdr4RNAi$^{(P)}$; Su(H)GBE-lacZ tubGAL80TS/+
	**F'-G'**	w^-;esg-GAL4 UAS-gfp/+; Su(H)GBE-lacZ,tubGAL80TS/UAS-mcherryRNAi and w^-;esg-GAL4 UAS-gfp/dwdr4RNAi$^{(P)}$; Su(H)GBE-lacZ tubGAL80TS/+ and dmyc4/+;esg-Gal4,UAS-gfp/dwdr4RNAi$^{(P)}$; Su(H)GBE-lacZ tubGAL80TS/+
**Fig. EV5**	**A-A'**	w^-;esg-GAL4 UAS-gfp/+; Su(H)GBE-lacZ tubGAL80^{TS}/UAS-mcherryRNAi; and w-;esg-GAL4 UAS-gfp/dwdr4RNAi$^{(P)}$; Su(H)GBE-lacZ tubGAL80TS/+

	**B-B'**	*w^-;esg-GAL4,UAS-gfp/+; Su(H)GBE-lacZ tubGAL80^{TS}/UAS-mcherryRNAi; and w^-;esg-Gal4 UAS-gfp/mettl1RNAi; Su(H)GBE-lacZ tubGAL80TS/+*
	**C-C'**	*w^-;esg-GAL4/dwdr4-gfp; UAS-Mettl1-V5/+*
	**D**	*wh7/wh7;esg-GAL4/UAS-mcD8::gfp and wh7/wh7;esg-GAL4/UAS-mcD8::gfp; UAS-hWDR4/+*
	**D'**	*yw;esg-Gal4/Uas-mcD8::gfp and wh7/wh7;esg-GAL4/UAS-mcD8::gfp and wh7/wh7;esg-GAL4/UAS-mcD8::gfp; UAS-hWDR4/+*
	**E**	*w^-;esg-GAL4 UAS-gfp/+; Su(H)GBE-lacZ,tubGAL80TS/UAS-mcherryRNAi and wh7/wh7 ;esg-GAL4 UAS-gfp/+;Su(H)GBE-lacZ tubGAL80TS/+ and wh7/wh7 ;esg-GAL4,UAS-gfp/+;Su(H)GBE-lacZ tubGAL80TS/UAS-hWDR4*
	**F**	*w^-;tub-GAL80TS/+;Dl-GAL4 UAS-gfp/UAS-mcherryRNAi and w^-;tub-GAL80TS/mettl1RNAi;Dl-GAL4 UAS-gfp/+ and w^-;tub-GAL80TS/mettl1RNAi;Dl-GAL4 UAS-gfp/UAS-hMETTL1 and w^-;tub-GAL80TS/mettl1RNAi;Dl-GAL4 UAS-gfp/UAS-hMETTL1^{KD}*
	**F'**	*w^-;tub-GAL80TS/+;Dl-GAL4 UAS-gfp/UAS-mcherryRNAi and w^-;tub-GAL80TS/dwdr4RNAi$^{(P)}$;Dl-GAL4 UAS-gfp/+ and w^-;tub-GAL80TS/mettl1RNAi;Dl-GAL4 UAS-gfp/+ and w^-;tub-GAL80TS/mettl1RNAi;Dl-GAL4 UAS-gfp/UAS-hMETTL1 and w^-;tub-GAL80TS/mettl1RNAi;Dl-GAL4 UAS-gfp/UAS-hMETTL1^{KD}*
**Fig. EV6**	**A**	*yw and wh7/wh7*
	**B**	*w^-;tub-GAL80^{TS}/+;Dl-GAL4 UAS-gfp/let7-lacZ and w^-;tub-GAL80TS/dwdr4RNAi$^{(P)}$;Dl-GAL4 UAS-gfp/let7-lacZ*
	**C-C'**	*w^-;tub-GAL80^{TS}/+;Dl-GAL4 UAS-gfp/UAS-mcherryRNAi and w^-;tub-GAL80TS/+;Dl-GAL4 UAS-gfp/let-7-12p decoy*
