# Supplementary figures and images for: Wdr4 regulates ribosome biogenesis and intestinal homeostasis via let-7 (part 1 of 2)
Source: EMBO Rep. 2026 Feb 9;27(8):1870–903. doi: 10.1038/s44319-026-00701-y (PMC13121520; doi:10.1038/s44319-026-00701-y)

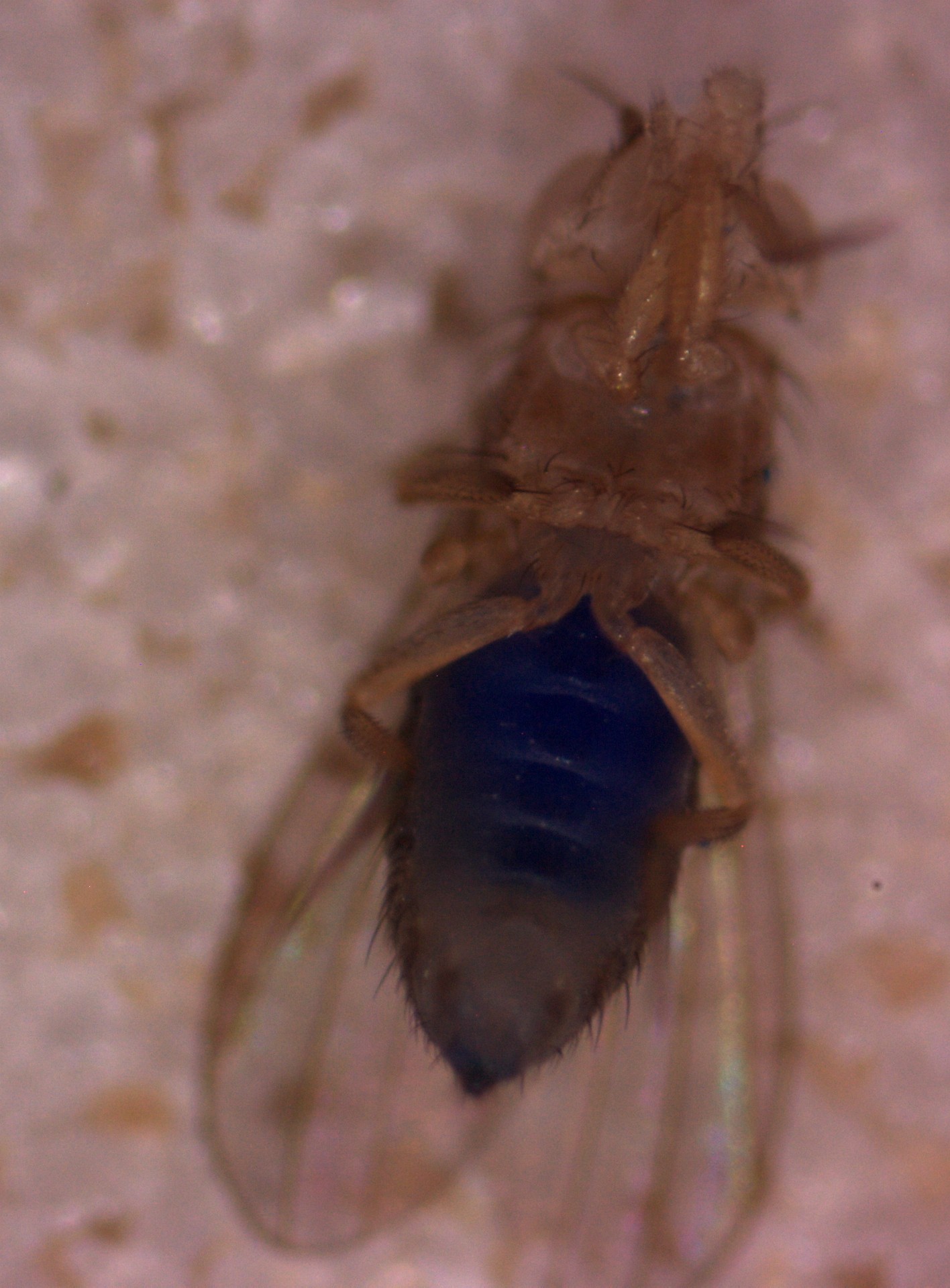

Supplement: Supplementary file 4 — Source data Fig. 1 [file 44319_2026_701_MOESM4_ESM.zip › Fig. 1/Fig. 1A/wh7.jpg]

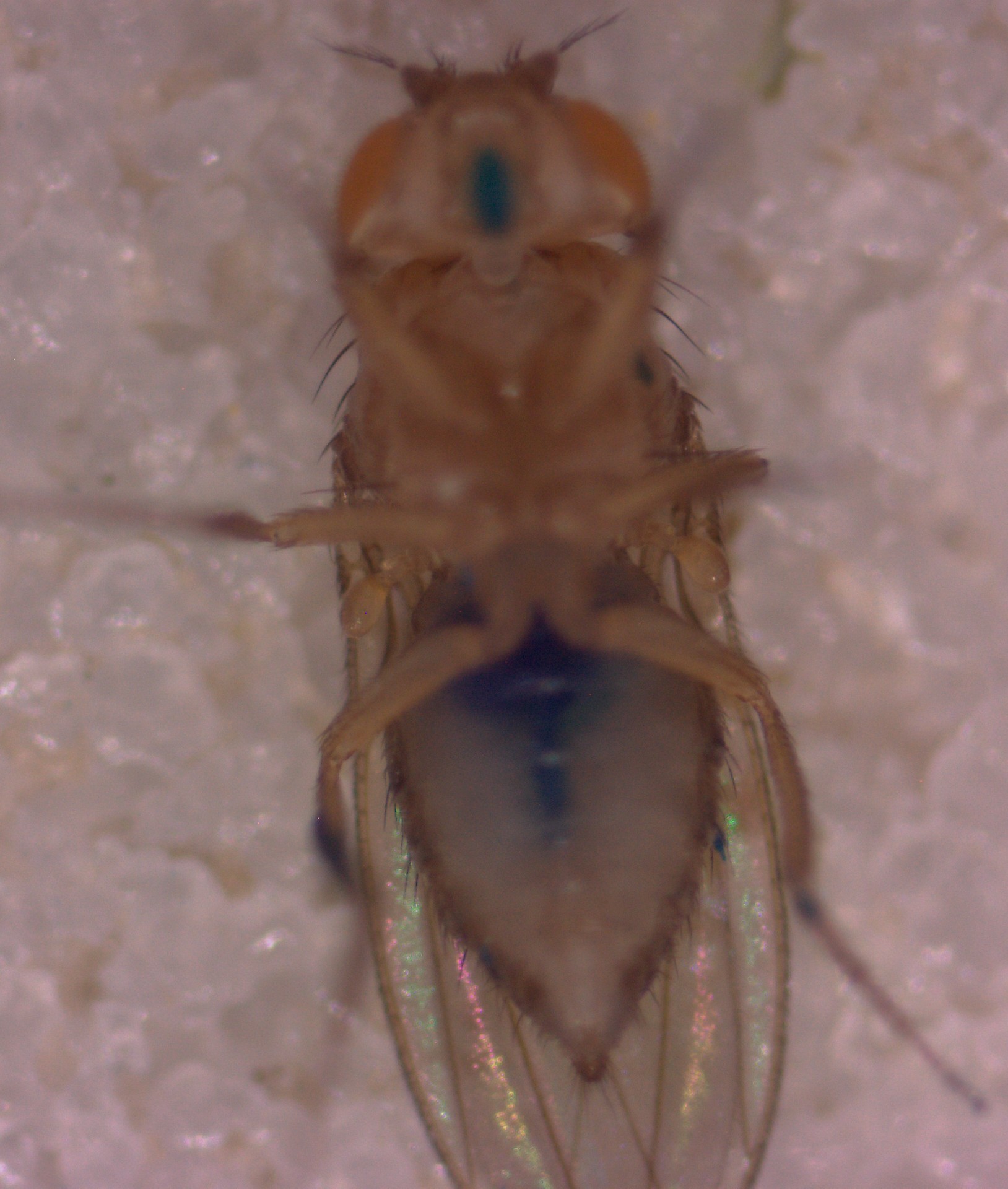

Supplement: Supplementary file 4 — Source data Fig. 1 [file 44319_2026_701_MOESM4_ESM.zip › Fig. 1/Fig. 1A/wh7;wh-gfp.jpg]

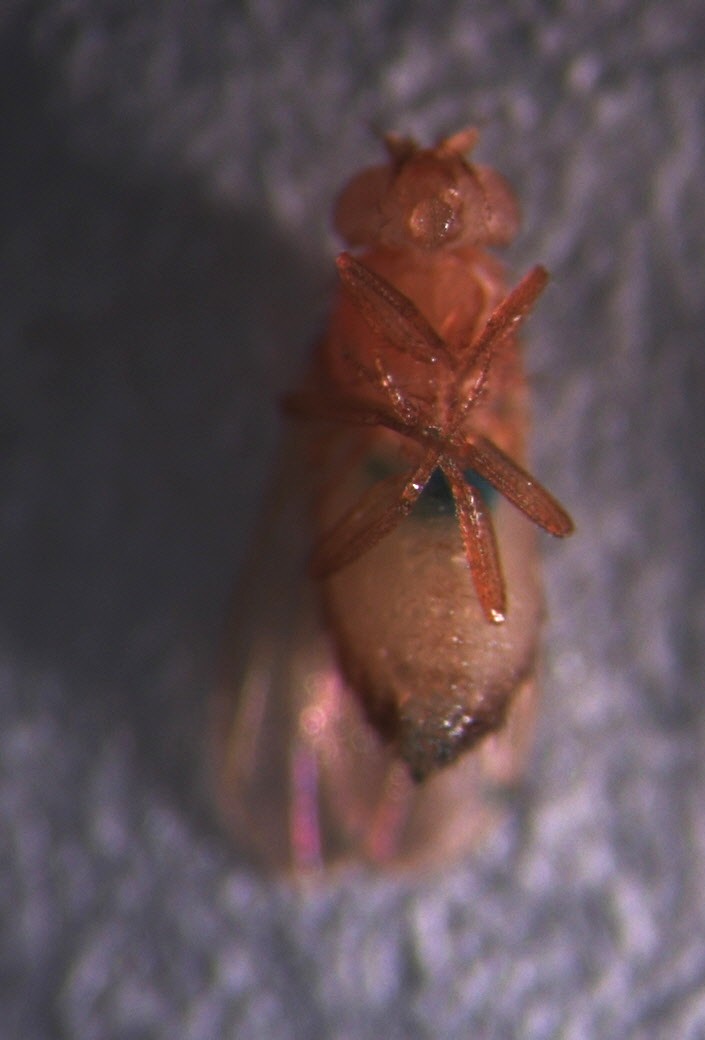

Supplement: Supplementary file 4 — Source data Fig. 1 [file 44319_2026_701_MOESM4_ESM.zip › Fig. 1/Fig. 1A/yw.JPG]

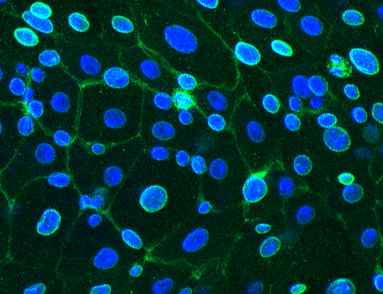

Supplement: Supplementary file 4 — Source data Fig. 1 [file 44319_2026_701_MOESM4_ESM.zip › Fig. 1/Fig. 1B- B'/wh7_Dlg+LamC+DAPI.tif]

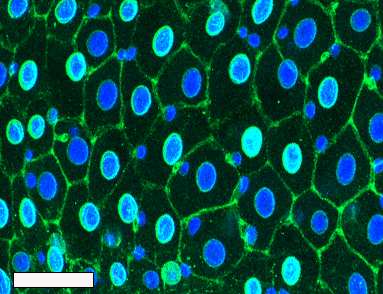

Supplement: Supplementary file 4 — Source data Fig. 1 [file 44319_2026_701_MOESM4_ESM.zip › Fig. 1/Fig. 1B- B'/WT_Dlg+LamC+DAPI.tif]

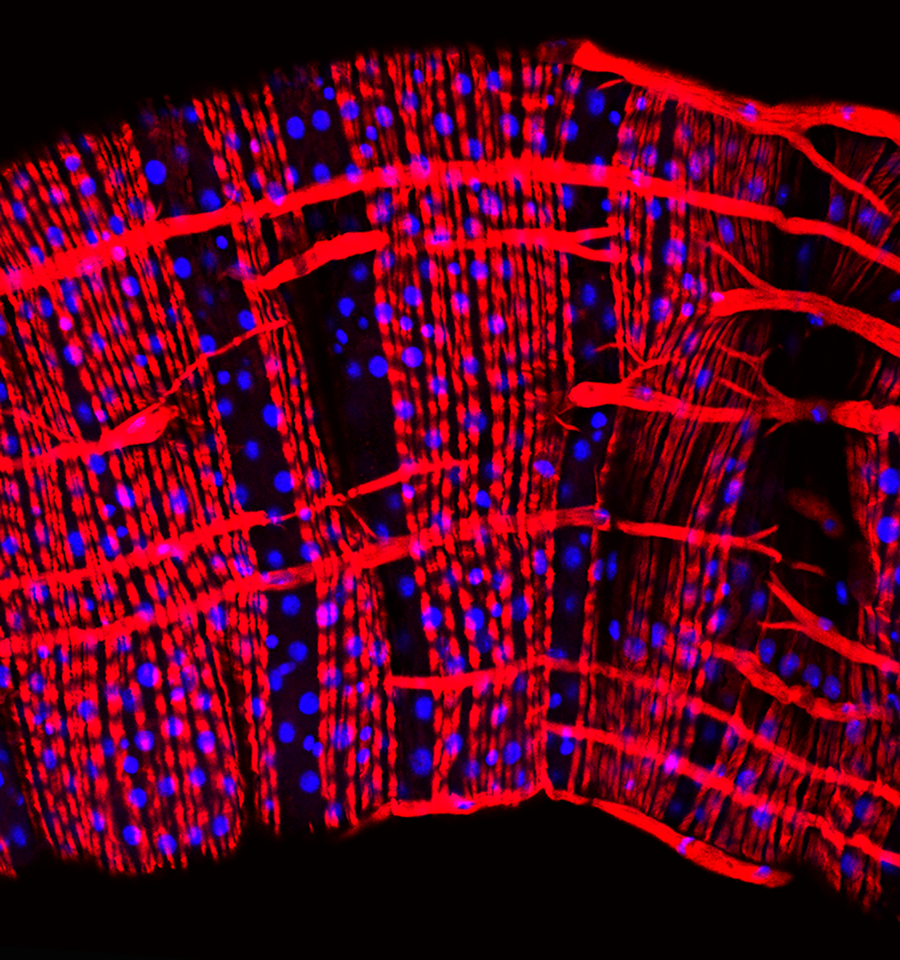

Supplement: Supplementary file 4 — Source data Fig. 1 [file 44319_2026_701_MOESM4_ESM.zip › Fig. 1/Fig. 1C/wh7_DAPI+Phalloidin.tif]

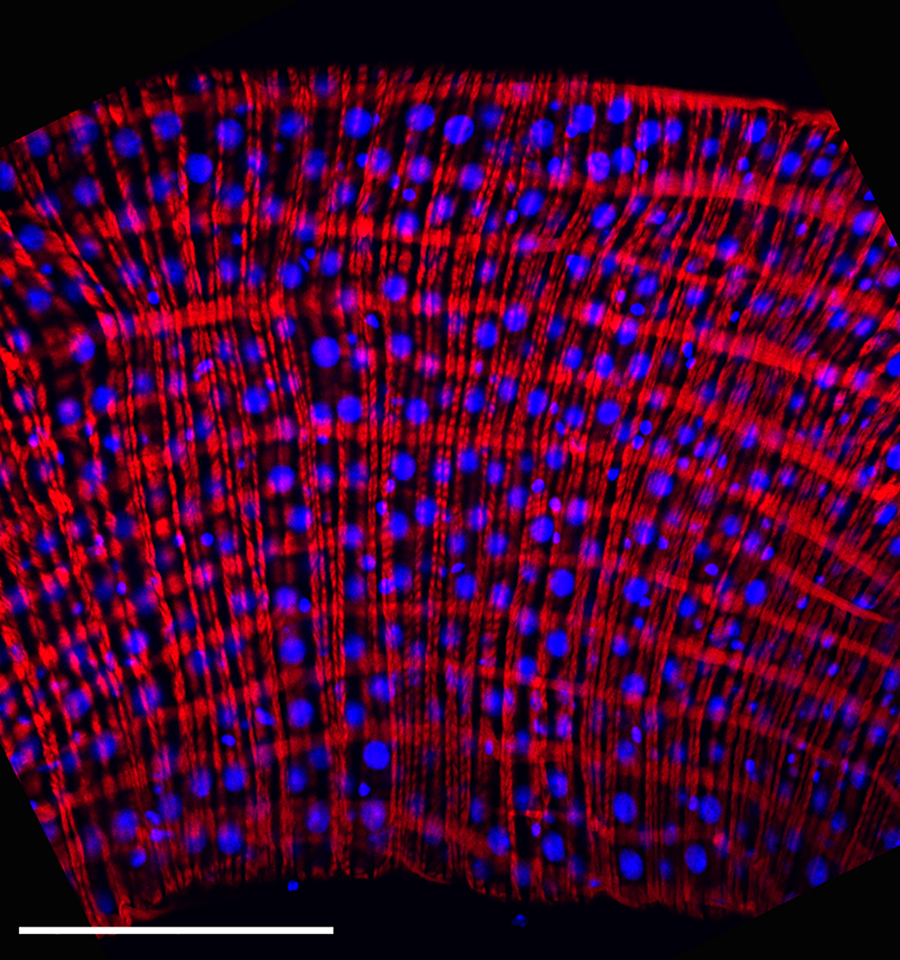

Supplement: Supplementary file 4 — Source data Fig. 1 [file 44319_2026_701_MOESM4_ESM.zip › Fig. 1/Fig. 1C/WT_DAPI+Phalloidin.tif]

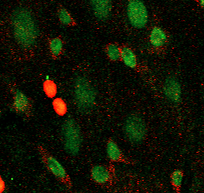

Supplement: Supplementary file 4 — Source data Fig. 1 [file 44319_2026_701_MOESM4_ESM.zip › Fig. 1/Fig. 1E/GFP+Hdc+Pros.tif]

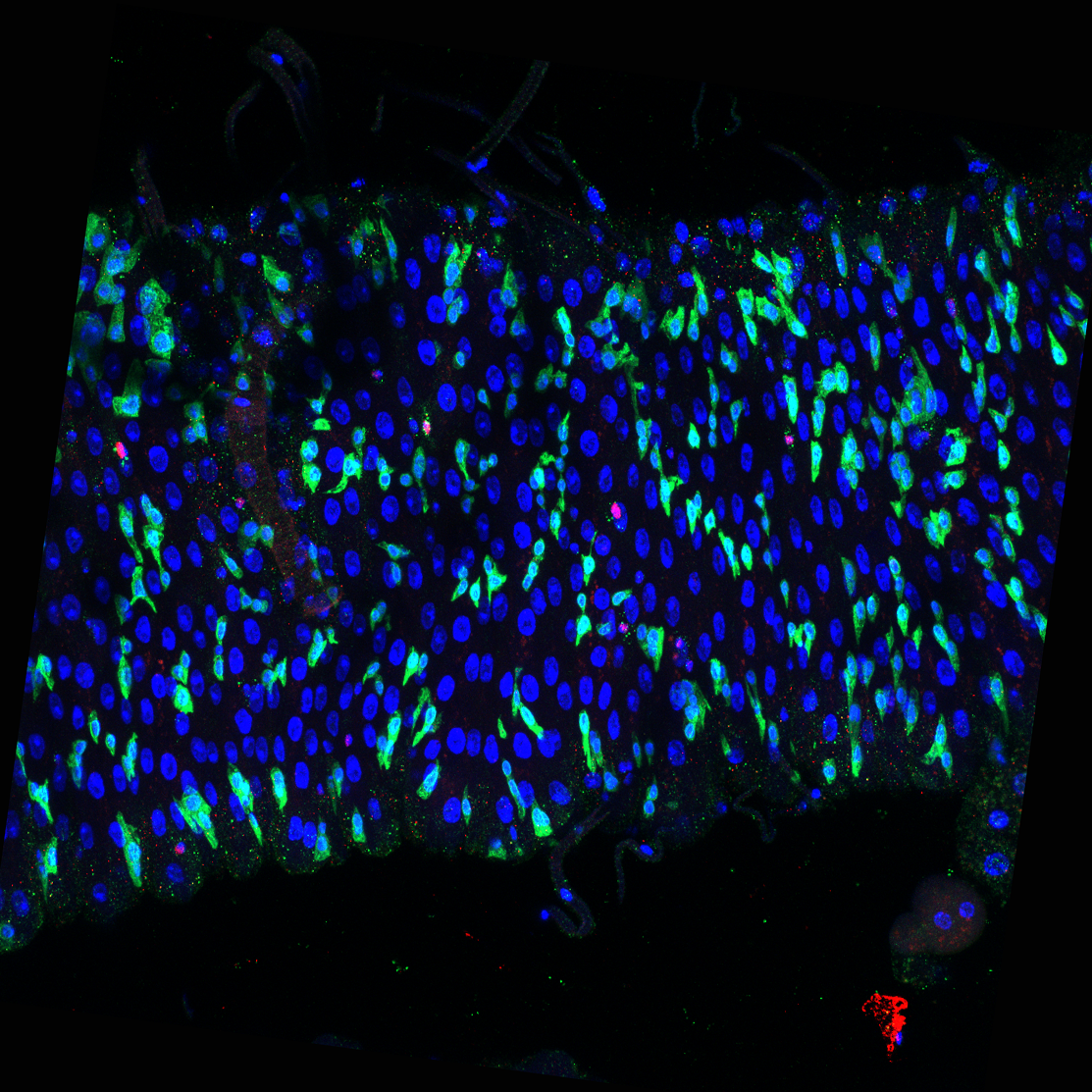

Supplement: Supplementary file 5 — Source data Fig. 2 [file 44319_2026_701_MOESM5_ESM.zip › Fig. 2/Fig. 2A-A'/Ind. ISCs_wh7_GFP+Pros+DAPI.tif]

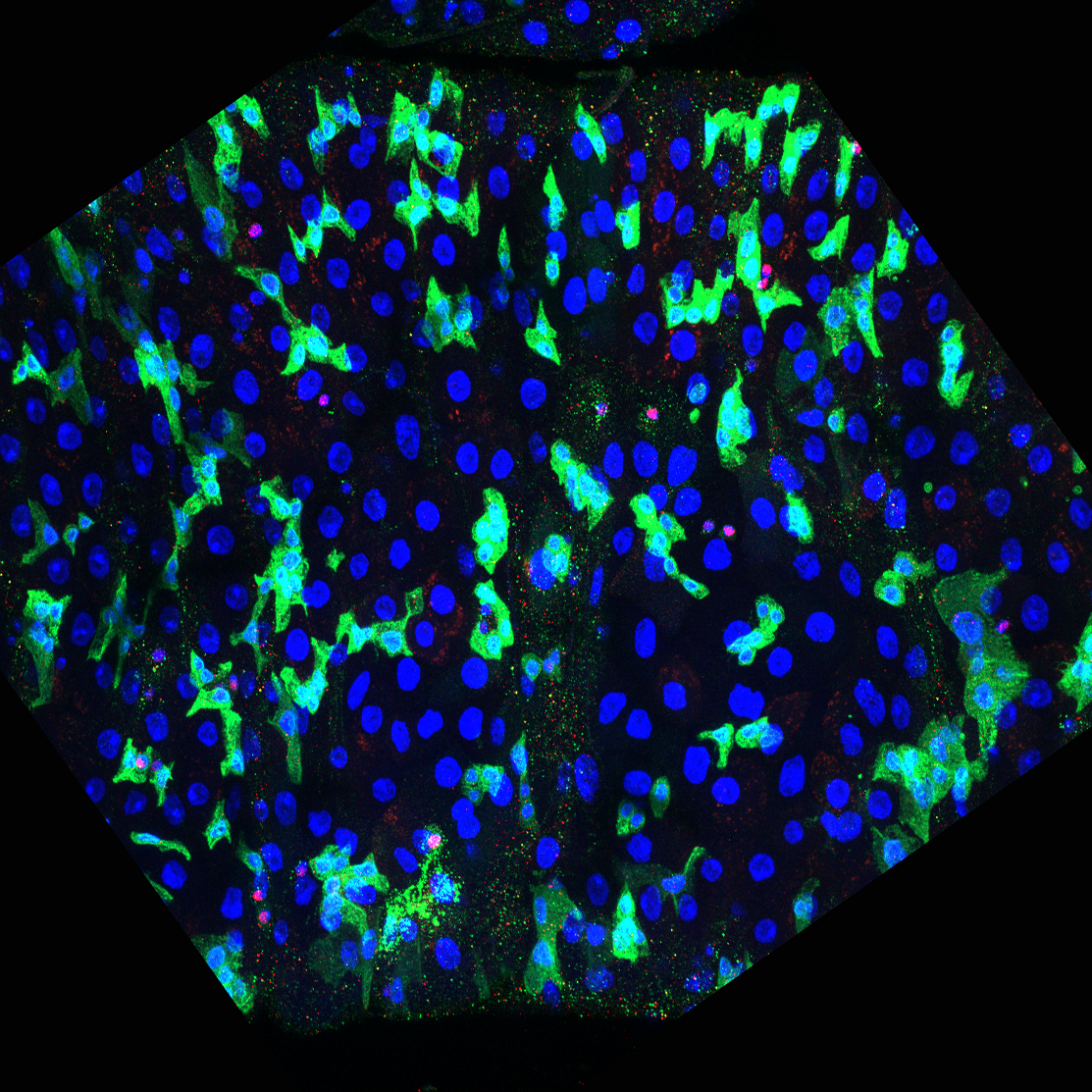

Supplement: Supplementary file 5 — Source data Fig. 2 [file 44319_2026_701_MOESM5_ESM.zip › Fig. 2/Fig. 2A-A'/Intermediate_wh7_GFP+Pros+DAPI.tif]

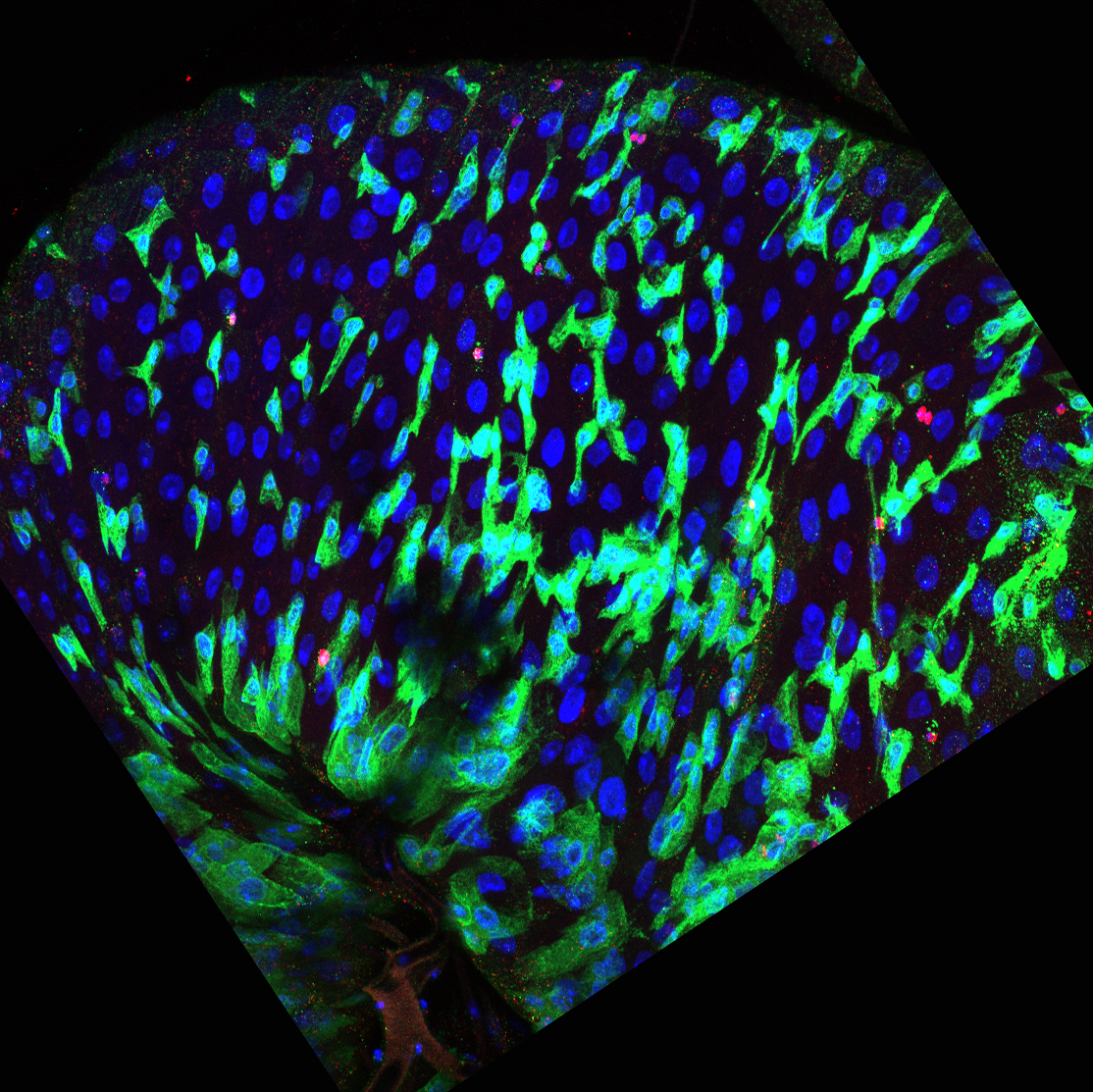

Supplement: Supplementary file 5 — Source data Fig. 2 [file 44319_2026_701_MOESM5_ESM.zip › Fig. 2/Fig. 2A-A'/Strong_wh7_GFP+Pros+DAPI.tif]

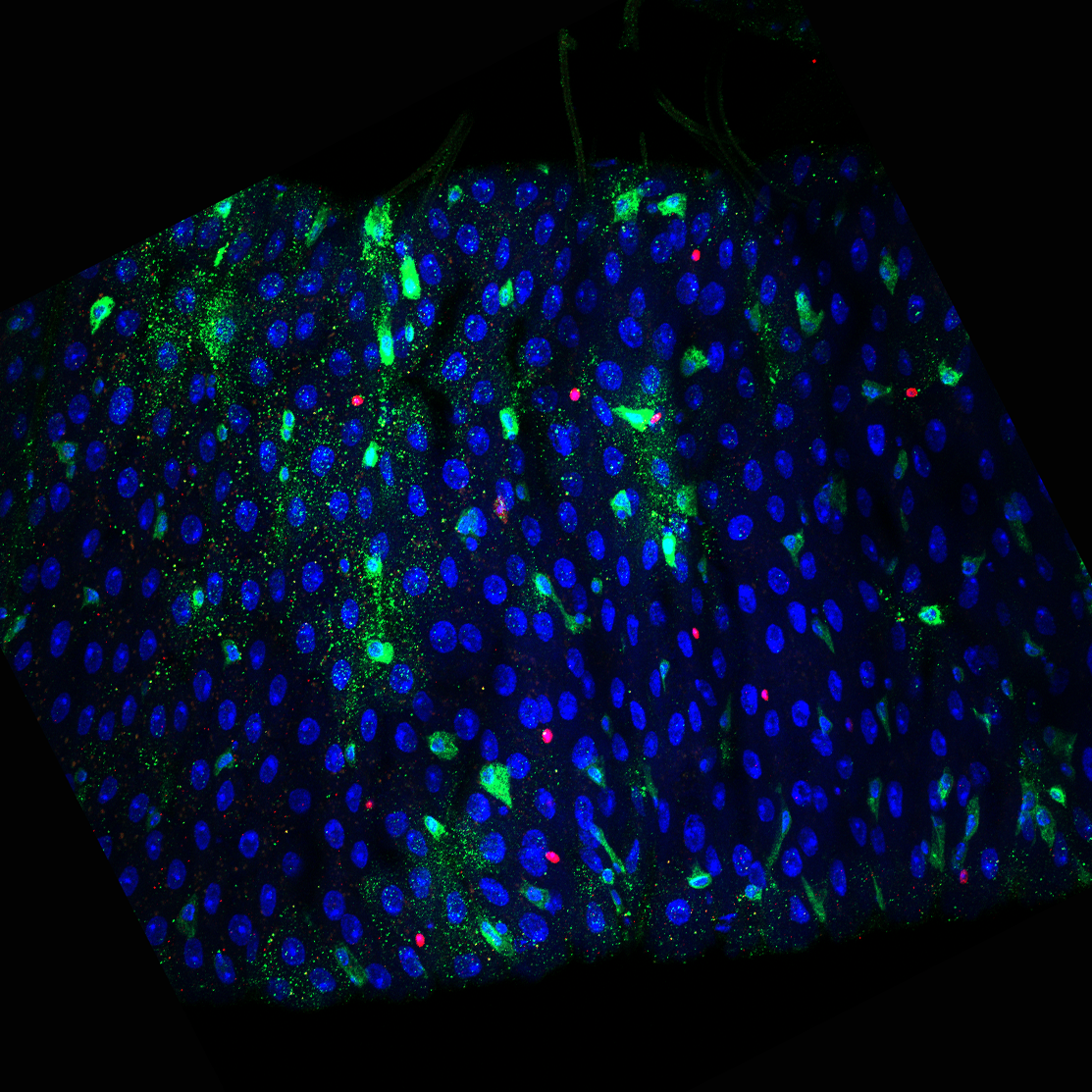

Supplement: Supplementary file 5 — Source data Fig. 2 [file 44319_2026_701_MOESM5_ESM.zip › Fig. 2/Fig. 2A-A'/WT_GFP+Pros+DAPI.tif]

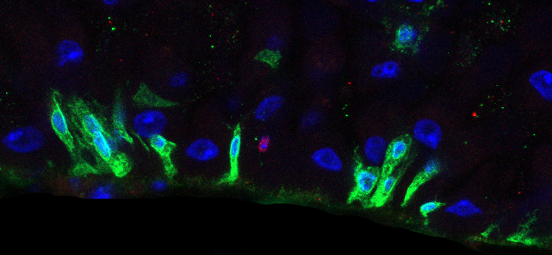

Supplement: Supplementary file 5 — Source data Fig. 2 [file 44319_2026_701_MOESM5_ESM.zip › Fig. 2/Fig. 2B/wh7_GFP+DAPI+pros.tif]

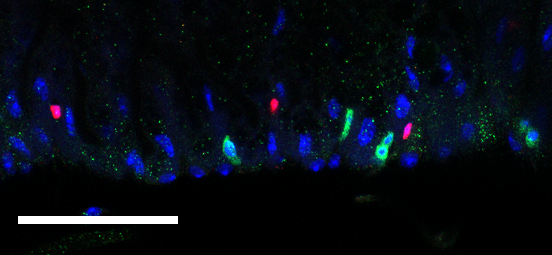

Supplement: Supplementary file 5 — Source data Fig. 2 [file 44319_2026_701_MOESM5_ESM.zip › Fig. 2/Fig. 2B/WT_GFP+Pros+DAPI.tif]

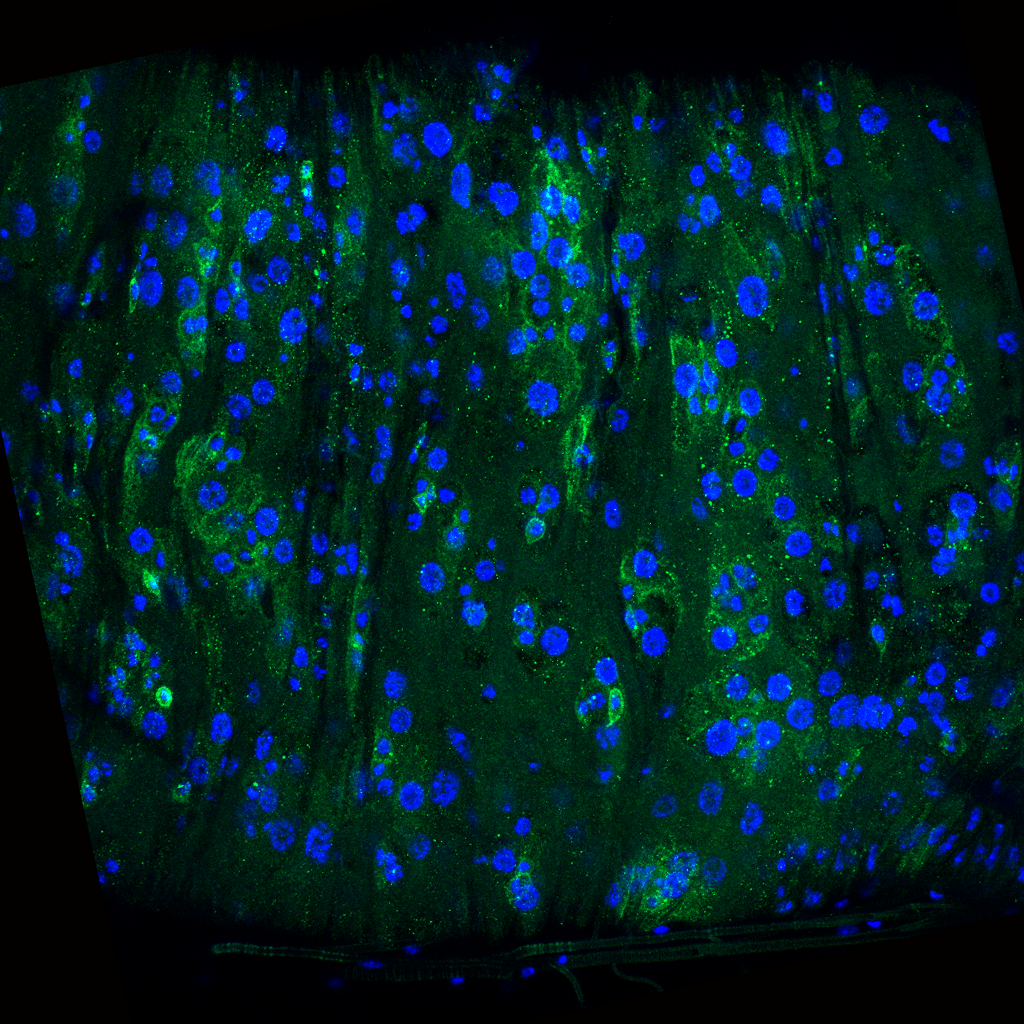

Supplement: Supplementary file 5 — Source data Fig. 2 [file 44319_2026_701_MOESM5_ESM.zip › Fig. 2/Fig. 2D/wh7_Dl+DAPI.tif]

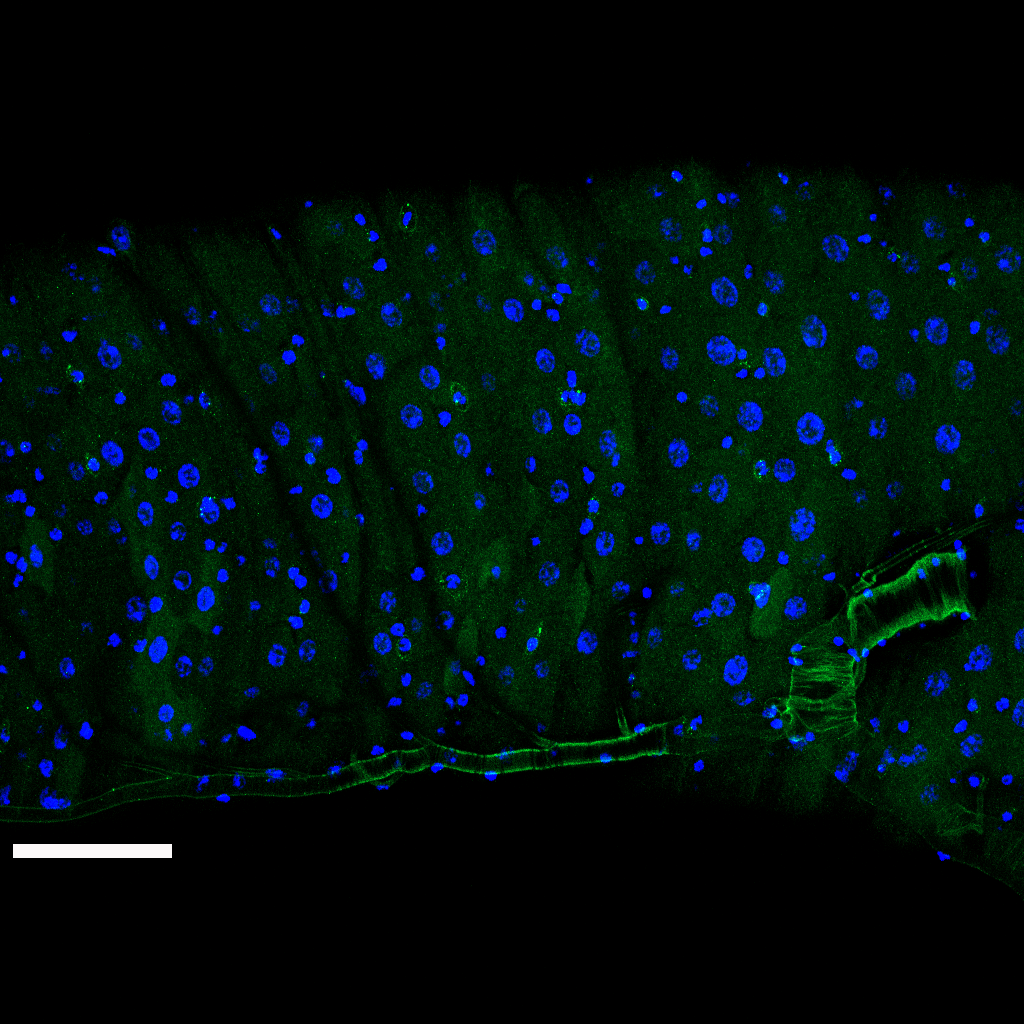

Supplement: Supplementary file 5 — Source data Fig. 2 [file 44319_2026_701_MOESM5_ESM.zip › Fig. 2/Fig. 2D/WT_Dl+DAPI.tif]

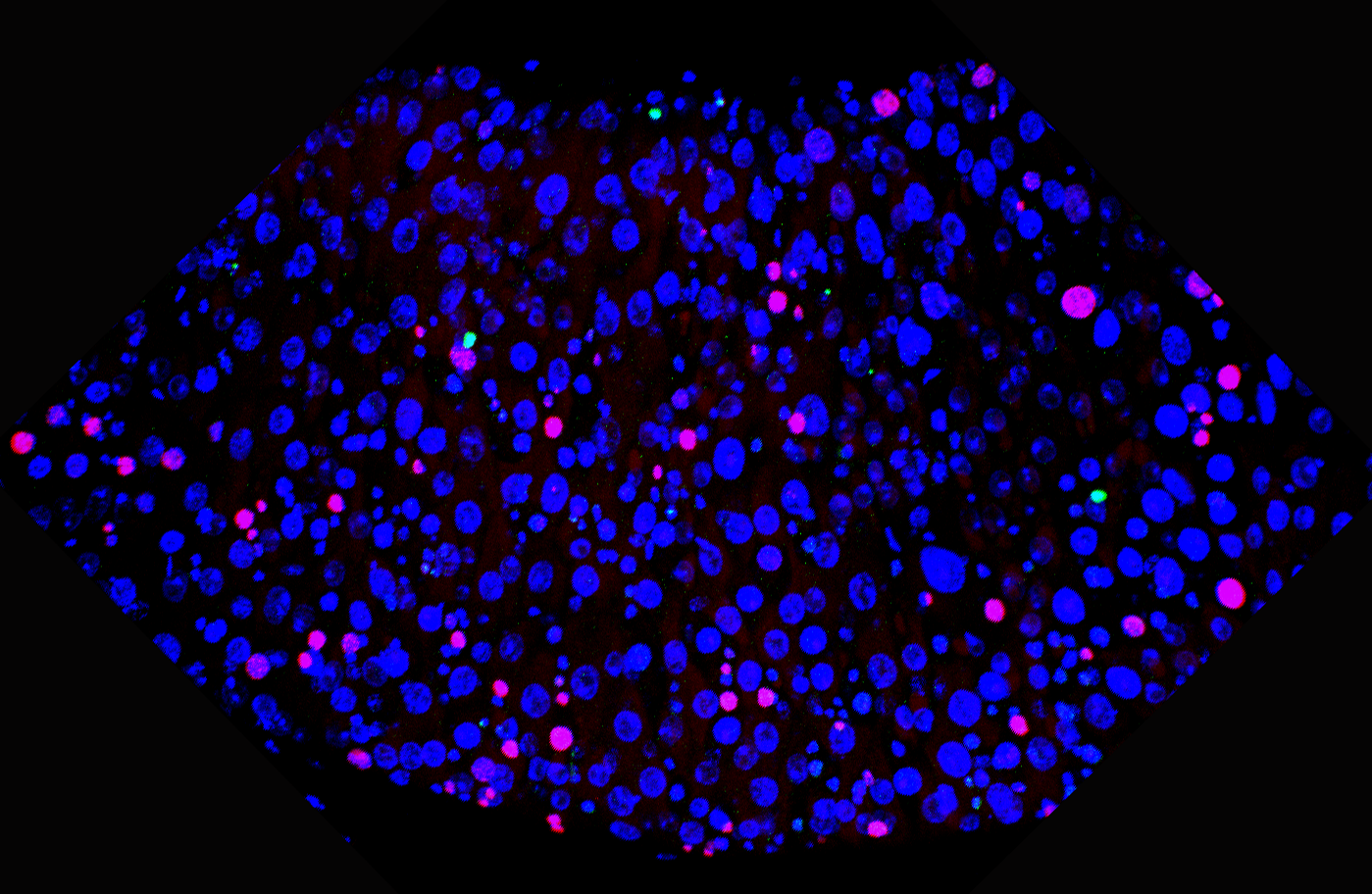

Supplement: Supplementary file 5 — Source data Fig. 2 [file 44319_2026_701_MOESM5_ESM.zip › Fig. 2/Fig. 2E-E'/wh7_phh3+EdU+DAPI.tif]

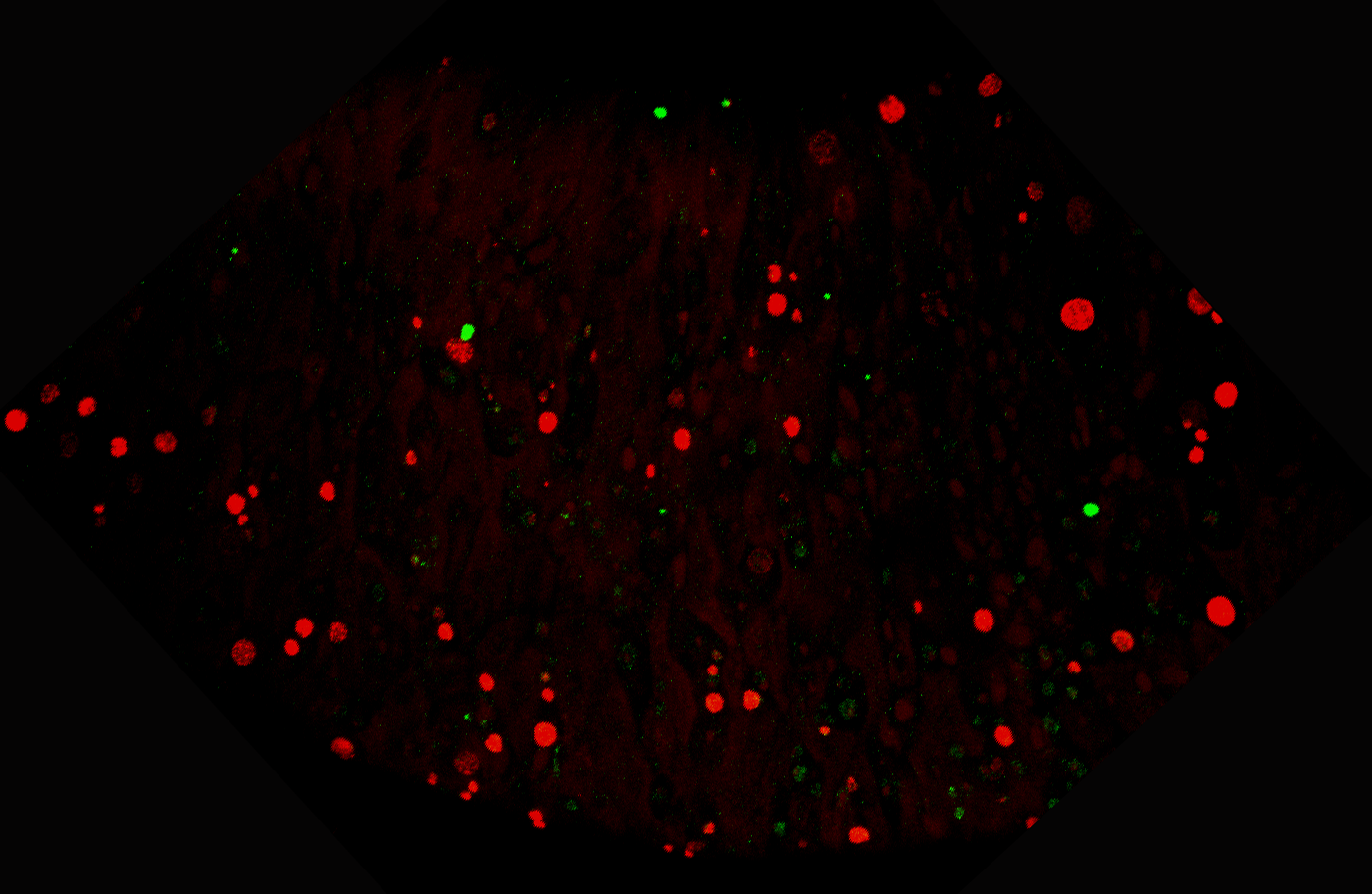

Supplement: Supplementary file 5 — Source data Fig. 2 [file 44319_2026_701_MOESM5_ESM.zip › Fig. 2/Fig. 2E-E'/wh7_phh3+EdU.tif]

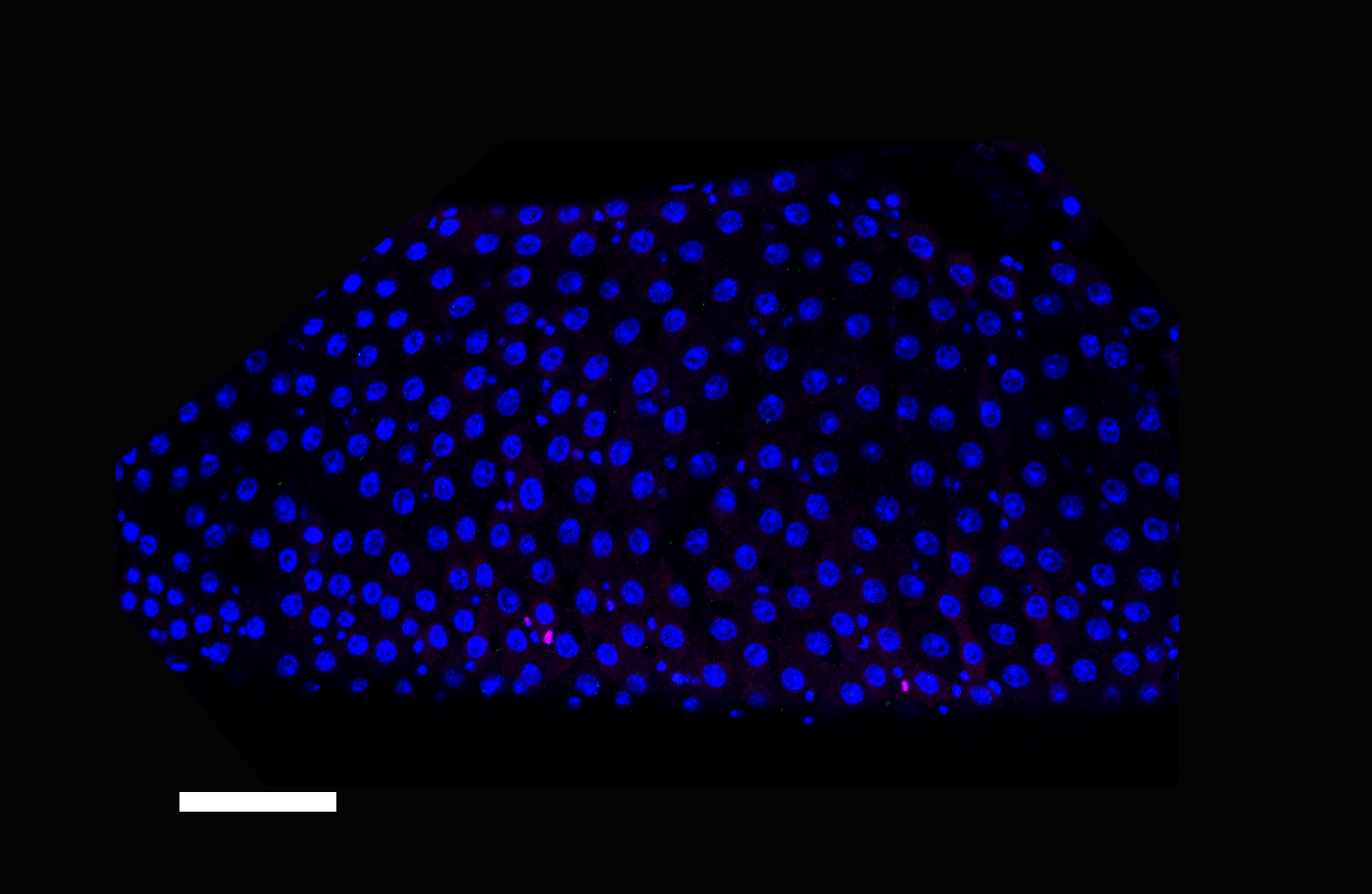

Supplement: Supplementary file 5 — Source data Fig. 2 [file 44319_2026_701_MOESM5_ESM.zip › Fig. 2/Fig. 2E-E'/WT_phh3+EdU+DAPI.tif]

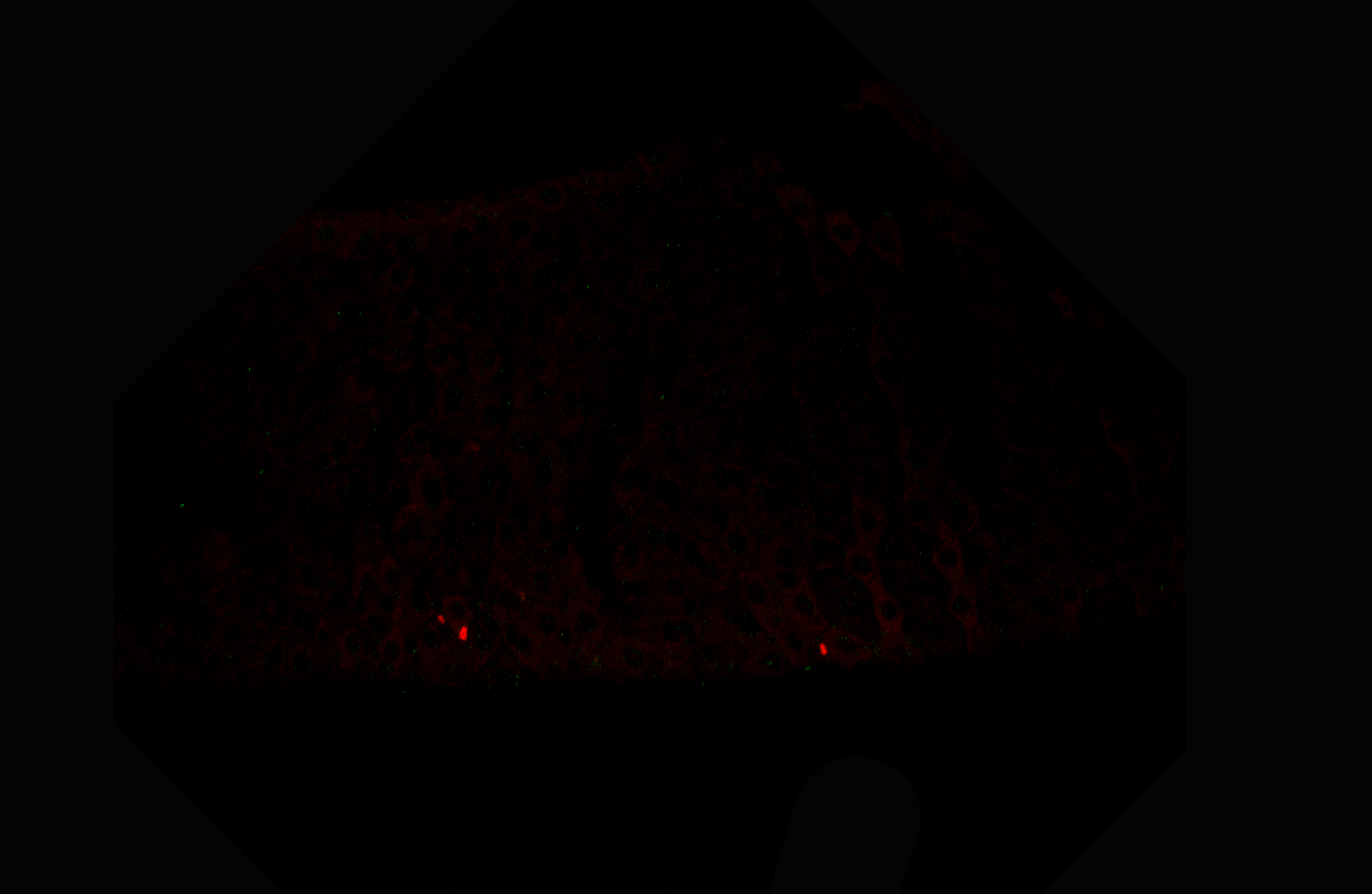

Supplement: Supplementary file 5 — Source data Fig. 2 [file 44319_2026_701_MOESM5_ESM.zip › Fig. 2/Fig. 2E-E'/WT_phh3+edU.tif]

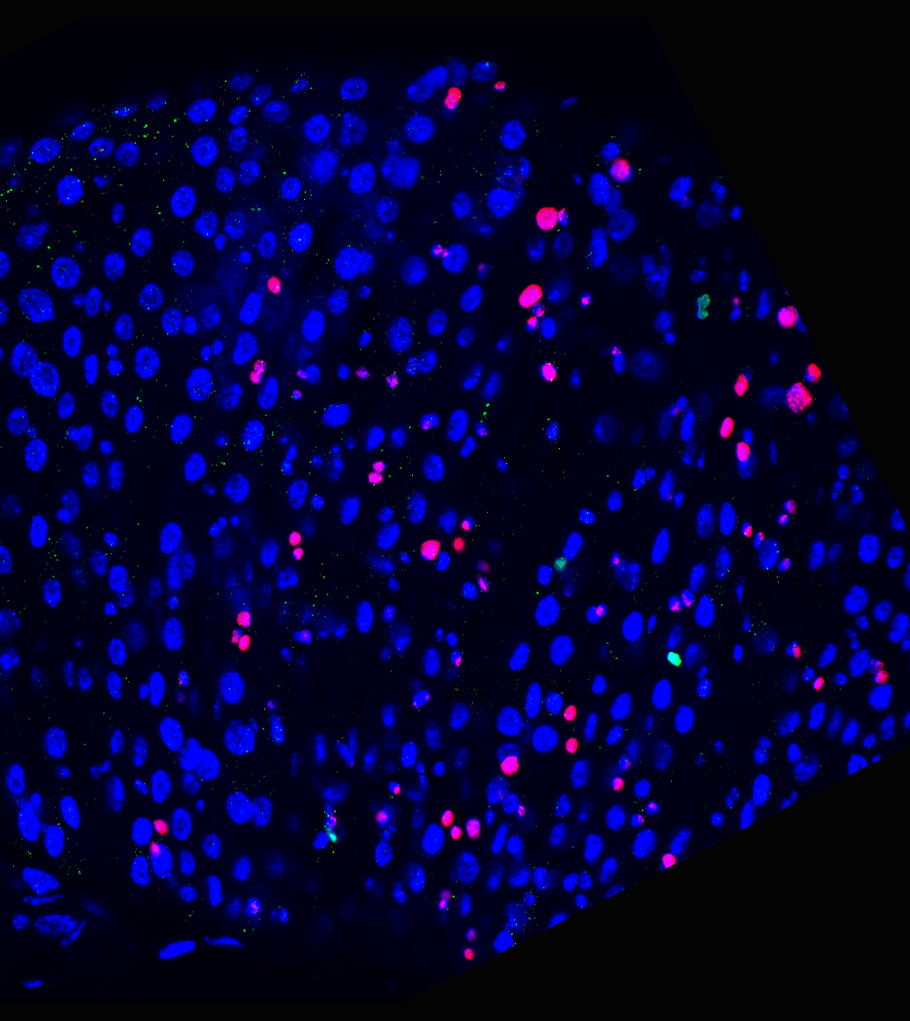

Supplement: Supplementary file 6 — Source data Fig. 3 [file 44319_2026_701_MOESM6_ESM.zip › Fig. 3/Fig 3B-B'/Dl-dwdr4RNAi_phh3+edu+DAPI.tif]

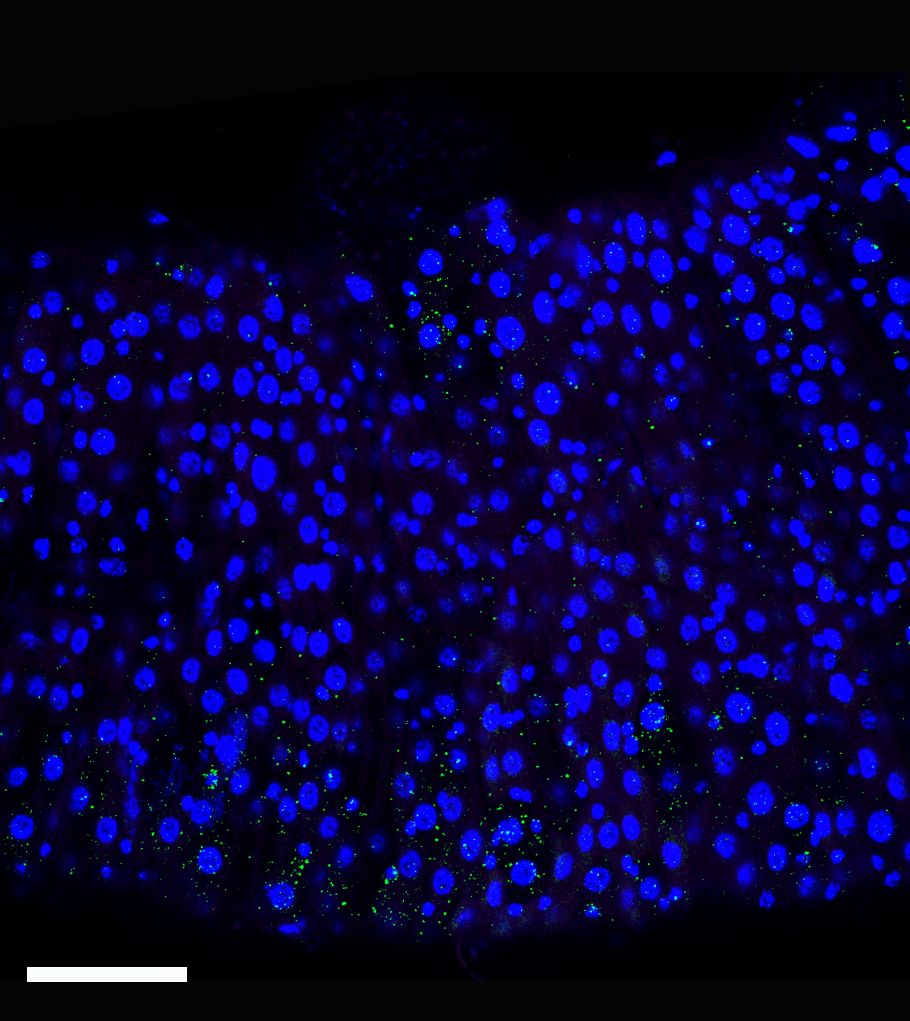

Supplement: Supplementary file 6 — Source data Fig. 3 [file 44319_2026_701_MOESM6_ESM.zip › Fig. 3/Fig 3B-B'/Dl-egfpRNAi_phh3+edu+DAPI.tif]

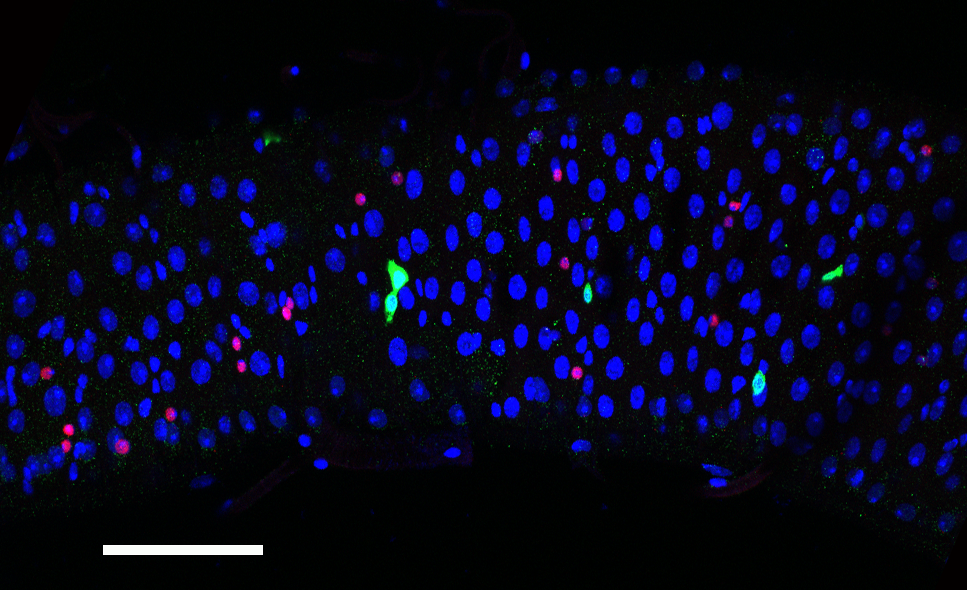

Supplement: Supplementary file 6 — Source data Fig. 3 [file 44319_2026_701_MOESM6_ESM.zip › Fig. 3/Fig. 3A'- A''/FRT19A_GFP+Pros+DAPI.tif]

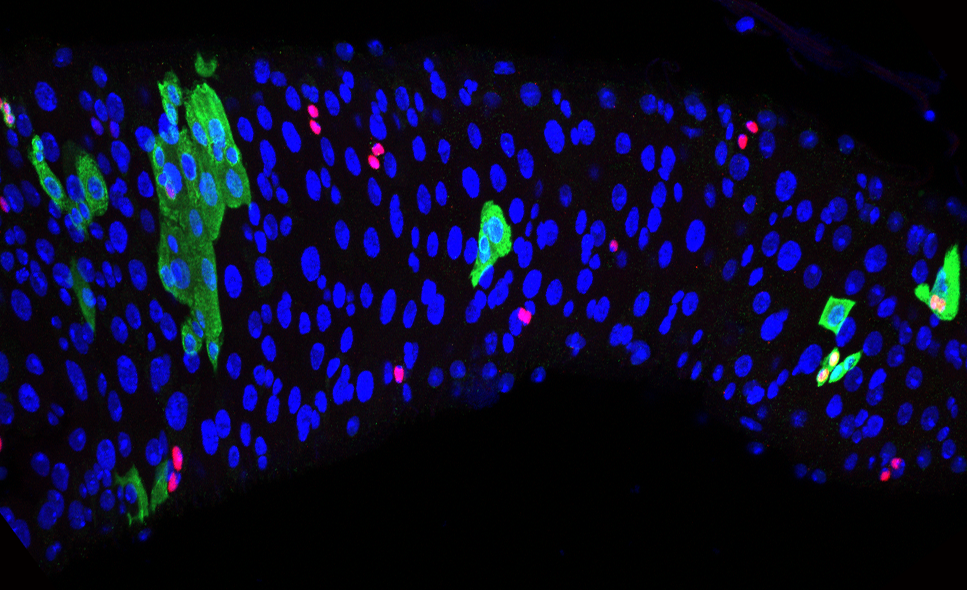

Supplement: Supplementary file 6 — Source data Fig. 3 [file 44319_2026_701_MOESM6_ESM.zip › Fig. 3/Fig. 3A'- A''/wh7FRT19A_GFP+Pros+DAPI.tif]

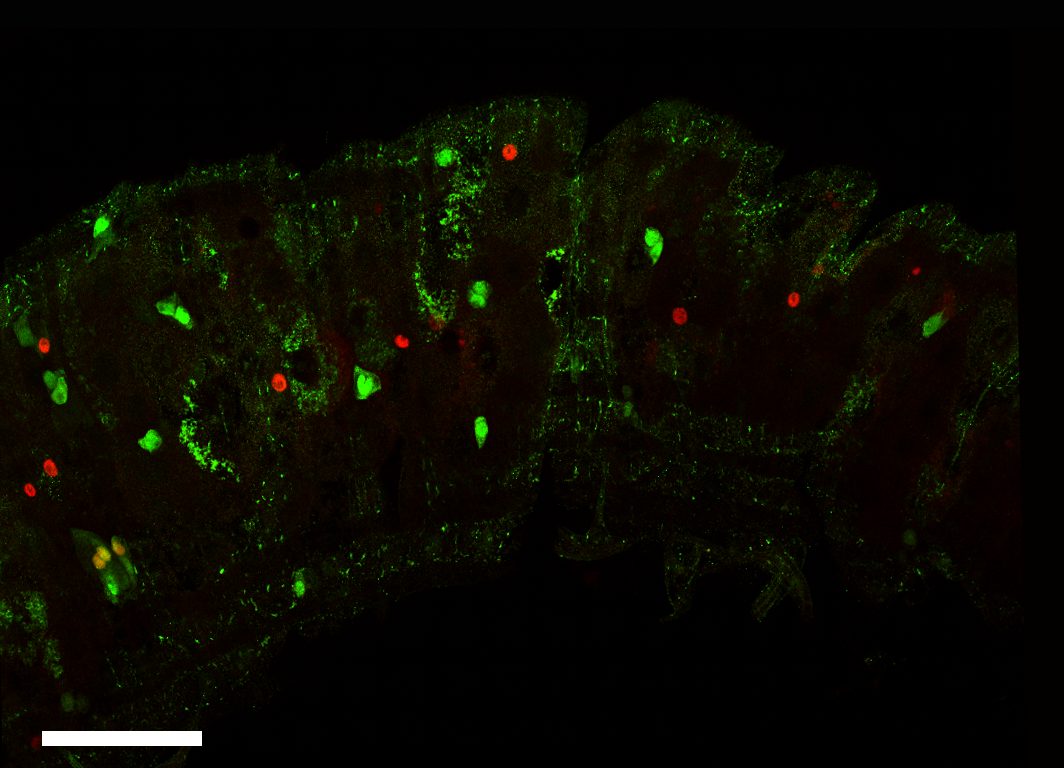

Supplement: Supplementary file 6 — Source data Fig. 3 [file 44319_2026_701_MOESM6_ESM.zip › Fig. 3/Fig. 3C-C'/Dl-mcherryRNAi_GFP+Pros.tif]

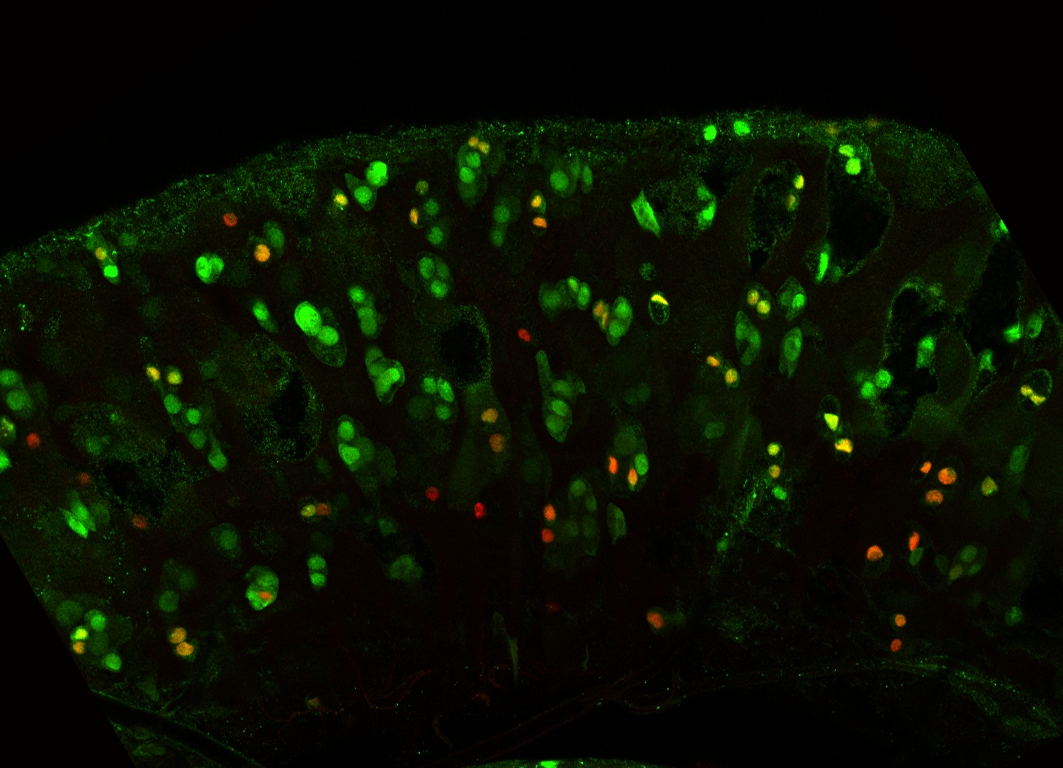

Supplement: Supplementary file 6 — Source data Fig. 3 [file 44319_2026_701_MOESM6_ESM.zip › Fig. 3/Fig. 3C-C'/Dl-UASp-dwdr4RNAi_GFP+Pros.tif]

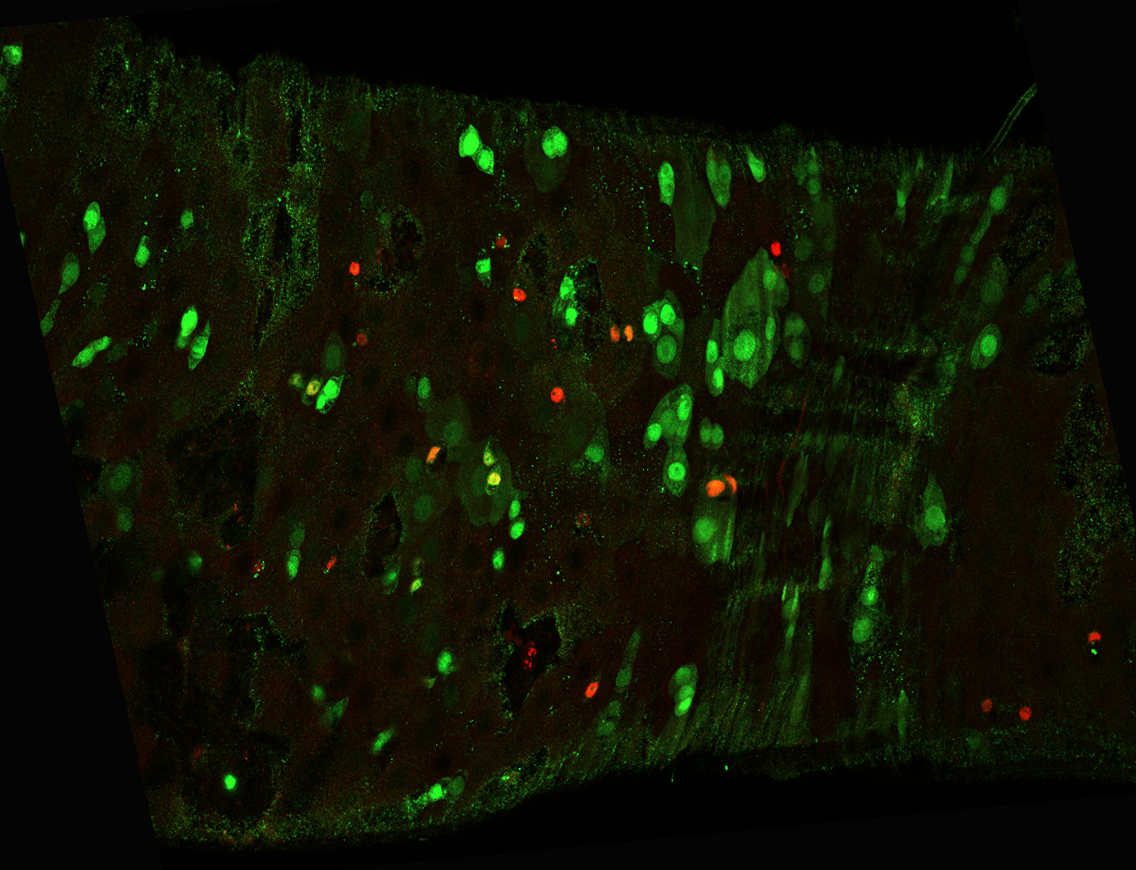

Supplement: Supplementary file 6 — Source data Fig. 3 [file 44319_2026_701_MOESM6_ESM.zip › Fig. 3/Fig. 3C-C'/Dl-UASt-dwdr4RNAi_GFP+Pros.tif]

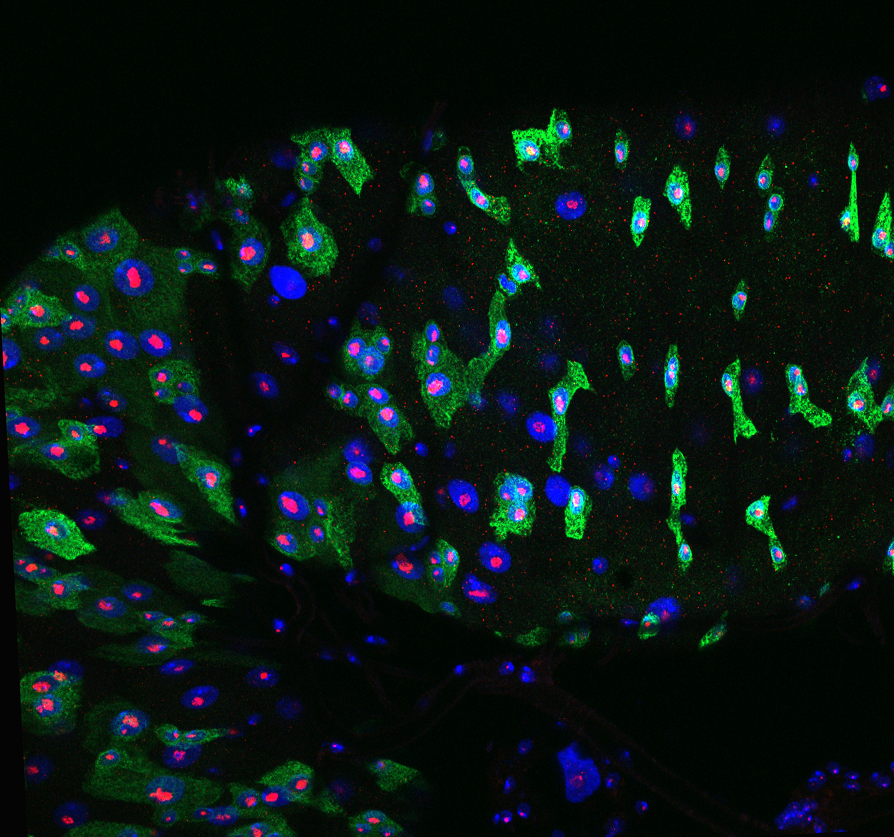

Supplement: Supplementary file 7 — Source data Fig. 4 [file 44319_2026_701_MOESM7_ESM.zip › Fig. 4/Fig. 4B-B'/wh7_GFP+Fibrillarin+DAPI.tif]

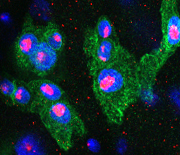

Supplement: Supplementary file 7 — Source data Fig. 4 [file 44319_2026_701_MOESM7_ESM.zip › Fig. 4/Fig. 4B-B'/wh7_GFP+Fibrillarin+DAPI_cut.tif]

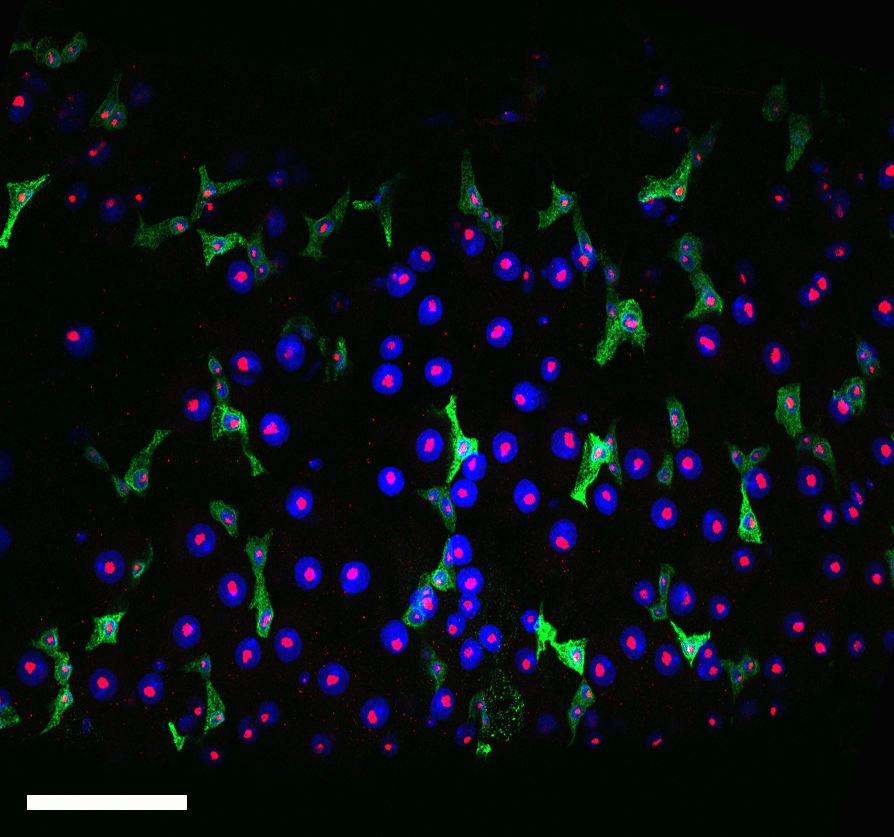

Supplement: Supplementary file 7 — Source data Fig. 4 [file 44319_2026_701_MOESM7_ESM.zip › Fig. 4/Fig. 4B-B'/WT_GFP+Fibrillarin+DAPI.tif]

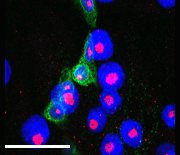

Supplement: Supplementary file 7 — Source data Fig. 4 [file 44319_2026_701_MOESM7_ESM.zip › Fig. 4/Fig. 4B-B'/WT_GFP+Fibrillarin+DAPI_cut.tif]

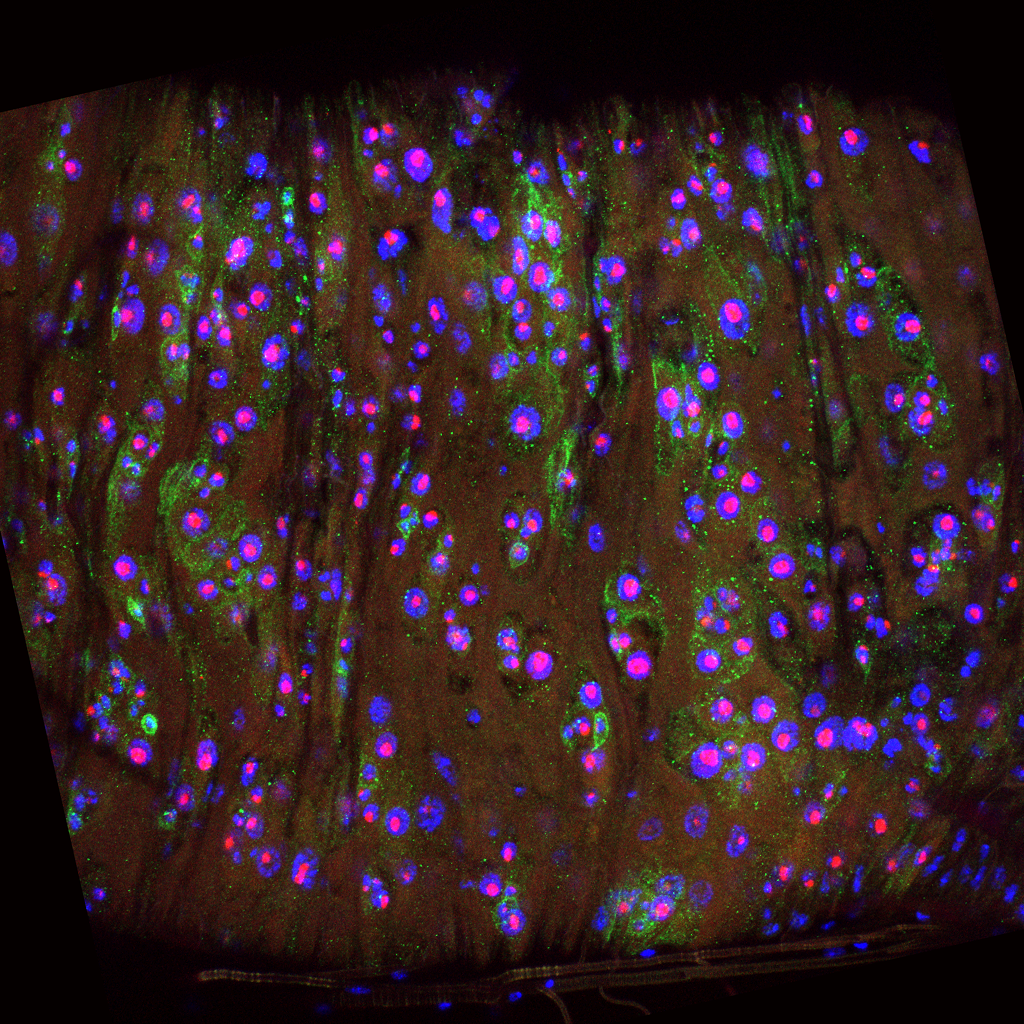

Supplement: Supplementary file 7 — Source data Fig. 4 [file 44319_2026_701_MOESM7_ESM.zip › Fig. 4/Fig. 4C-C'/wh7_Dl+EU+DAPI.tif]

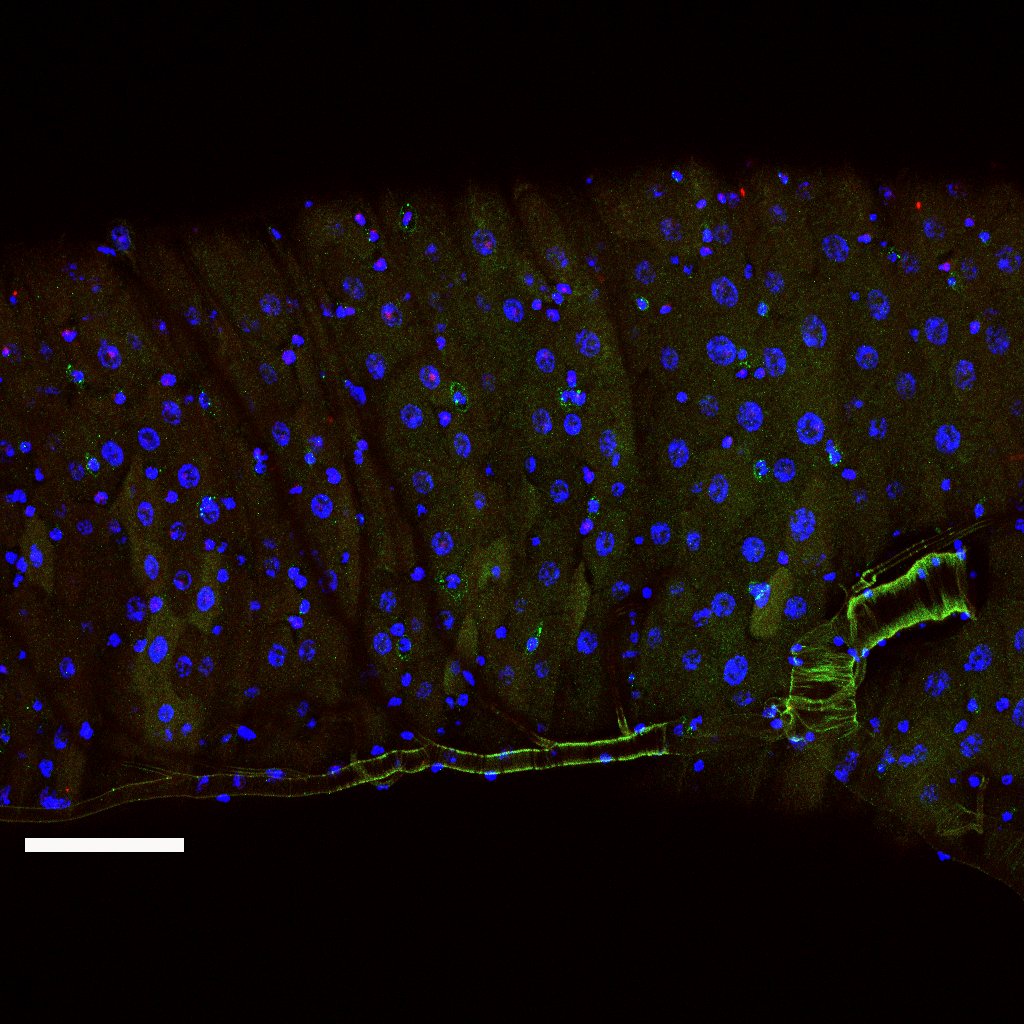

Supplement: Supplementary file 7 — Source data Fig. 4 [file 44319_2026_701_MOESM7_ESM.zip › Fig. 4/Fig. 4C-C'/WT_Dl+EU+DAPI.tif]

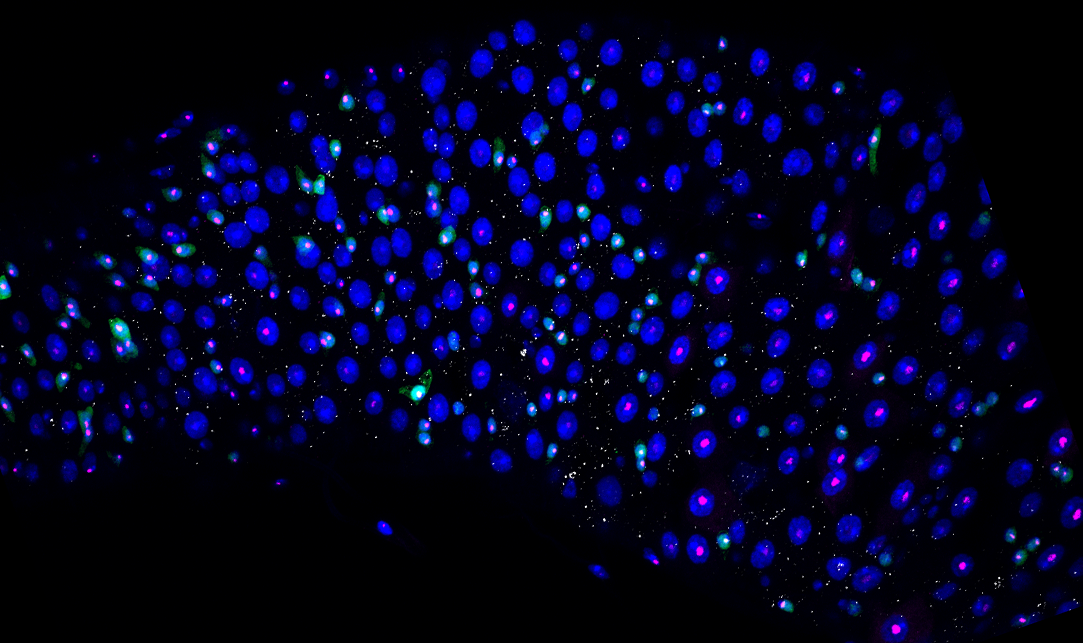

Supplement: Supplementary file 7 — Source data Fig. 4 [file 44319_2026_701_MOESM7_ESM.zip › Fig. 4/Fig. 4D-D'/esgts-dwdr4RNAi_GFP+phh3+Fib+DAPI_BMH-21.tif]

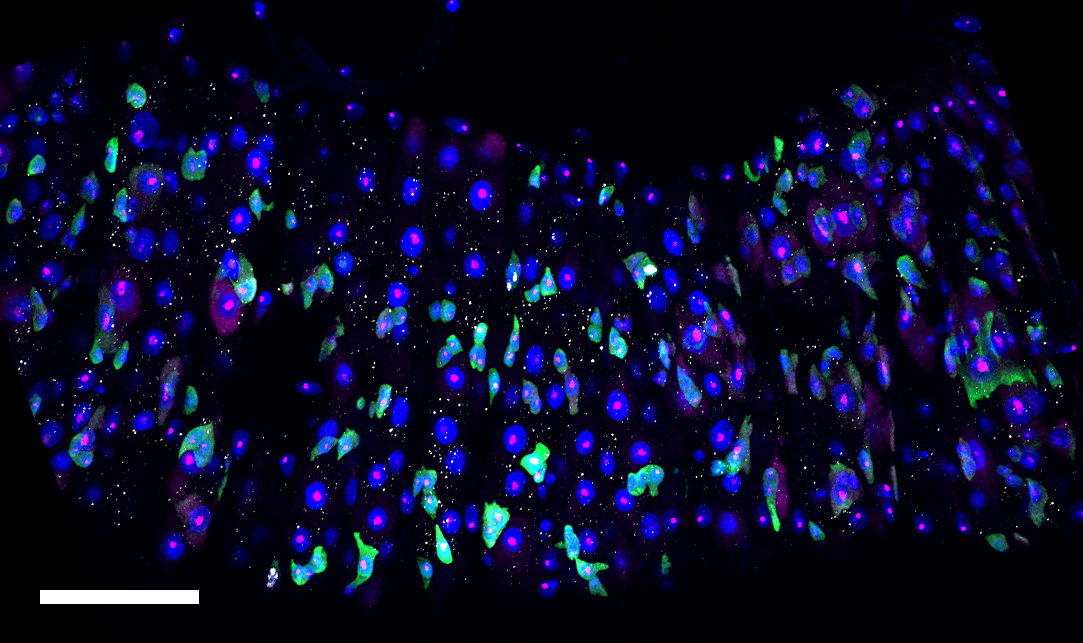

Supplement: Supplementary file 7 — Source data Fig. 4 [file 44319_2026_701_MOESM7_ESM.zip › Fig. 4/Fig. 4D-D'/esgts-dwdr4RNAi_GFP+phh3+Fib+DAPI_DMSO.tif]

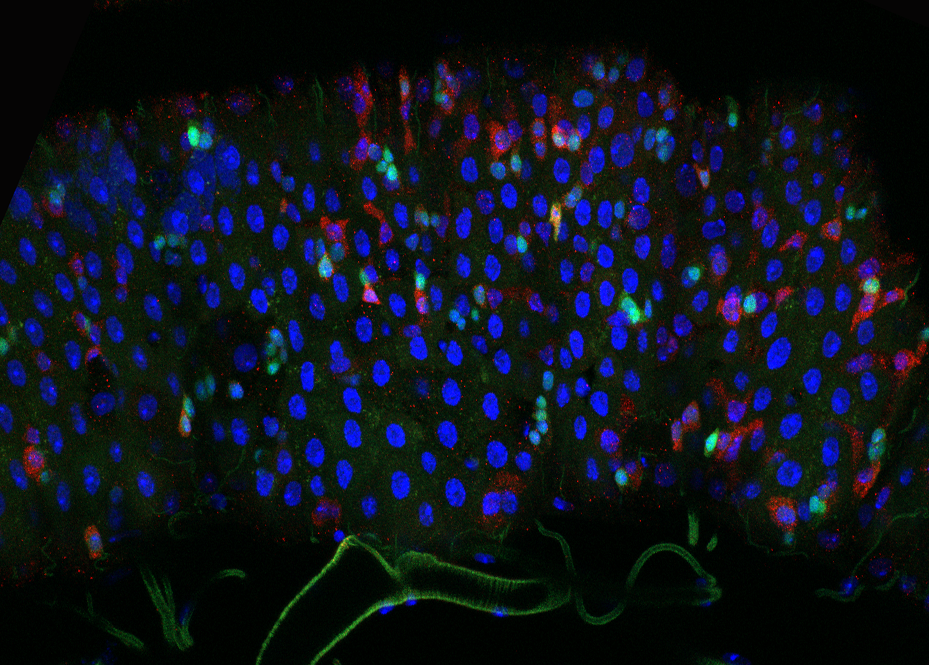

Supplement: Supplementary file 8 — Source data Fig. 5 [file 44319_2026_701_MOESM8_ESM.zip › Fig. 5/Fig. 5A-A'/Dlts-dwdr4RNAi_GFP+p4E-BP+DAPI.tif]

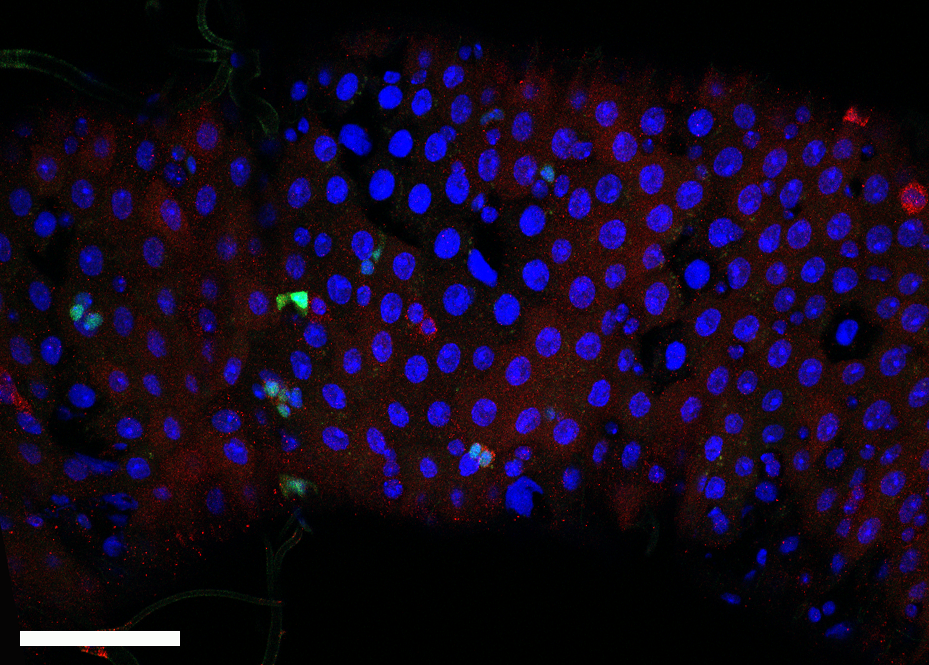

Supplement: Supplementary file 8 — Source data Fig. 5 [file 44319_2026_701_MOESM8_ESM.zip › Fig. 5/Fig. 5A-A'/Dlts-mcherryRNAi_GFP+p4E-BP+DAPI.tif]

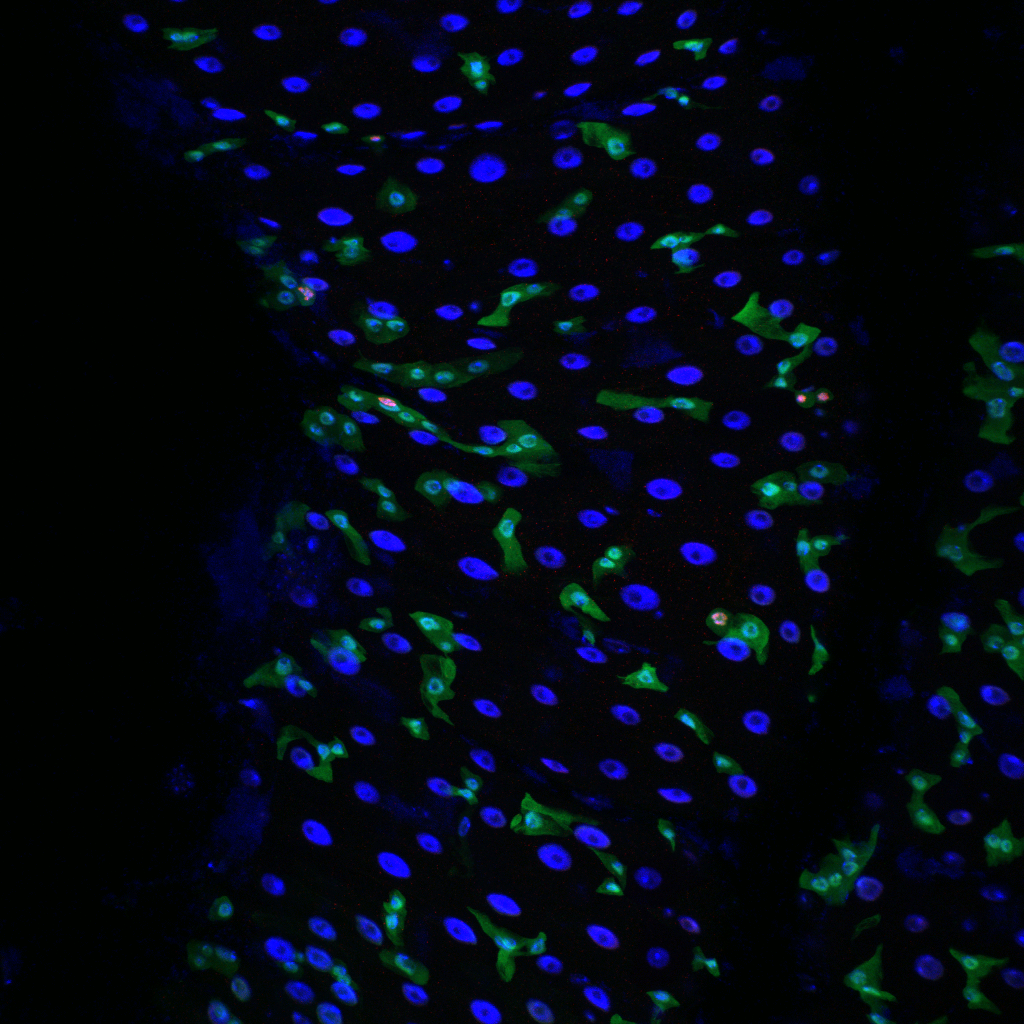

Supplement: Supplementary file 8 — Source data Fig. 5 [file 44319_2026_701_MOESM8_ESM.zip › Fig. 5/Fig. 5B-B'/esgts-dwdr4RNAi_GFP+pHH3+DAPI.tif]

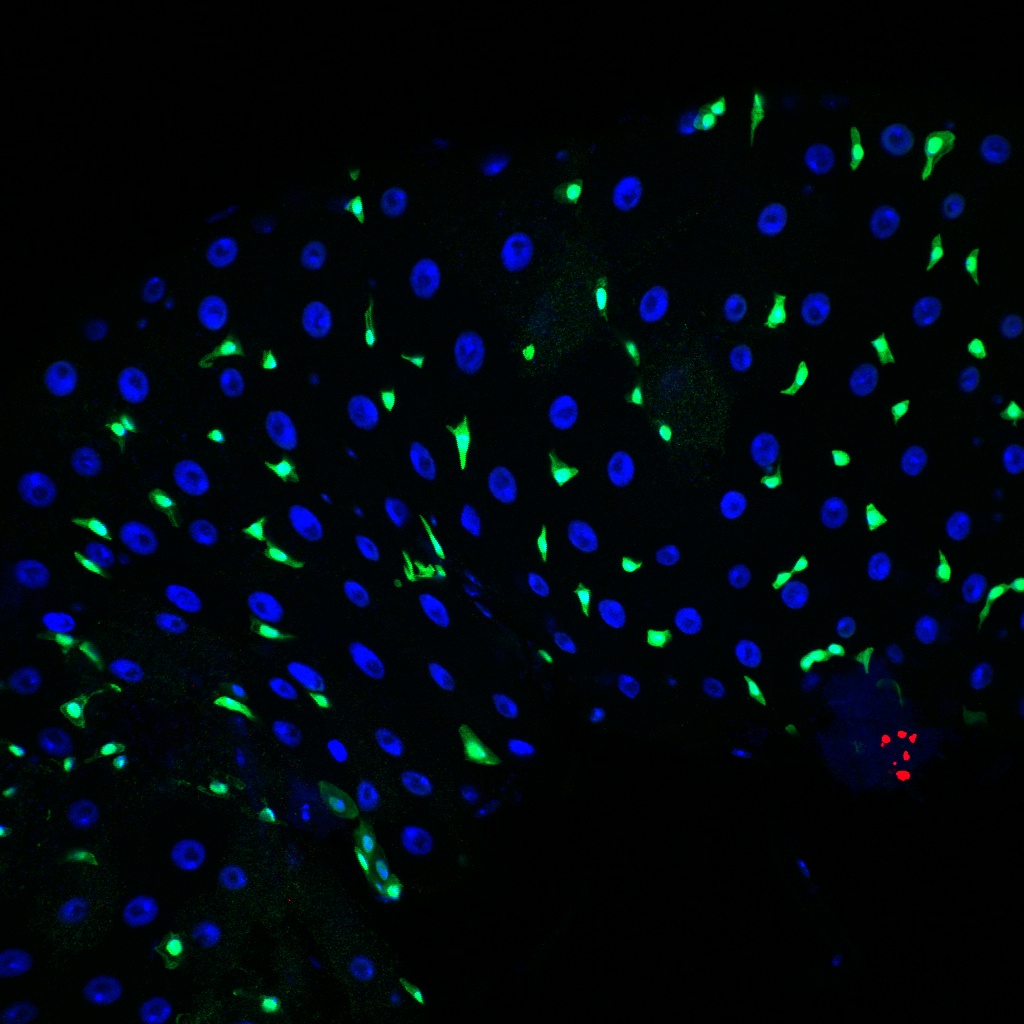

Supplement: Supplementary file 8 — Source data Fig. 5 [file 44319_2026_701_MOESM8_ESM.zip › Fig. 5/Fig. 5B-B'/ests-dwdr4RNAi;TORRNAi_GFP+pHH3+DAPI.jpg]

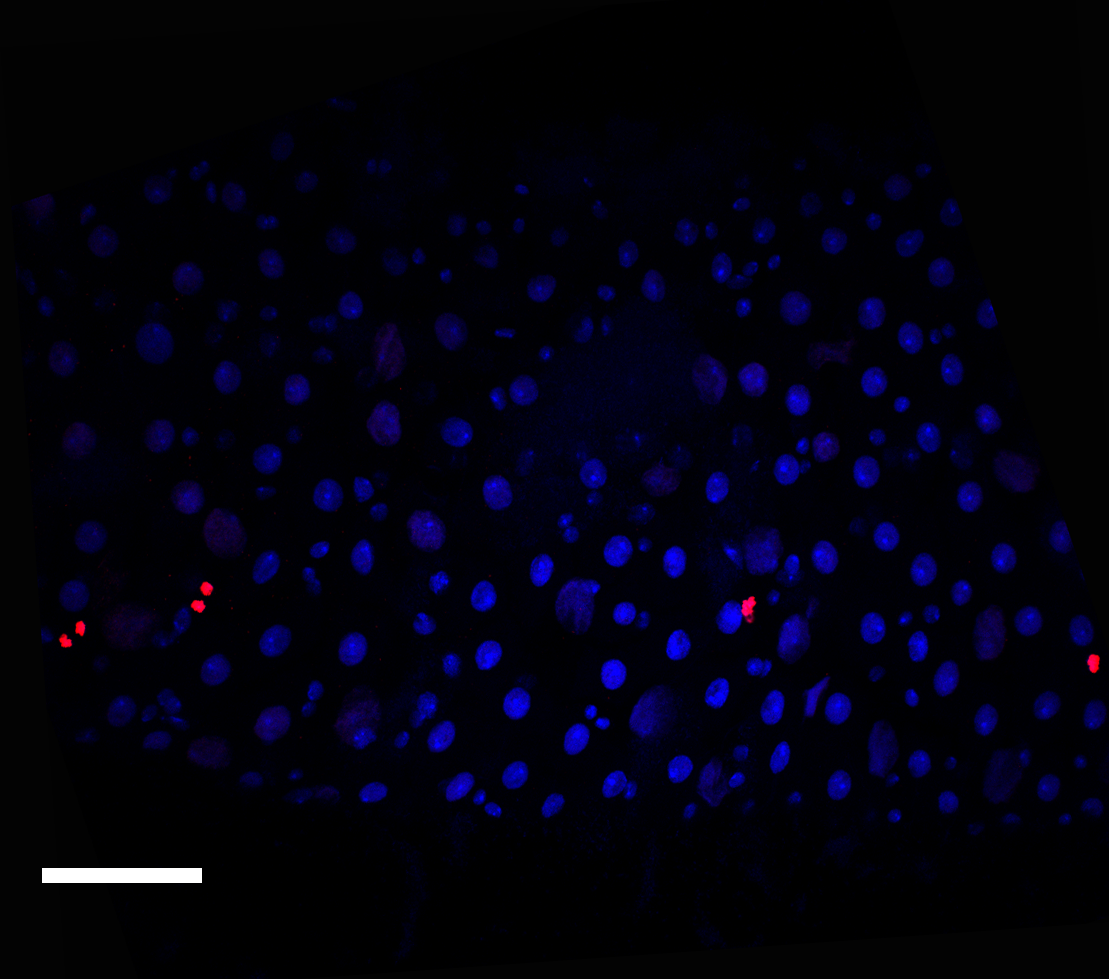

Supplement: Supplementary file 8 — Source data Fig. 5 [file 44319_2026_701_MOESM8_ESM.zip › Fig. 5/Fig. 5C-C'/wh7_phh3+DAPI_dH2O.tif]

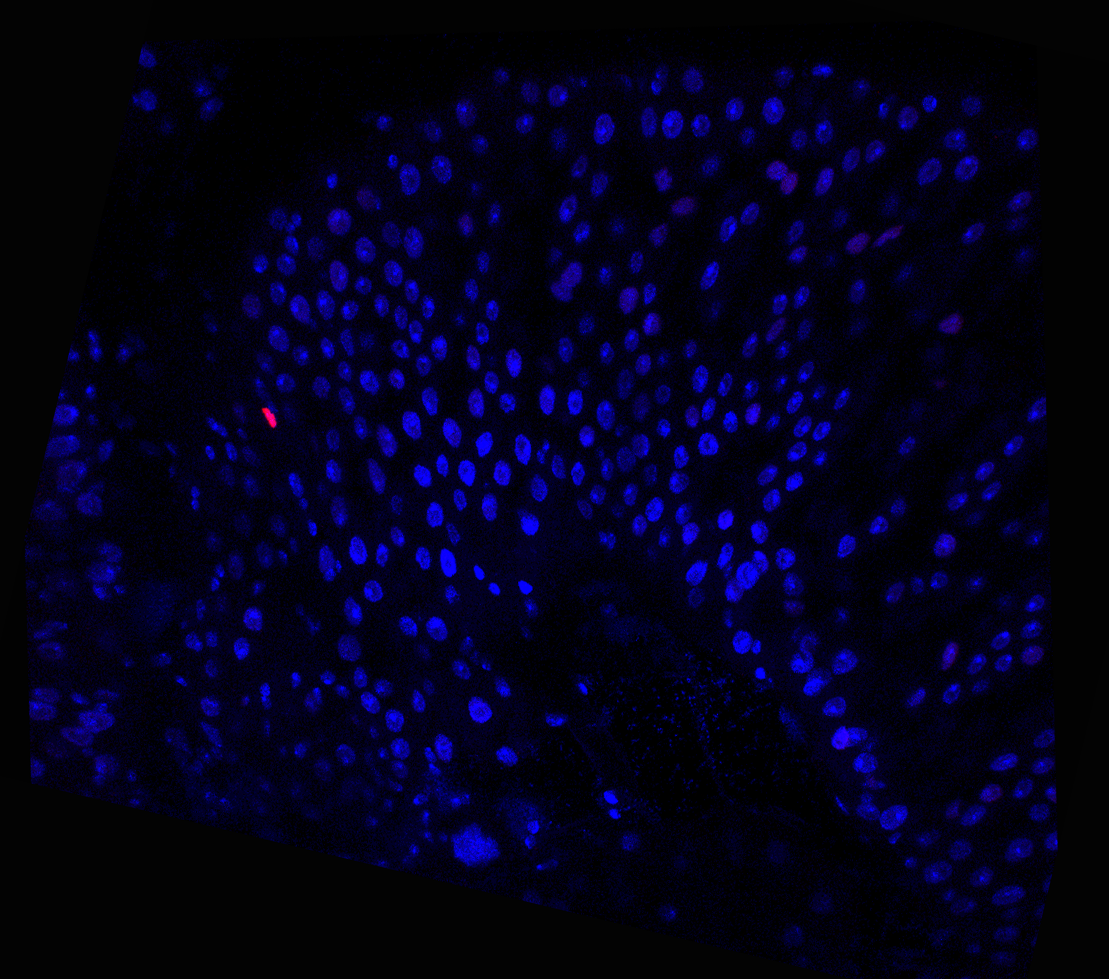

Supplement: Supplementary file 8 — Source data Fig. 5 [file 44319_2026_701_MOESM8_ESM.zip › Fig. 5/Fig. 5C-C'/wh7_phh3+DAPI_Rapamycin.tif]

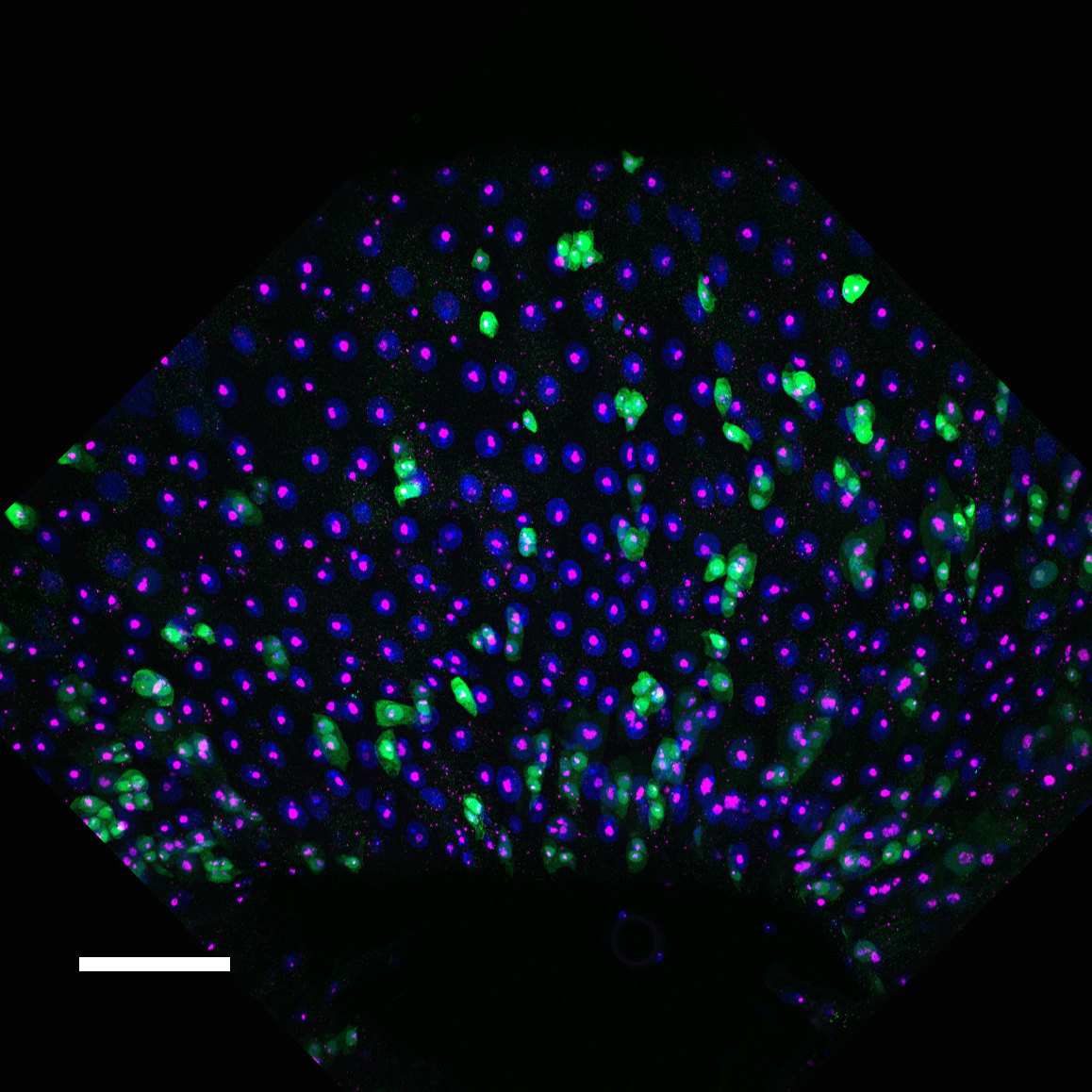

Supplement: Supplementary file 8 — Source data Fig. 5 [file 44319_2026_701_MOESM8_ESM.zip › Fig. 5/Fig. 5D-D'/esgts-dwdr4RNAi_GFP+Fibrillarin+DAPI_dH20.tif]

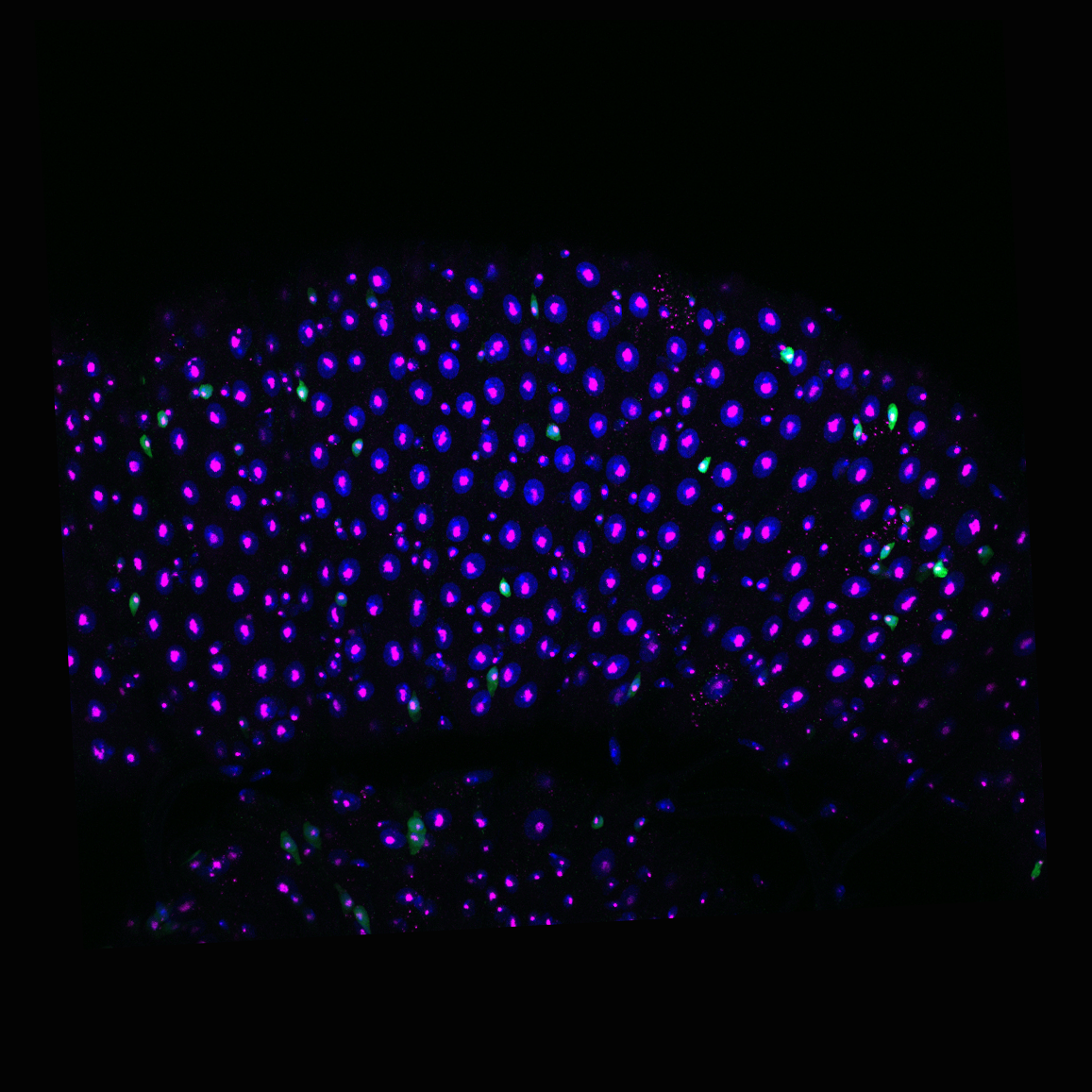

Supplement: Supplementary file 8 — Source data Fig. 5 [file 44319_2026_701_MOESM8_ESM.zip › Fig. 5/Fig. 5D-D'/esgts-dwdr4RNAi_GFP+Fibrillarin+DAPI_Rapamycin.tif]

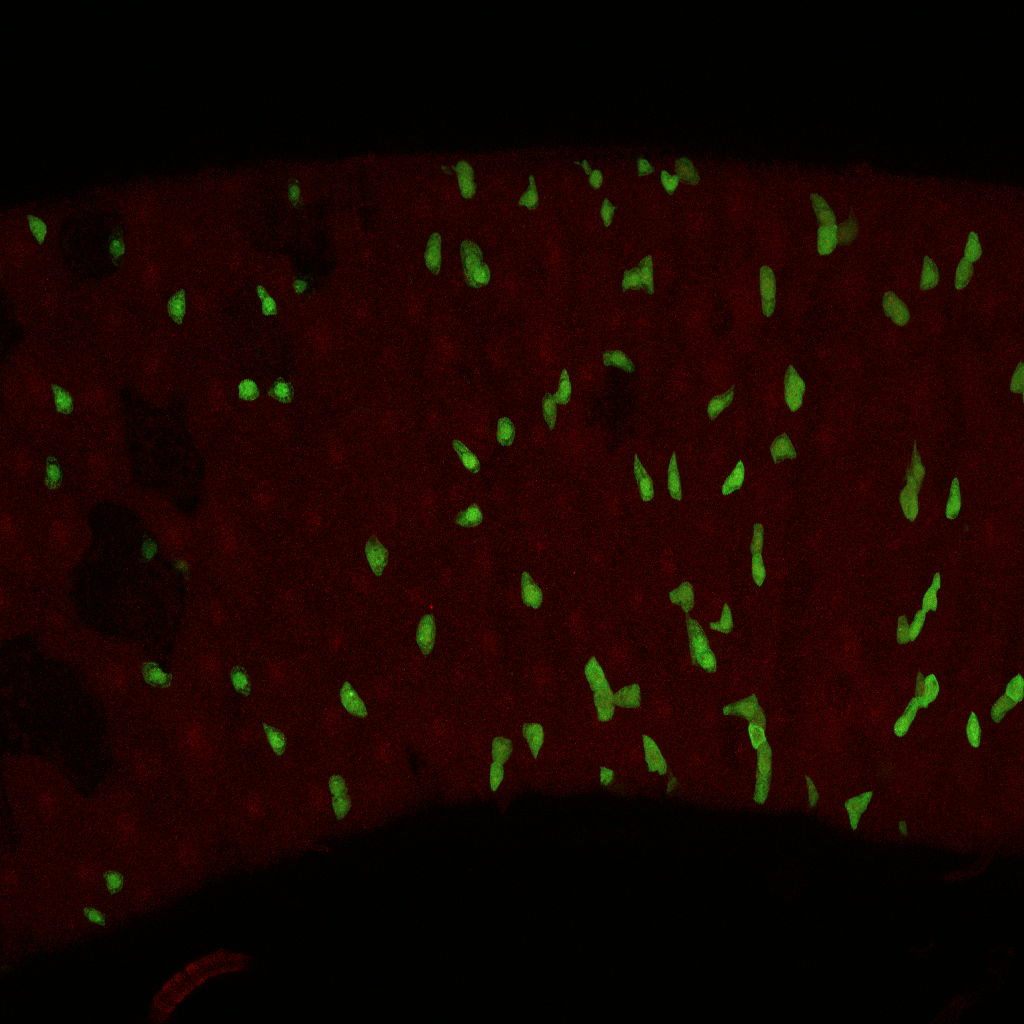

Supplement: Supplementary file 8 — Source data Fig. 5 [file 44319_2026_701_MOESM8_ESM.zip › Fig. 5/Fig. 5E-E'/esgts-mcherryRNAi_GFP+dmyc.tif]

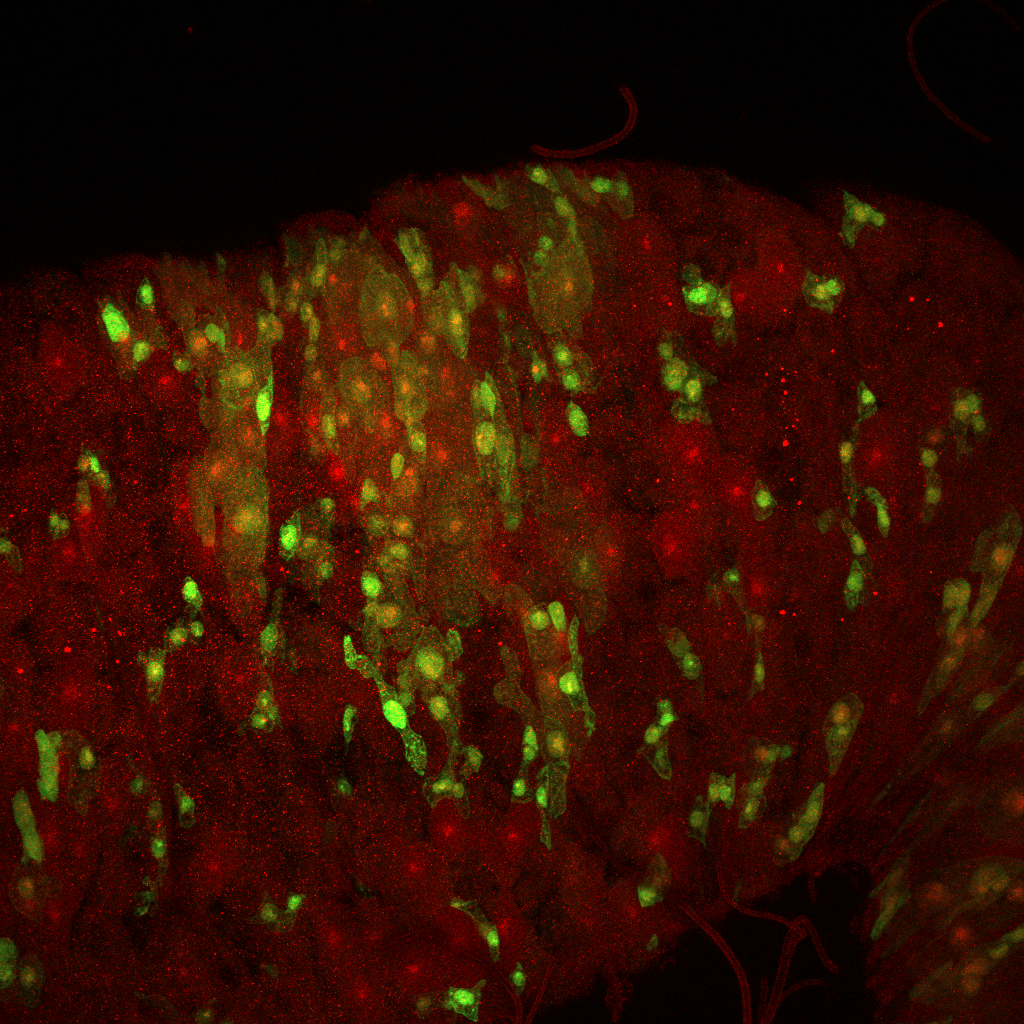

Supplement: Supplementary file 8 — Source data Fig. 5 [file 44319_2026_701_MOESM8_ESM.zip › Fig. 5/Fig. 5E-E'/esgts_dwdr4RNAi_GFP+myc.tif]

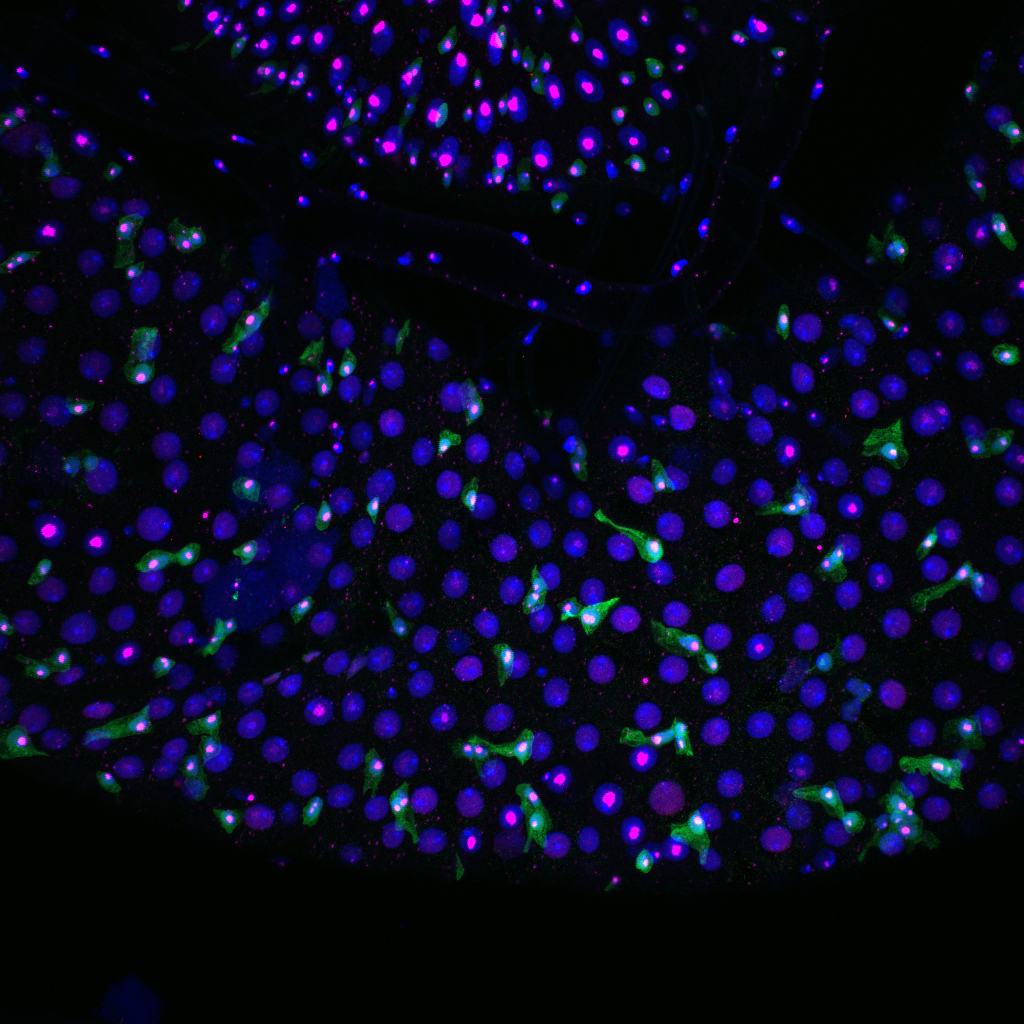

Supplement: Supplementary file 8 — Source data Fig. 5 [file 44319_2026_701_MOESM8_ESM.zip › Fig. 5/Fig. 5F-F'/esgts-dmyc4;dwdr4RNAi_GFP+phh3+fib+DAPI.tif]

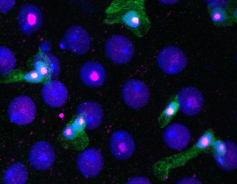

Supplement: Supplementary file 8 — Source data Fig. 5 [file 44319_2026_701_MOESM8_ESM.zip › Fig. 5/Fig. 5F-F'/esgts-dmyc4;dwdr4RNai_GFP+phh3+fib+DAPI_cut.tif]

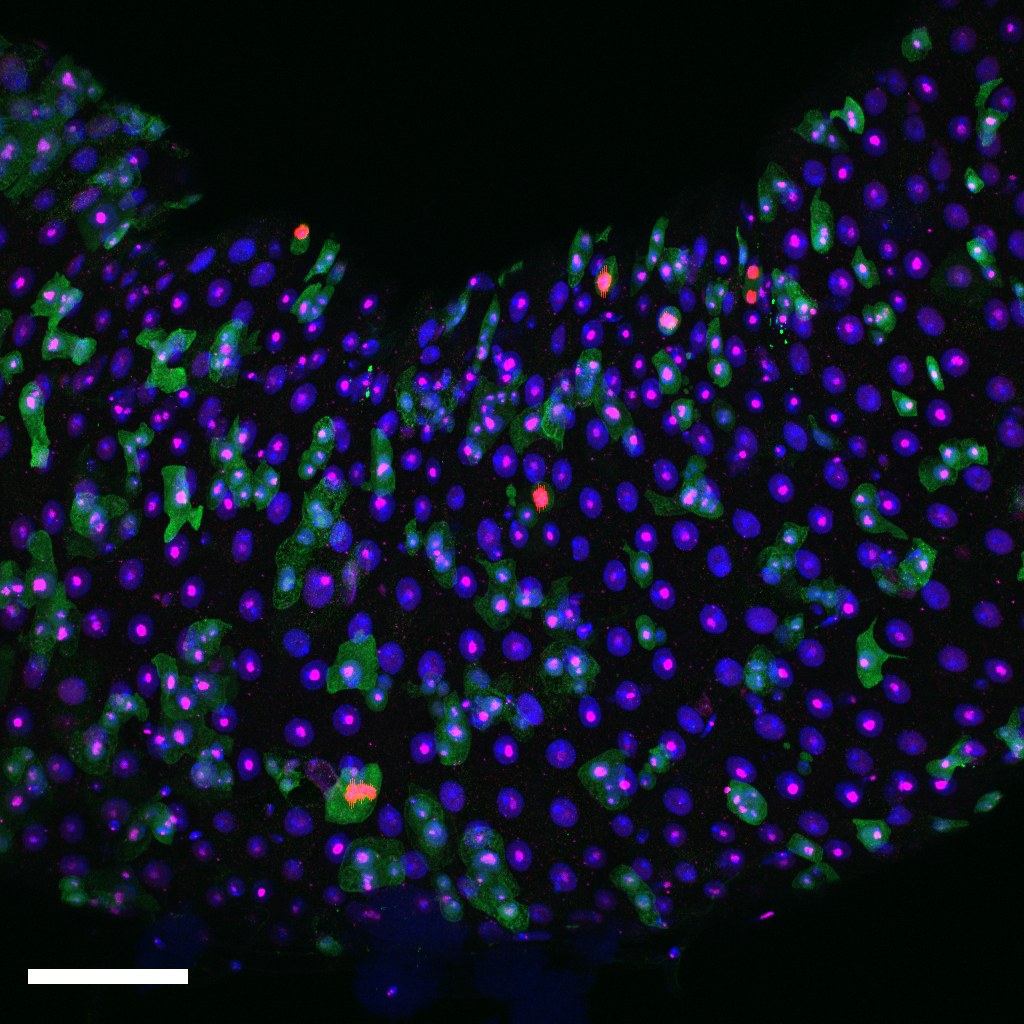

Supplement: Supplementary file 8 — Source data Fig. 5 [file 44319_2026_701_MOESM8_ESM.zip › Fig. 5/Fig. 5F-F'/esgts-dwdr4RNAi_GFP+phh3+DAPI.tif]

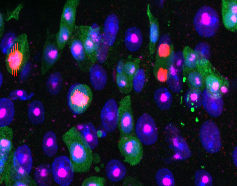

Supplement: Supplementary file 8 — Source data Fig. 5 [file 44319_2026_701_MOESM8_ESM.zip › Fig. 5/Fig. 5F-F'/esgts-dwdr4RNAi_GFP+phh3+DAPI_cut.tif]

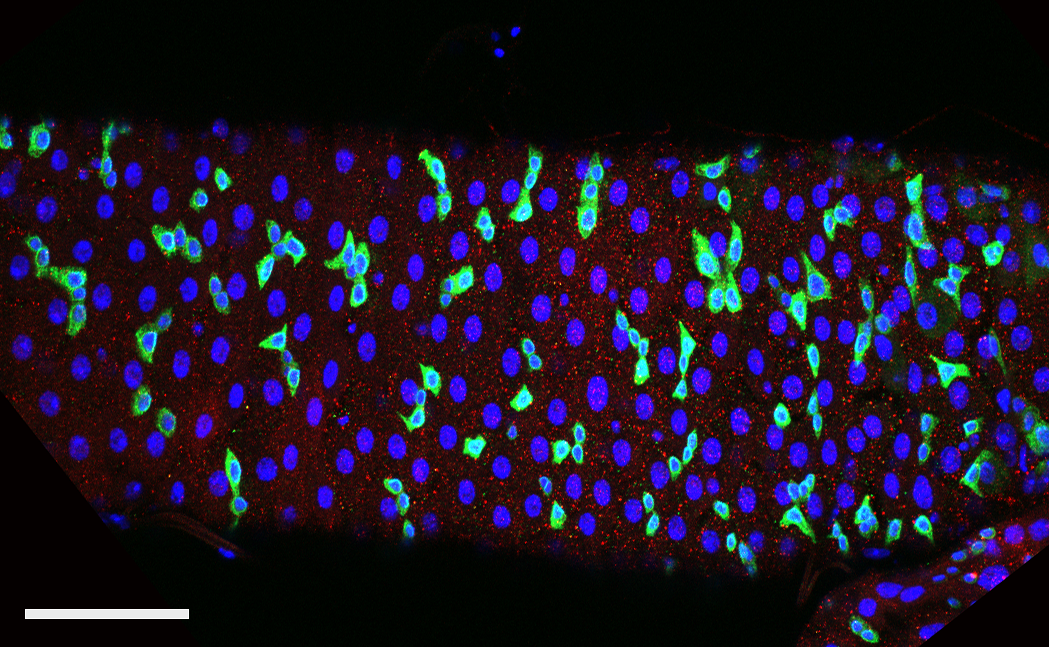

Supplement: Supplementary file 9 — Source data Fig. 6 [file 44319_2026_701_MOESM9_ESM.zip › Fig. 6/Fig. 6A-A'/esg-mcd8gfp_GFP+pJNK+DAPI.tif]

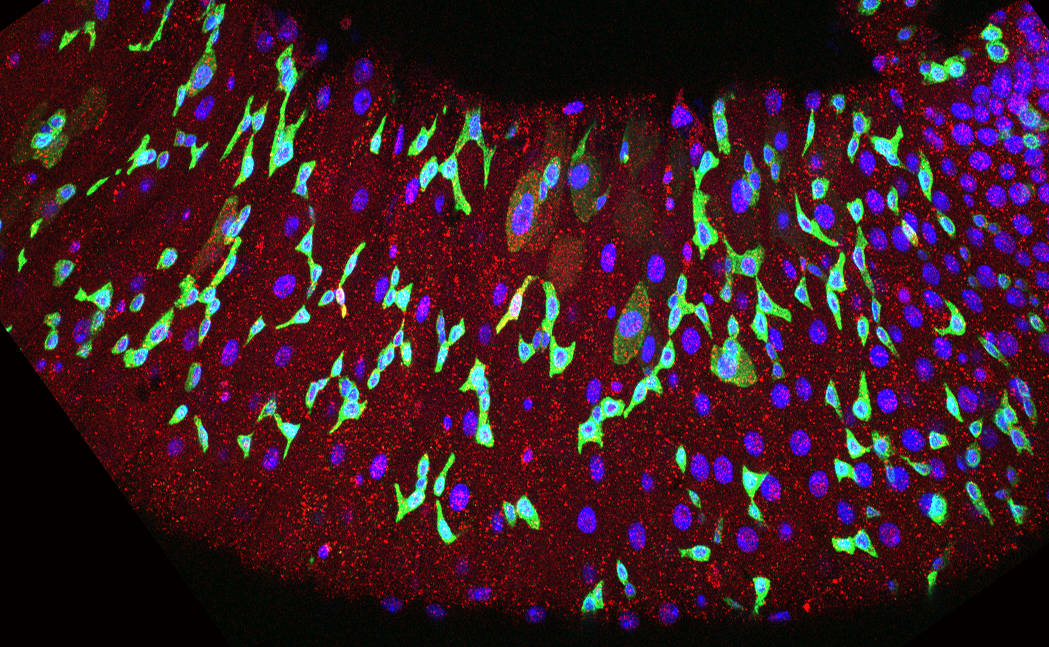

Supplement: Supplementary file 9 — Source data Fig. 6 [file 44319_2026_701_MOESM9_ESM.zip › Fig. 6/Fig. 6A-A'/wh7;mcd8gfp_GFP+pJNK+DAPI.tif]

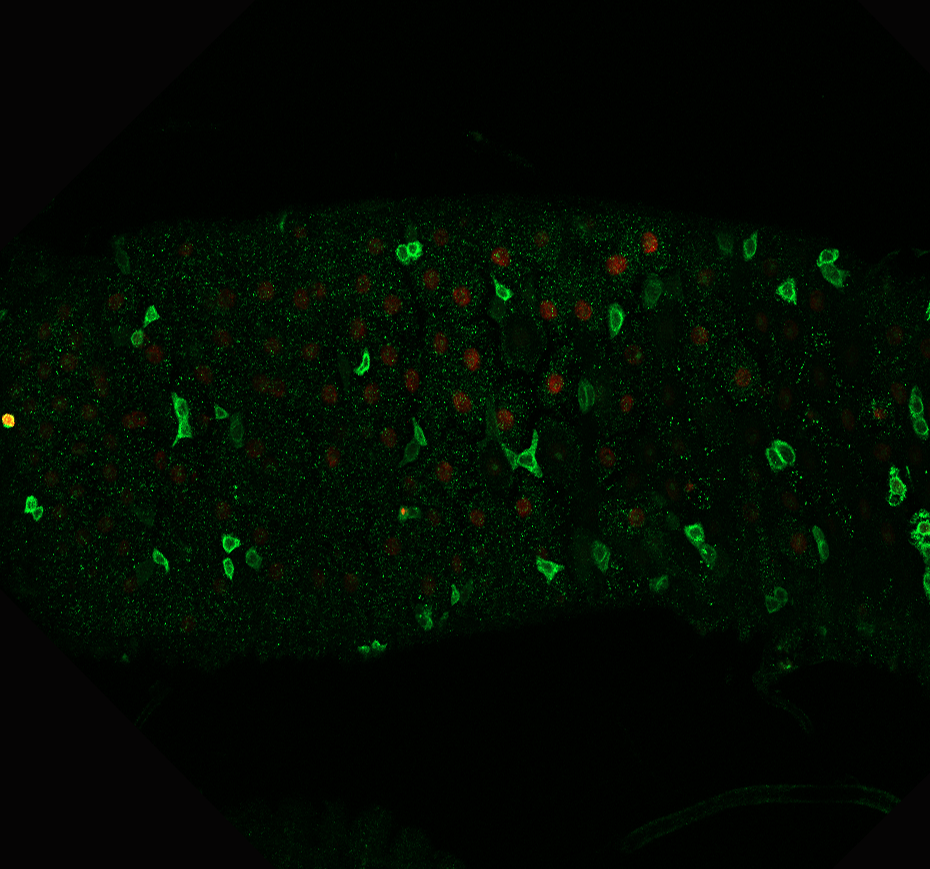

Supplement: Supplementary file 9 — Source data Fig. 6 [file 44319_2026_701_MOESM9_ESM.zip › Fig. 6/Fig. 6B-B'/wh7;hepRNAi_GFP+phh3.tif]

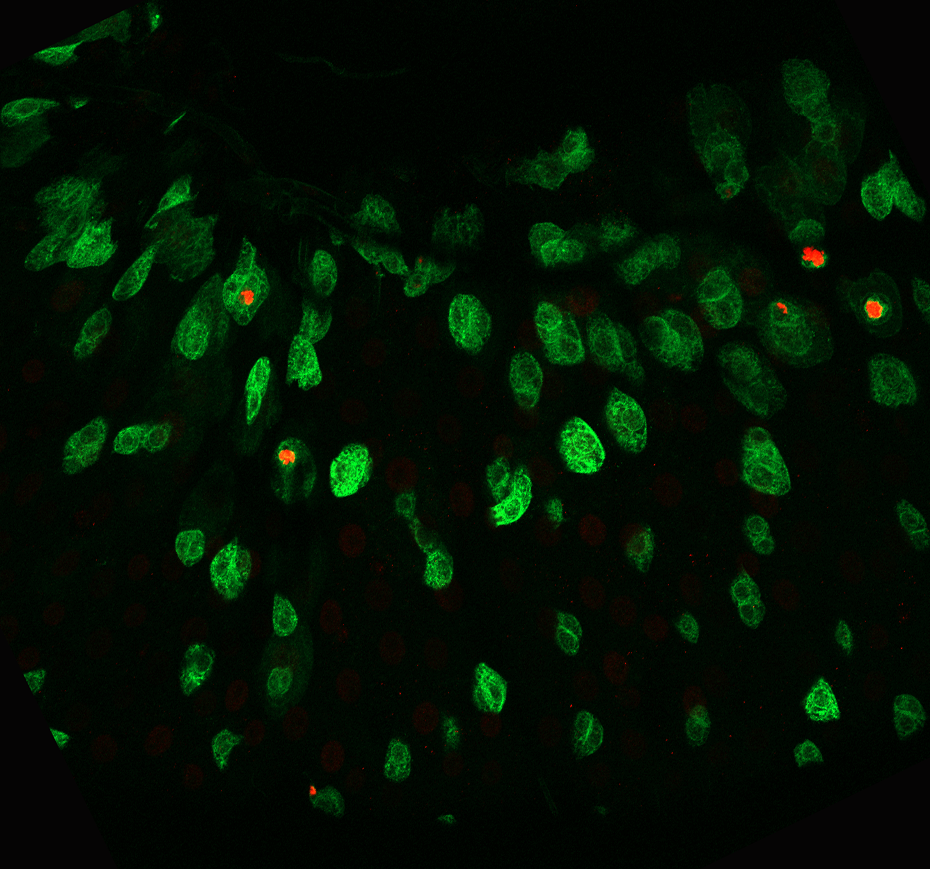

Supplement: Supplementary file 9 — Source data Fig. 6 [file 44319_2026_701_MOESM9_ESM.zip › Fig. 6/Fig. 6B-B'/wh7_GFP+phh3.tif]

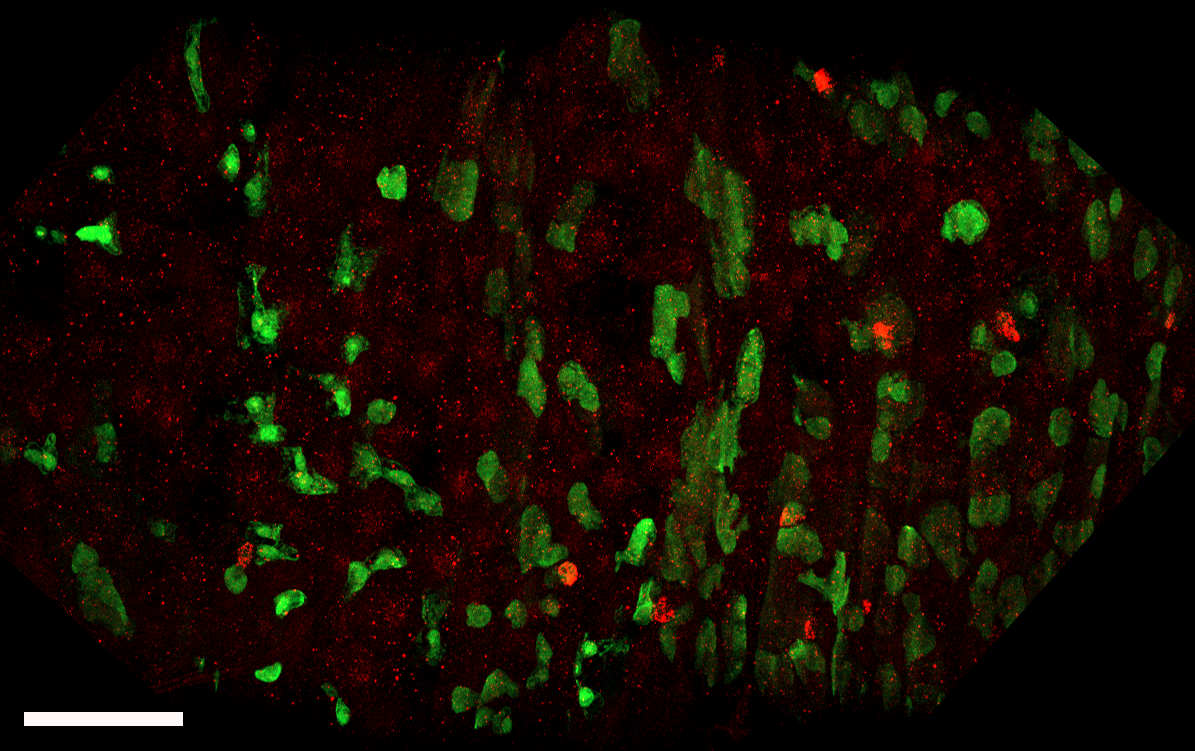

Supplement: Supplementary file 9 — Source data Fig. 6 [file 44319_2026_701_MOESM9_ESM.zip › Fig. 6/Fig. 6C-C'/esgts-dwdr4RNAi_GFP+pJNK_dH2O.tif]

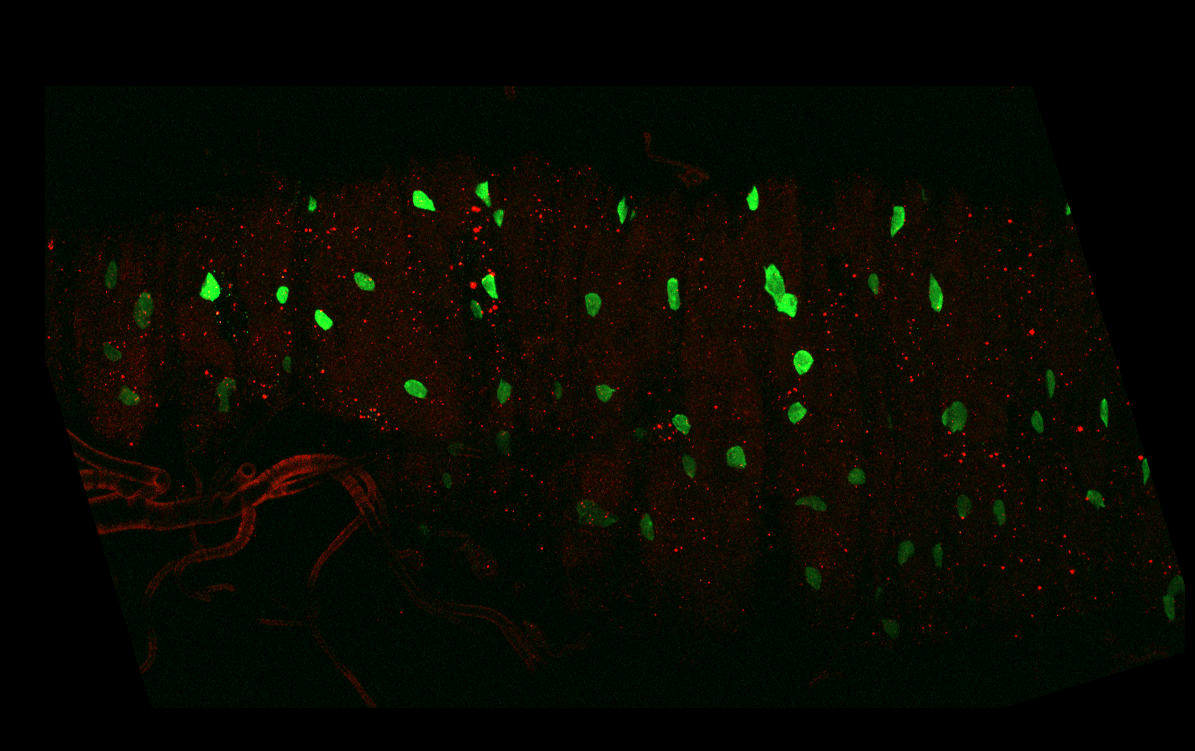

Supplement: Supplementary file 9 — Source data Fig. 6 [file 44319_2026_701_MOESM9_ESM.zip › Fig. 6/Fig. 6C-C'/esgts-dwdr4RNAi_GFP+pJNK_Rapamycin.tif]

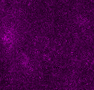

Supplement: Supplementary file 9 — Source data Fig. 6 [file 44319_2026_701_MOESM9_ESM.zip › Fig. 6/Fig. 6D-D'/wh7;hepRNAi_dmyc_cut.tif]

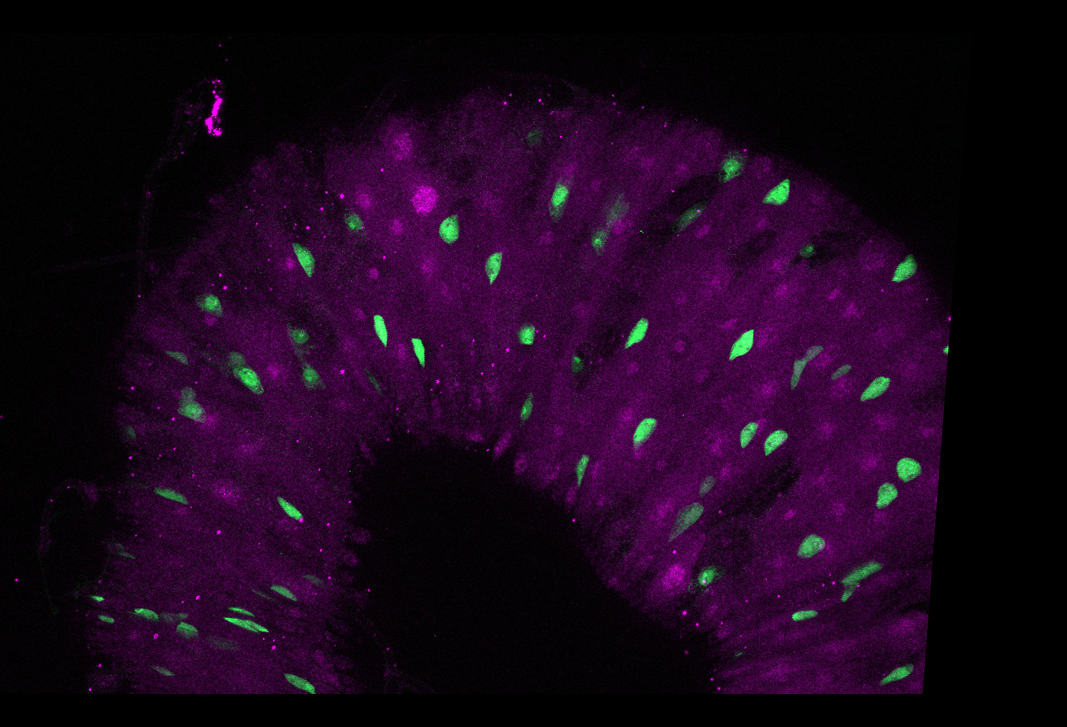

Supplement: Supplementary file 9 — Source data Fig. 6 [file 44319_2026_701_MOESM9_ESM.zip › Fig. 6/Fig. 6D-D'/wh7;hepRNAi_GFP+dmyc.tif]

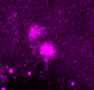

Supplement: Supplementary file 9 — Source data Fig. 6 [file 44319_2026_701_MOESM9_ESM.zip › Fig. 6/Fig. 6D-D'/wh7_dmyc.tif]

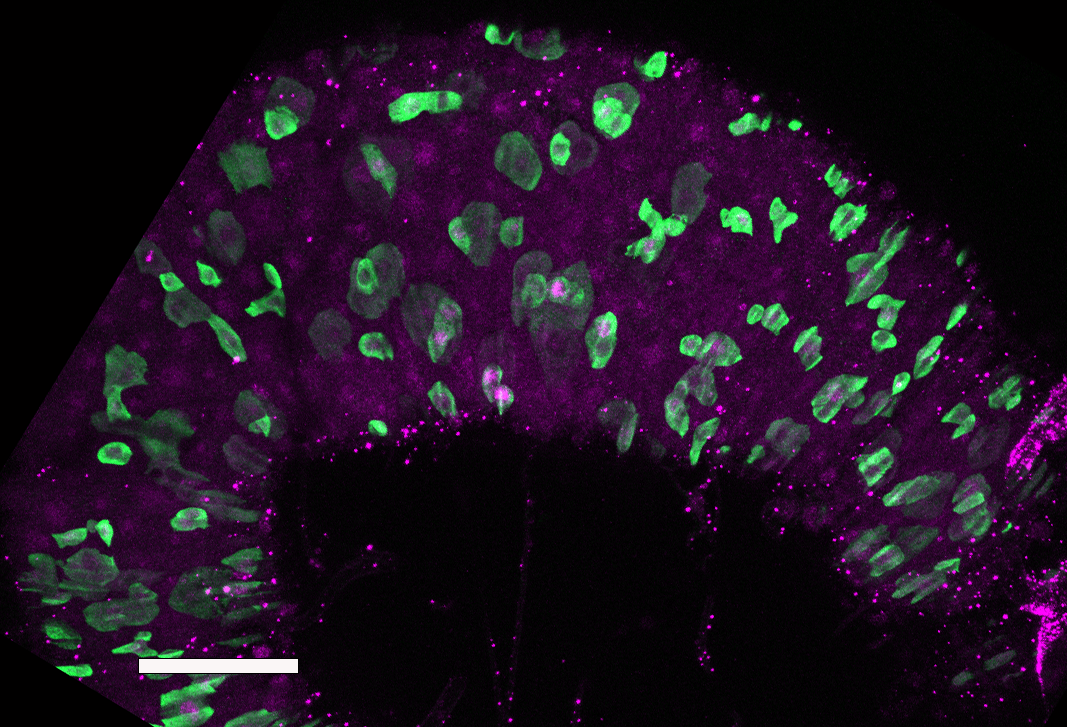

Supplement: Supplementary file 9 — Source data Fig. 6 [file 44319_2026_701_MOESM9_ESM.zip › Fig. 6/Fig. 6D-D'/wh7_GFP+dmyc.tif]

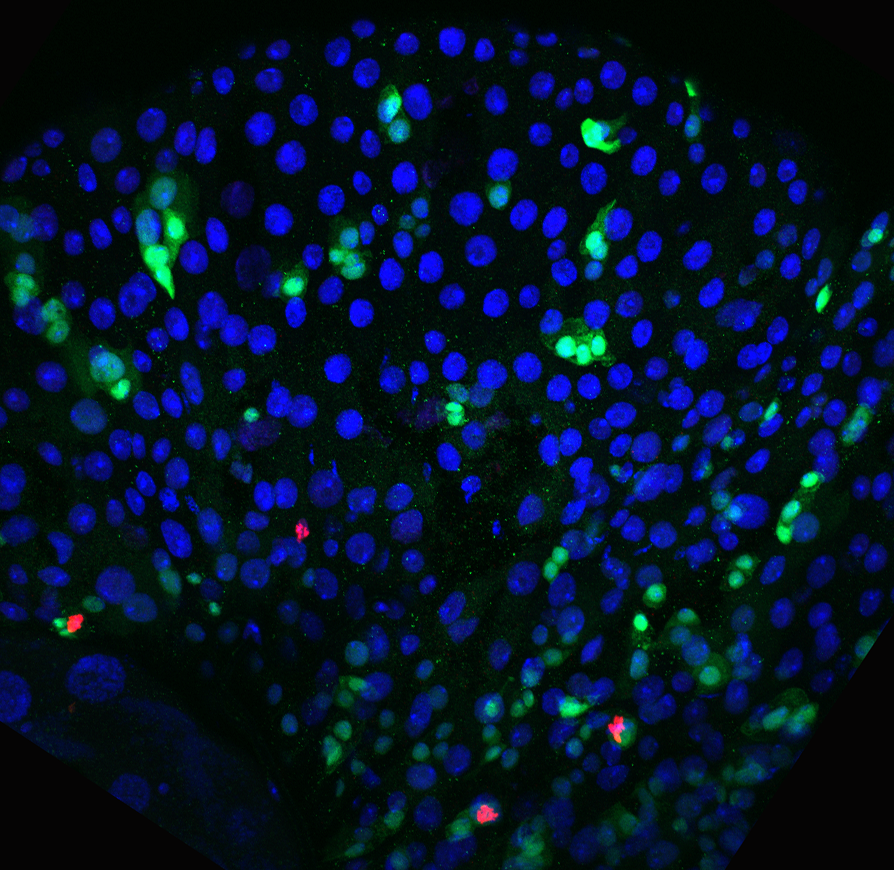

Supplement: Supplementary file 10 — Source data Fig. 7 [file 44319_2026_701_MOESM10_ESM.zip › Fig. 7/Fig. 7A-A'/Dlts-let7-decoy_GFP+phh3+DAPI.tif]

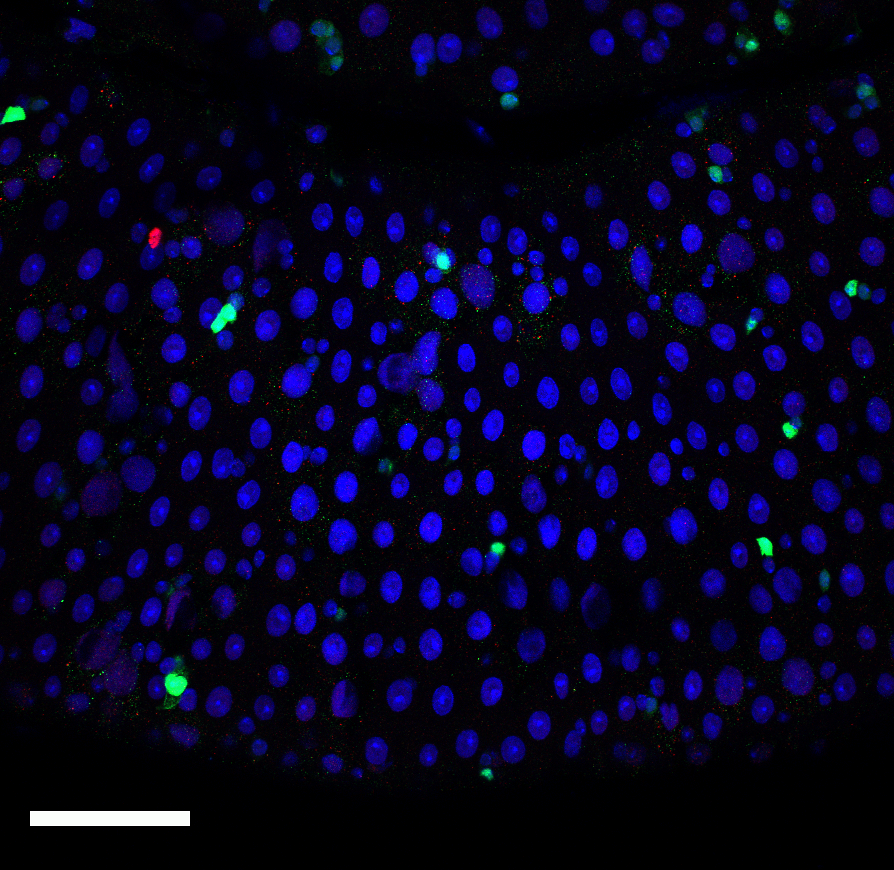

Supplement: Supplementary file 10 — Source data Fig. 7 [file 44319_2026_701_MOESM10_ESM.zip › Fig. 7/Fig. 7A-A'/Dlts-mcherryRNAi_GFP+ph3+DAPI.tif]

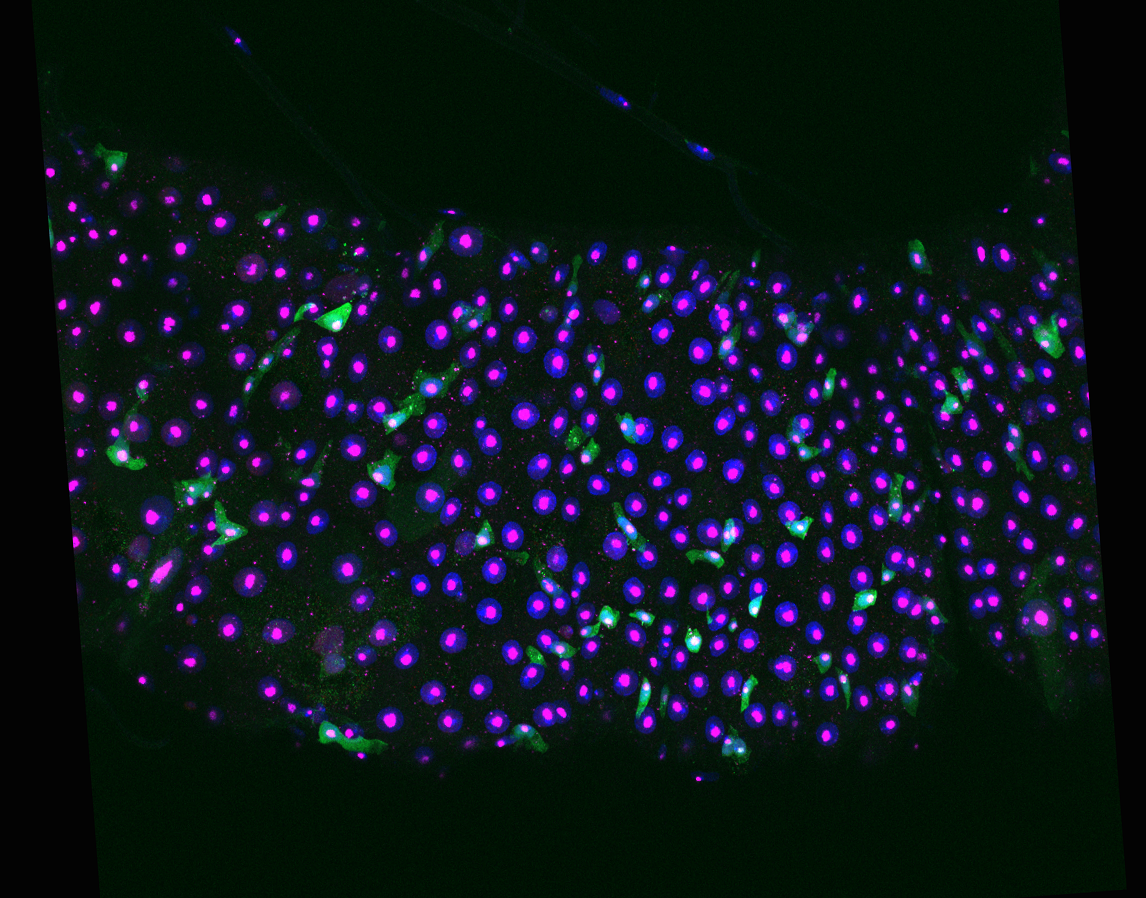

Supplement: Supplementary file 10 — Source data Fig. 7 [file 44319_2026_701_MOESM10_ESM.zip › Fig. 7/Fig. 7D-D'/esgts-dwdr4RNAi;uas-let7gfp+PHH3+dapi.tif]

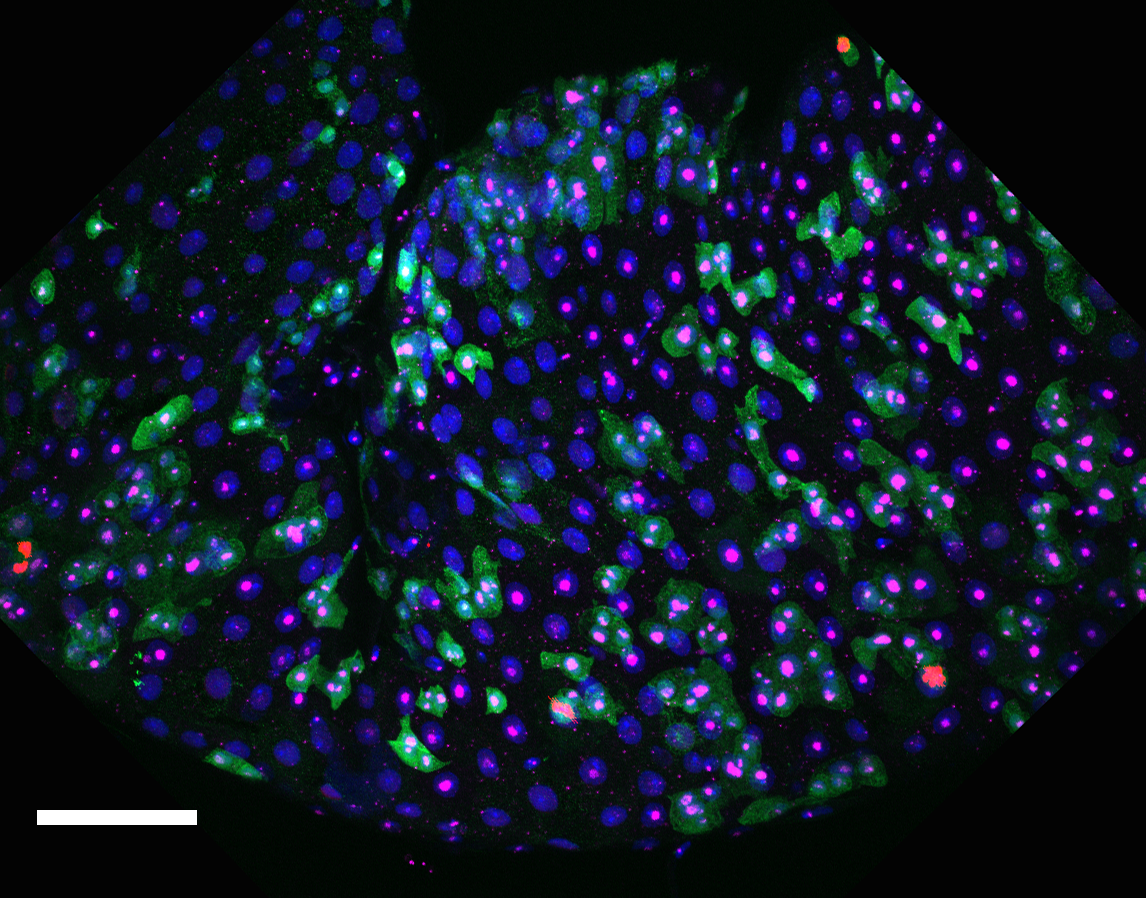

Supplement: Supplementary file 10 — Source data Fig. 7 [file 44319_2026_701_MOESM10_ESM.zip › Fig. 7/Fig. 7D-D'/esgts-dwdr4RNAiGFP+phh3+Fibrillarin+DAPI.tif]

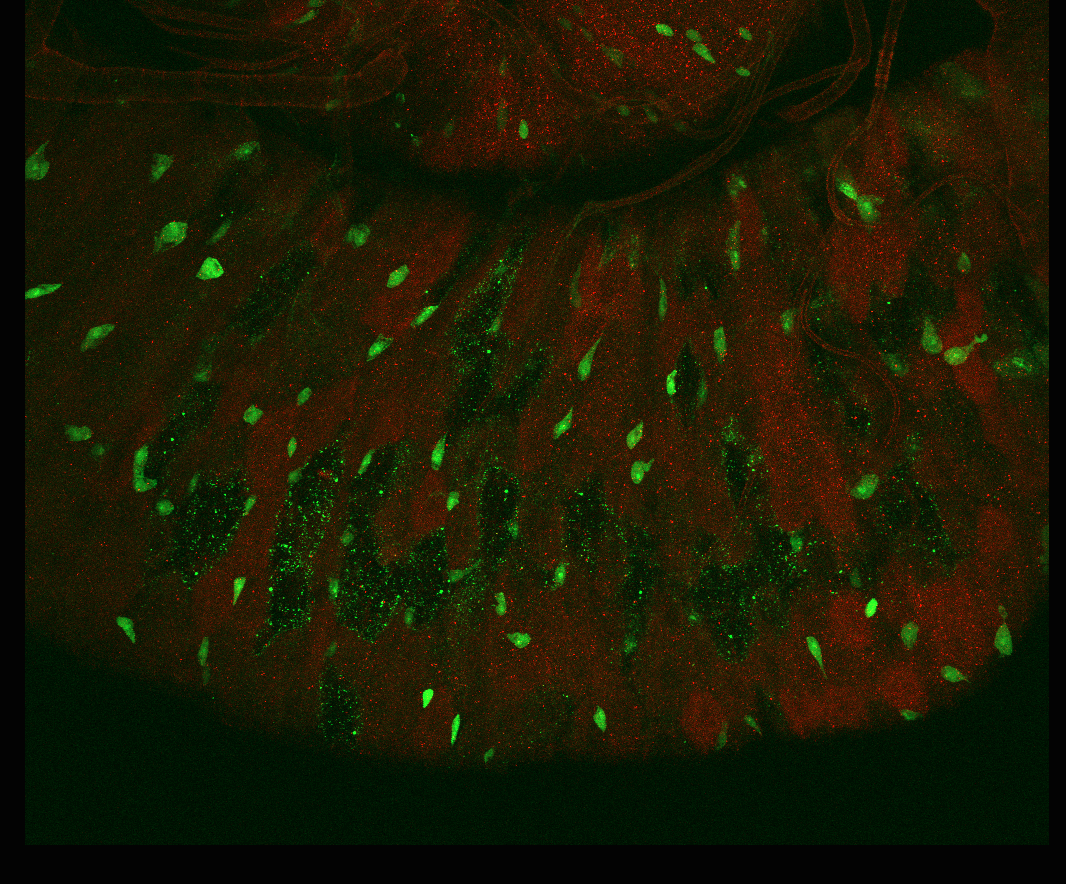

Supplement: Supplementary file 10 — Source data Fig. 7 [file 44319_2026_701_MOESM10_ESM.zip › Fig. 7/Fig. 7E-E'/esgts-dwdr4RNAi;uas-let7_GFP+p4ebp.tif]

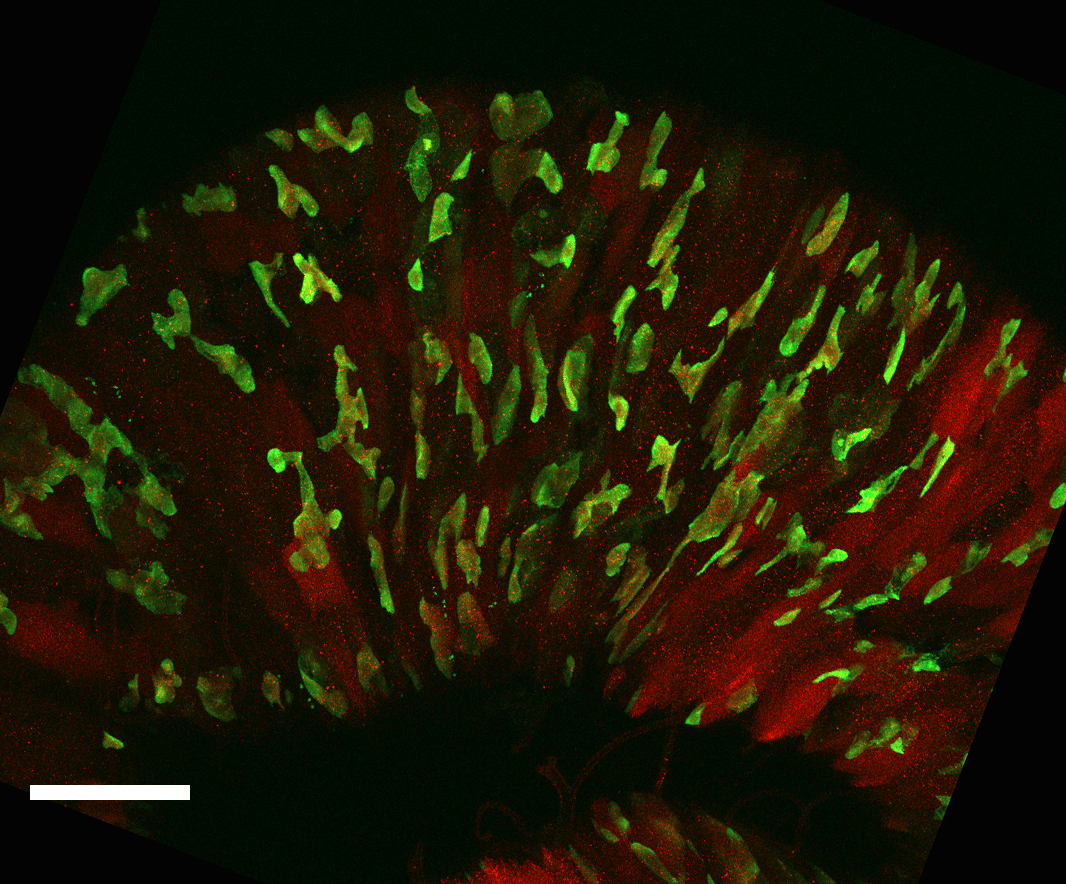

Supplement: Supplementary file 10 — Source data Fig. 7 [file 44319_2026_701_MOESM10_ESM.zip › Fig. 7/Fig. 7E-E'/esgts-dwdr4RNAi_p4EBP+GFP.tif]

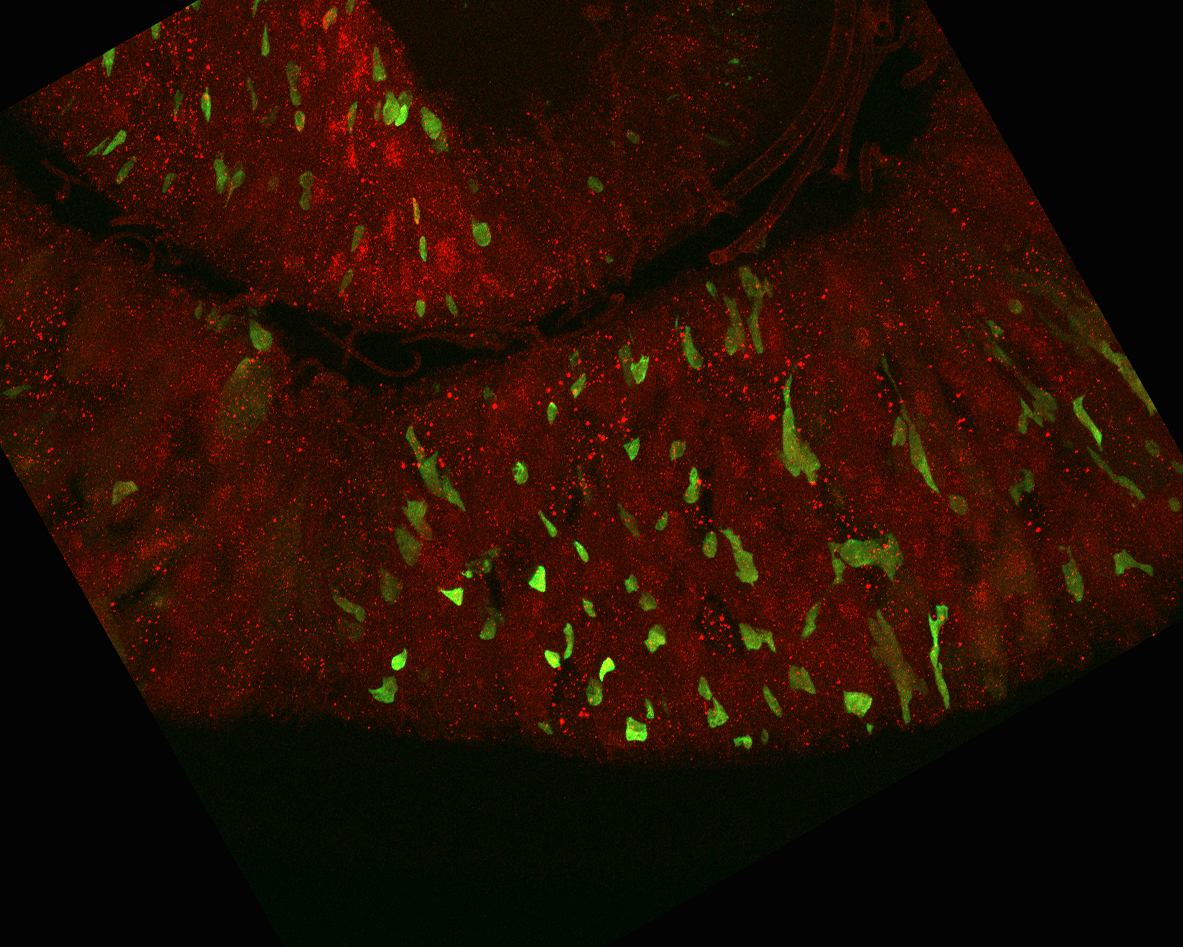

Supplement: Supplementary file 10 — Source data Fig. 7 [file 44319_2026_701_MOESM10_ESM.zip › Fig. 7/Fig. 7F-F'/esgts-dwdr4RNAi;uas-let7_GFP+pJNK.tif]

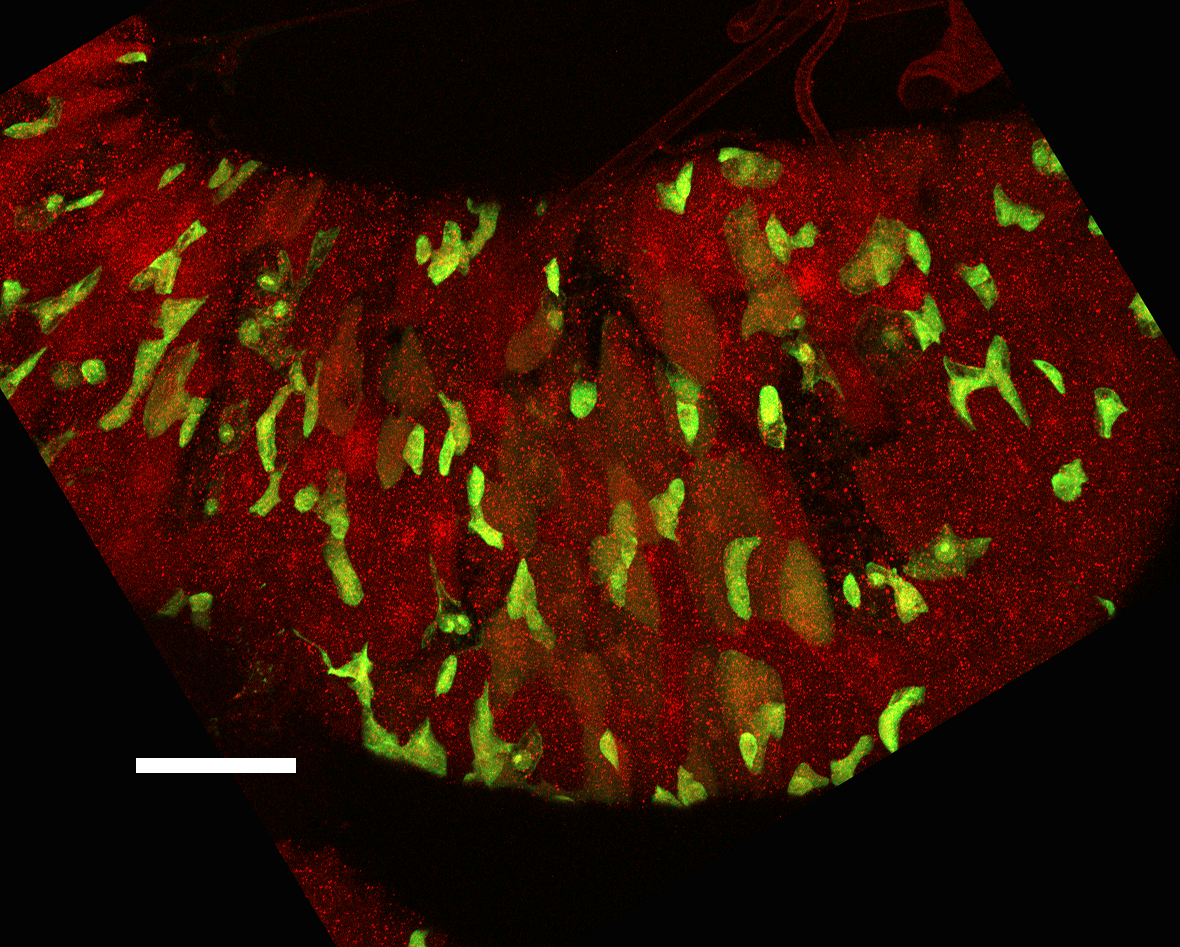

Supplement: Supplementary file 10 — Source data Fig. 7 [file 44319_2026_701_MOESM10_ESM.zip › Fig. 7/Fig. 7F-F'/esgts-dwdr4RNAi_GFP+pJNK.tif]

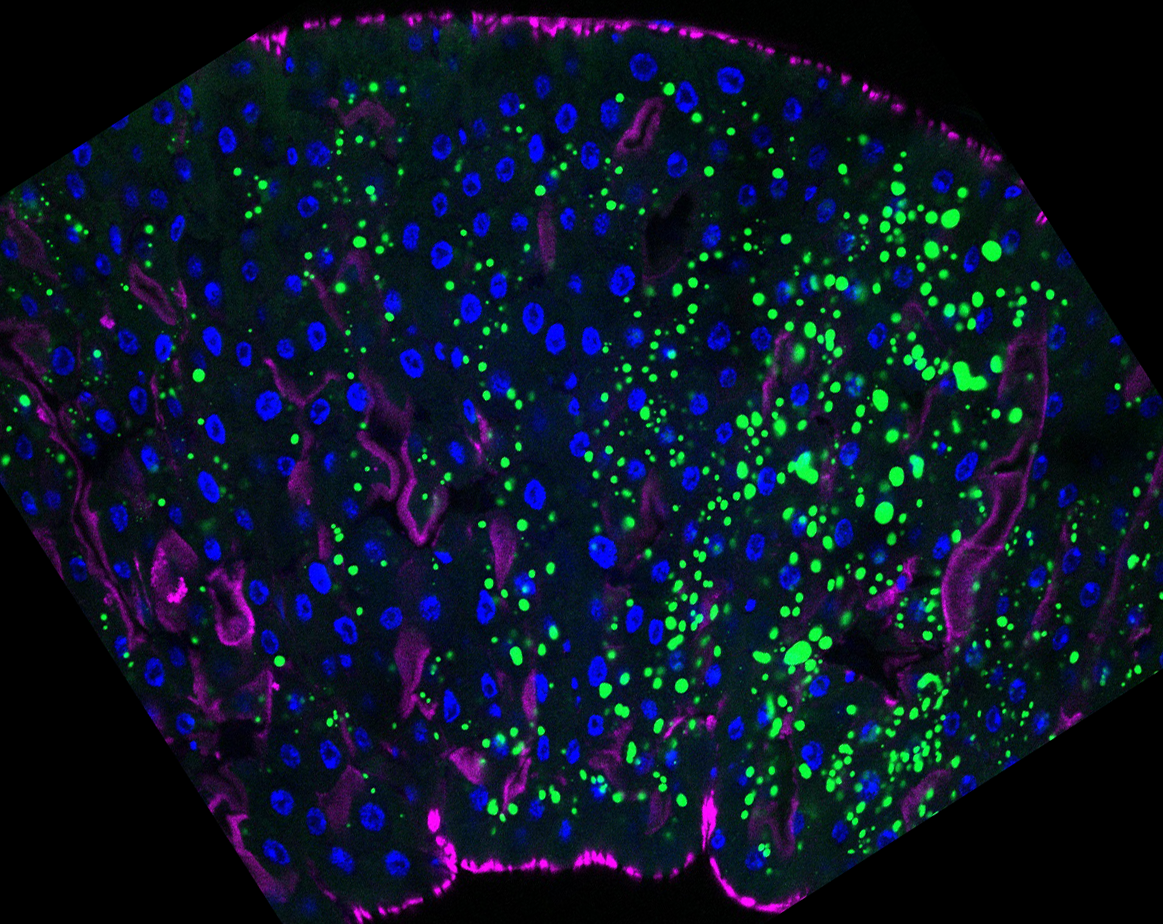

Supplement: Supplementary file 11 — Figure EV1 Source Data [file 44319_2026_701_MOESM11_ESM.zip › EV1/Fig. EV1E/wh7_Bodipy+Phalloidin+DAPI.tif]

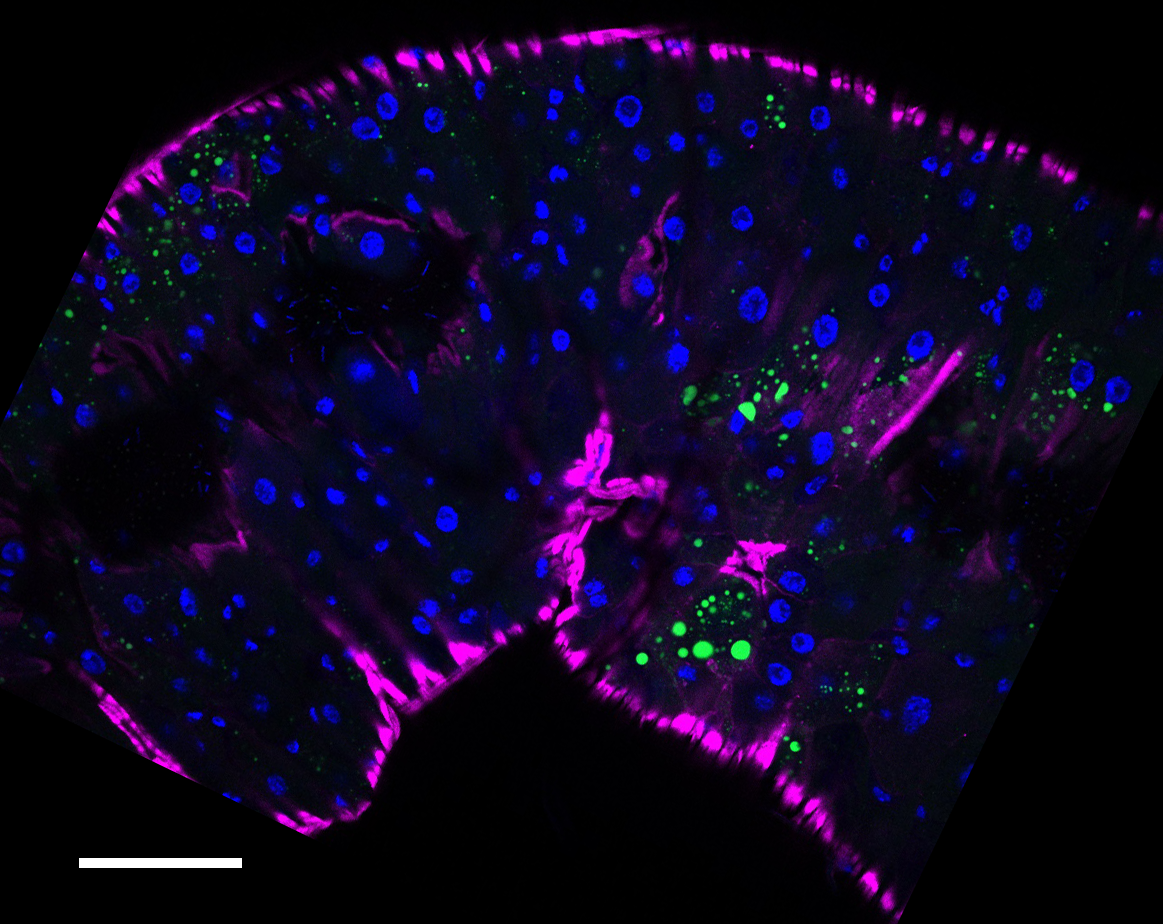

Supplement: Supplementary file 11 — Figure EV1 Source Data [file 44319_2026_701_MOESM11_ESM.zip › EV1/Fig. EV1E/WT_Bodipy+Phalloidin+DAPI.tif]

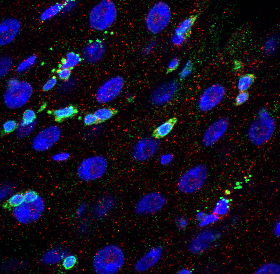

Supplement: Supplementary file 12 — Figure EV2 Source Data [file 44319_2026_701_MOESM12_ESM.zip › EV2/Fig. EV2A/esg-mcD8GFP_GFP+Hdc+DAPI.tif]

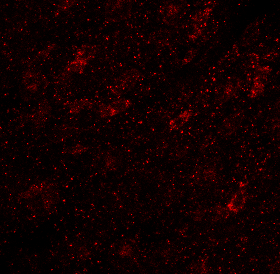

Supplement: Supplementary file 12 — Figure EV2 Source Data [file 44319_2026_701_MOESM12_ESM.zip › EV2/Fig. EV2A/esg-mcD8GFP_Hdc.tif]

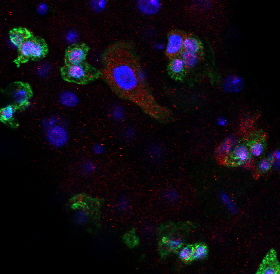

Supplement: Supplementary file 12 — Figure EV2 Source Data [file 44319_2026_701_MOESM12_ESM.zip › EV2/Fig. EV2A/wh7;esg-mcD8GFP_GFP+Hdc+DAPI.tif]

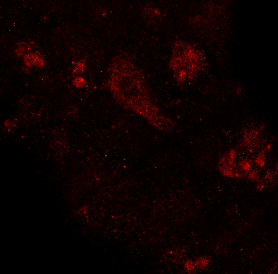

Supplement: Supplementary file 12 — Figure EV2 Source Data [file 44319_2026_701_MOESM12_ESM.zip › EV2/Fig. EV2A/wh7;esg-mcD8GFP_Hdc.tif]

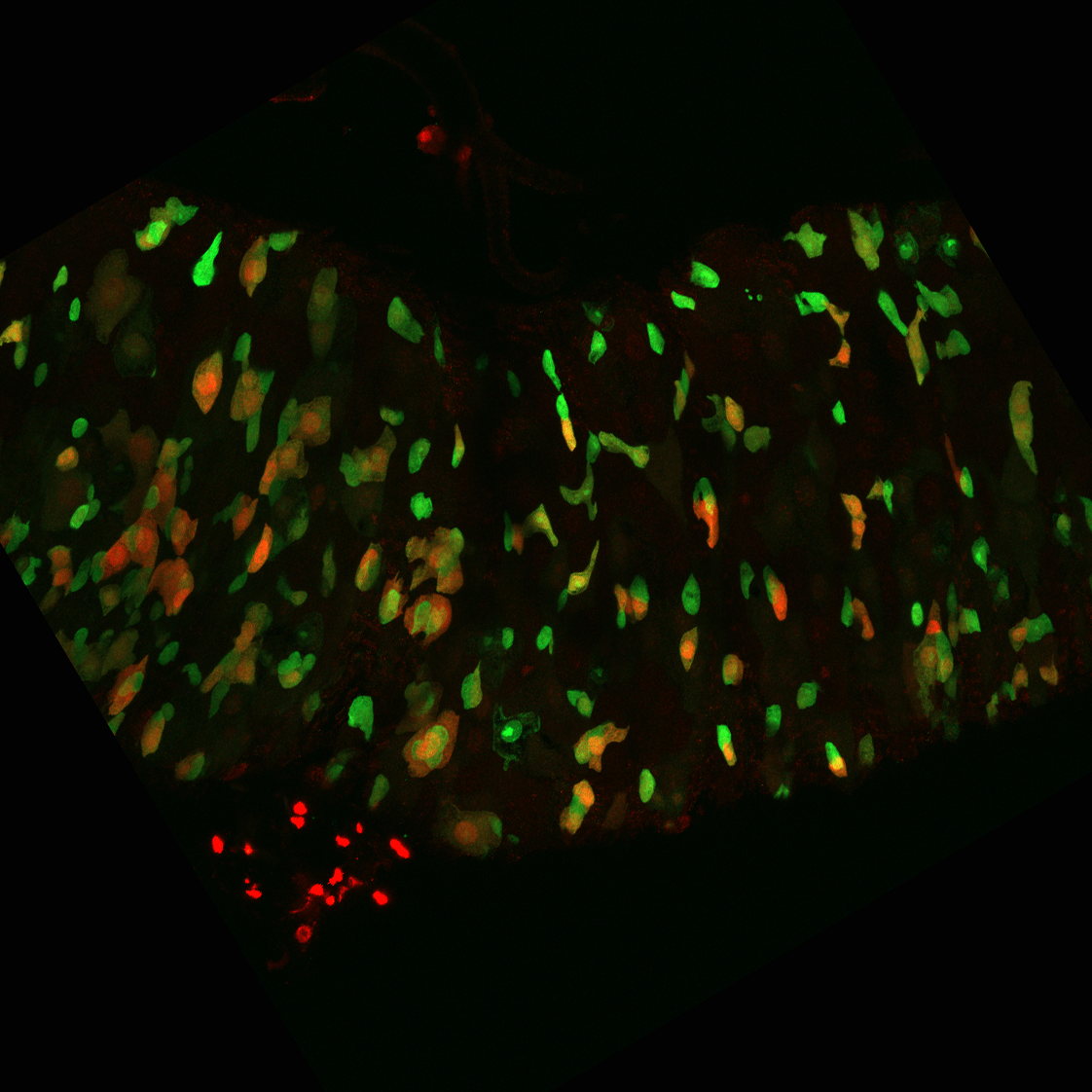

Supplement: Supplementary file 12 — Figure EV2 Source Data [file 44319_2026_701_MOESM12_ESM.zip › EV2/Fig. EV2C/esgts-dwdr4RNAi_GFP+LacZ.tif]

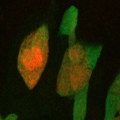

Supplement: Supplementary file 12 — Figure EV2 Source Data [file 44319_2026_701_MOESM12_ESM.zip › EV2/Fig. EV2C/esgts-dwdr4RNAi_GFP+LacZ_cut.tif]

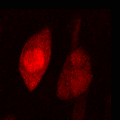

Supplement: Supplementary file 12 — Figure EV2 Source Data [file 44319_2026_701_MOESM12_ESM.zip › EV2/Fig. EV2C/esgts-dwdr4RNAi_lacZ_cut.tif]

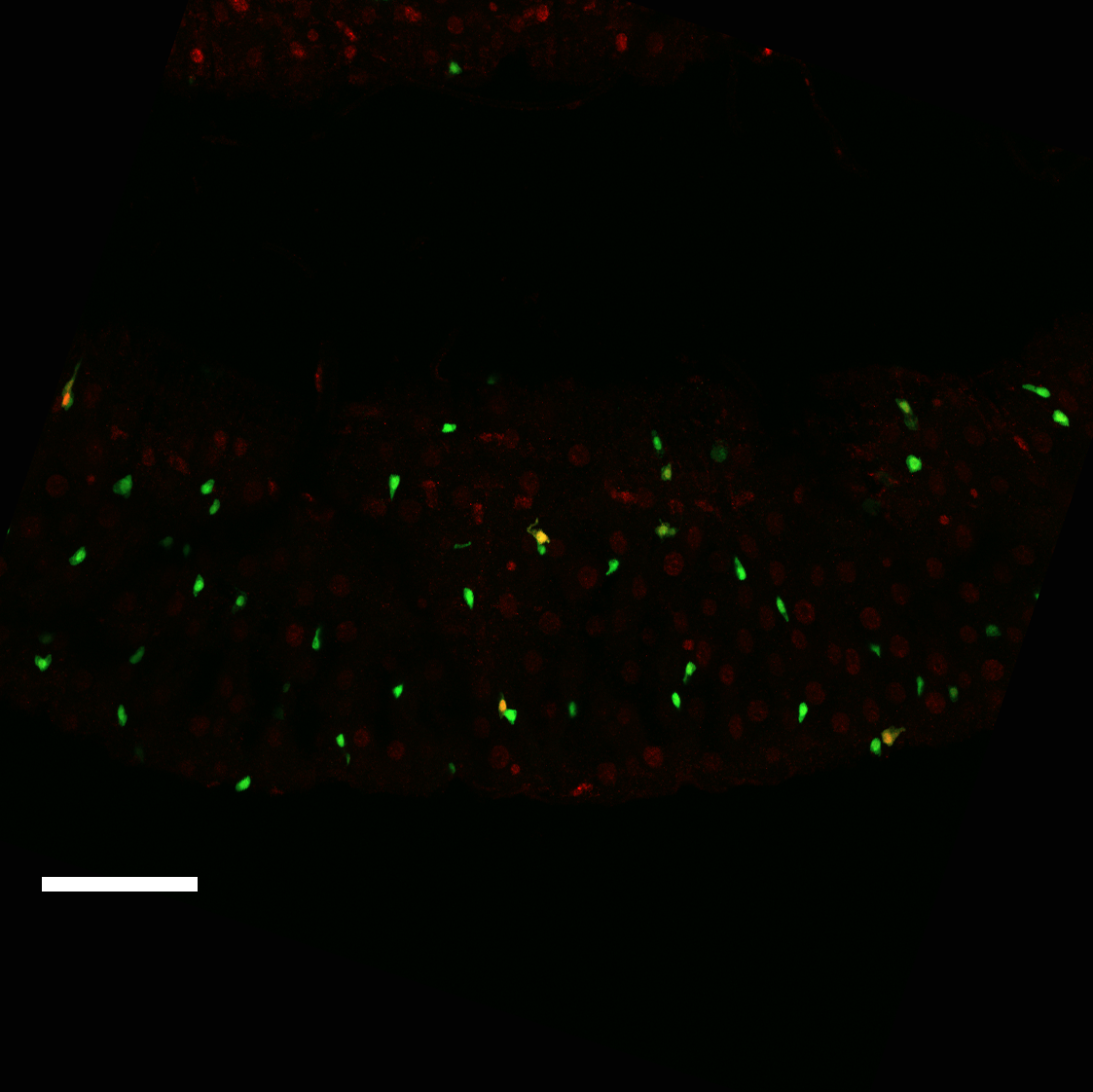

Supplement: Supplementary file 12 — Figure EV2 Source Data [file 44319_2026_701_MOESM12_ESM.zip › EV2/Fig. EV2C/esgts-mcherryRNAi_GFP+lacZ.tif]

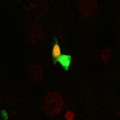

Supplement: Supplementary file 12 — Figure EV2 Source Data [file 44319_2026_701_MOESM12_ESM.zip › EV2/Fig. EV2C/esgts-mcherryRNAi_GFP+lacZ_cut.tif]

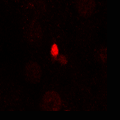

Supplement: Supplementary file 12 — Figure EV2 Source Data [file 44319_2026_701_MOESM12_ESM.zip › EV2/Fig. EV2C/esgts-mcherryRNAi_lacZ_cut.tif]

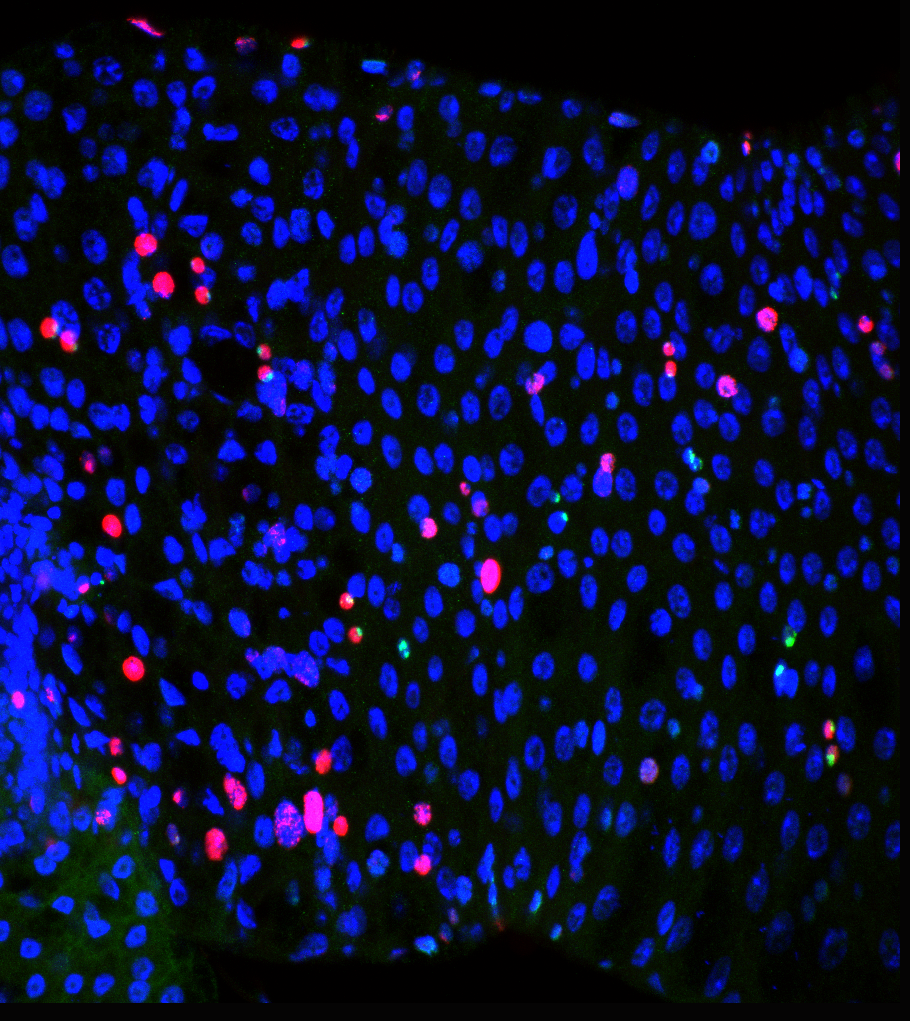

Supplement: Supplementary file 12 — Figure EV2 Source Data [file 44319_2026_701_MOESM12_ESM.zip › EV2/Fig. EV2D-D'/esg-dwdr4RNAi_phh3+EdU+DAPI.tif]

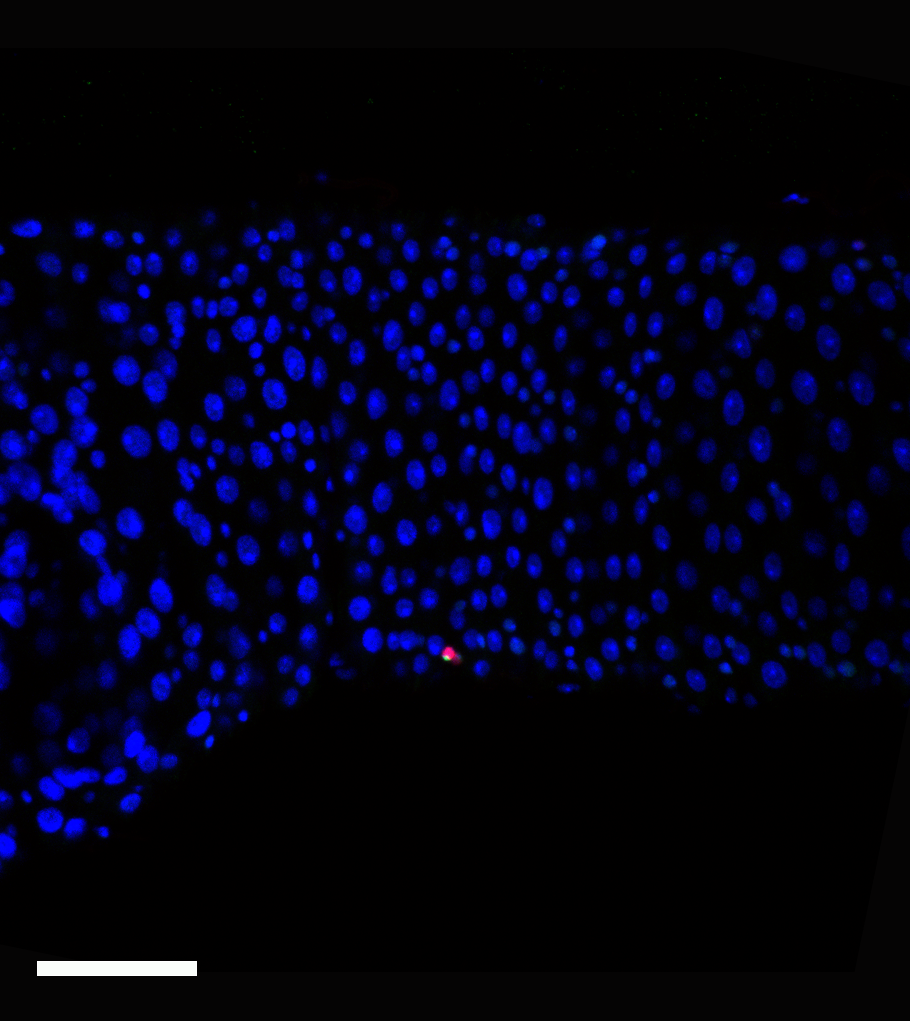

Supplement: Supplementary file 12 — Figure EV2 Source Data [file 44319_2026_701_MOESM12_ESM.zip › EV2/Fig. EV2D-D'/esg-egfpRNAi_phh3+EdU+DAPI.tif]

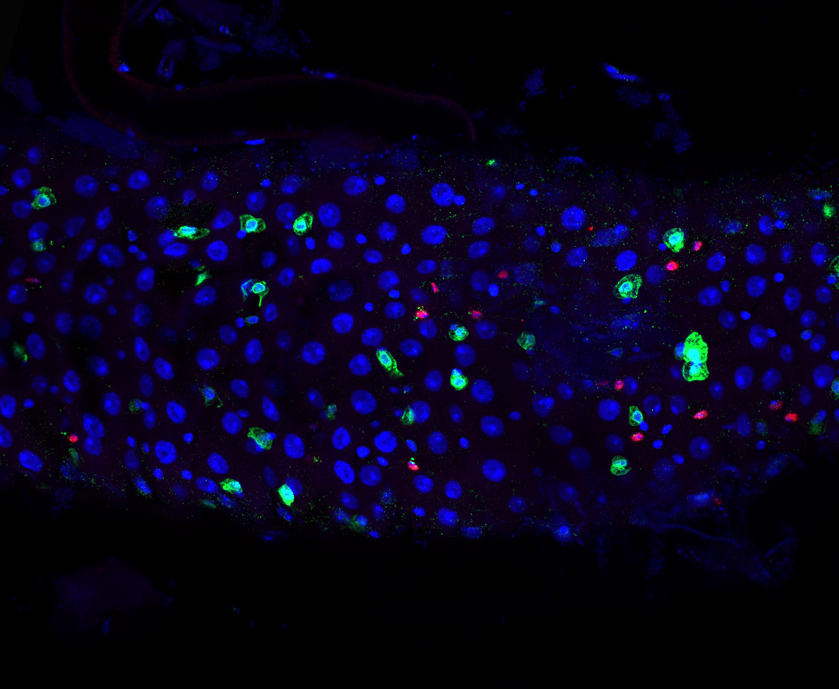

Supplement: Supplementary file 12 — Figure EV2 Source Data [file 44319_2026_701_MOESM12_ESM.zip › EV2/Fig. EV2E/Su(H)ts-dwdr4RNAi_GFP+Pros+DAPI.tif]

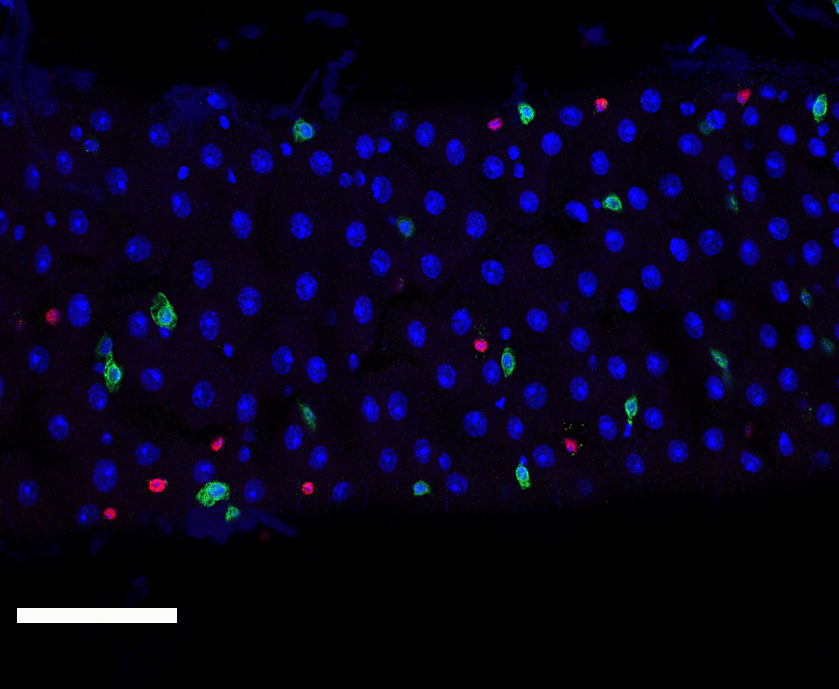

Supplement: Supplementary file 12 — Figure EV2 Source Data [file 44319_2026_701_MOESM12_ESM.zip › EV2/Fig. EV2E/Su(H)ts-mcherryRNAi_GFP+Pros+DAPI.tif]

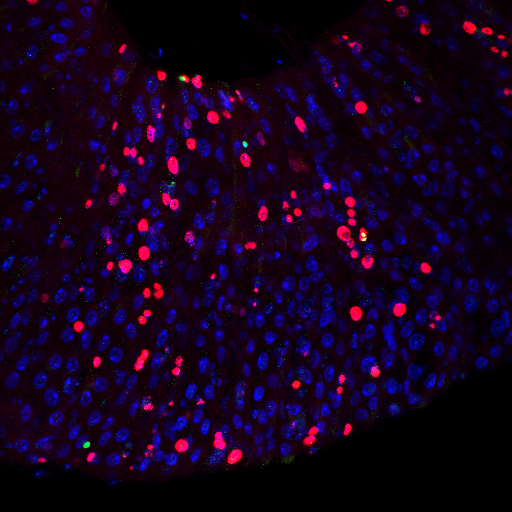

Supplement: Supplementary file 12 — Figure EV2 Source Data [file 44319_2026_701_MOESM12_ESM.zip › EV2/Fig. EV2F-F'/NP1-dwdr4RNAi_pHH3+Edu+DAPI.tif]

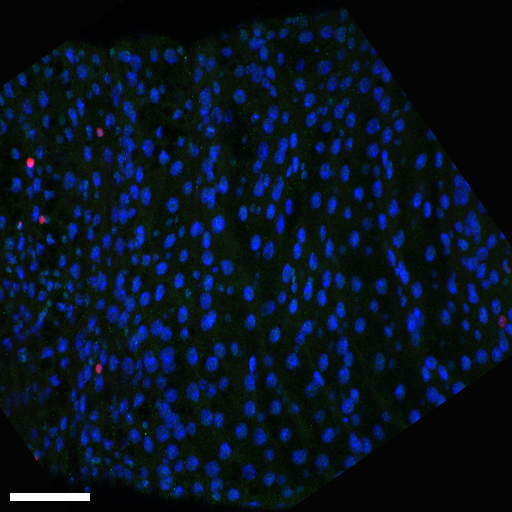

Supplement: Supplementary file 12 — Figure EV2 Source Data [file 44319_2026_701_MOESM12_ESM.zip › EV2/Fig. EV2F-F'/NP1-egfpRNAi_EdU+phh3+DAPI.tif]

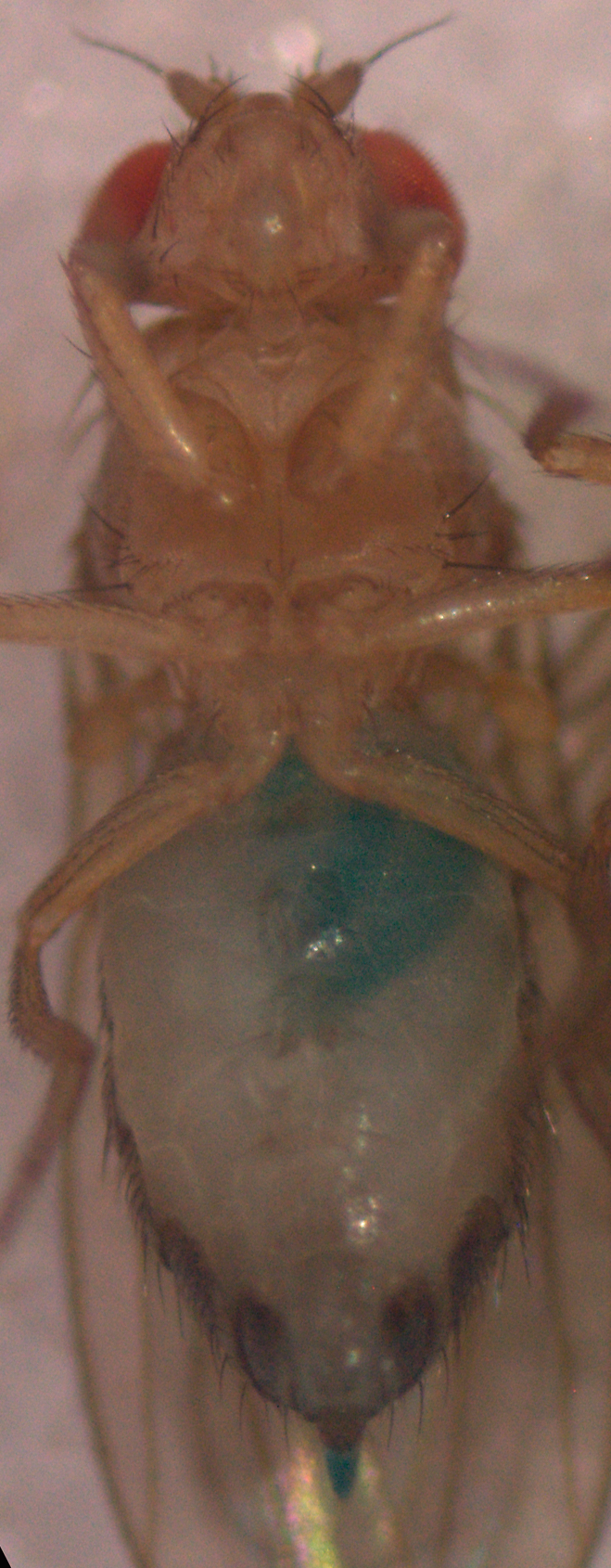

Supplement: Supplementary file 12 — Figure EV2 Source Data [file 44319_2026_701_MOESM12_ESM.zip › EV2/Fig. EV2G/Dlts-dwdr4RNAi.tif]

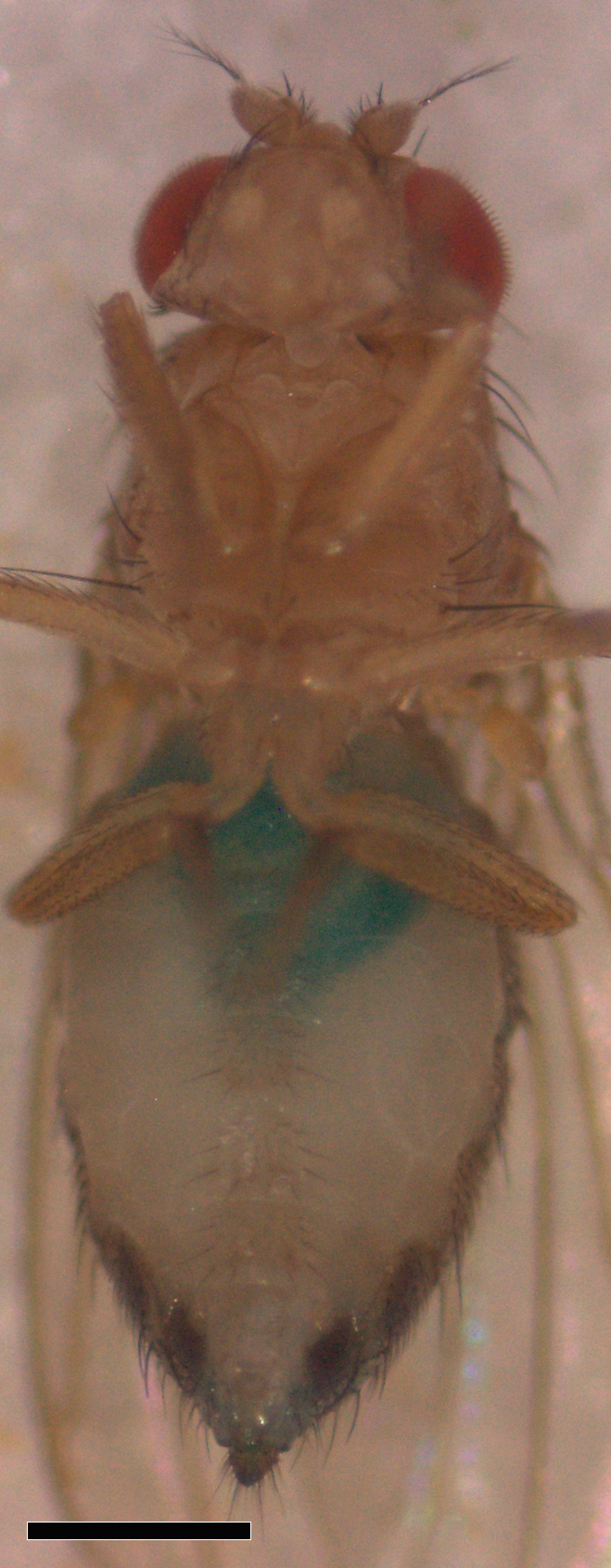

Supplement: Supplementary file 12 — Figure EV2 Source Data [file 44319_2026_701_MOESM12_ESM.zip › EV2/Fig. EV2G/Dlts-mcherryRNAi.tif]

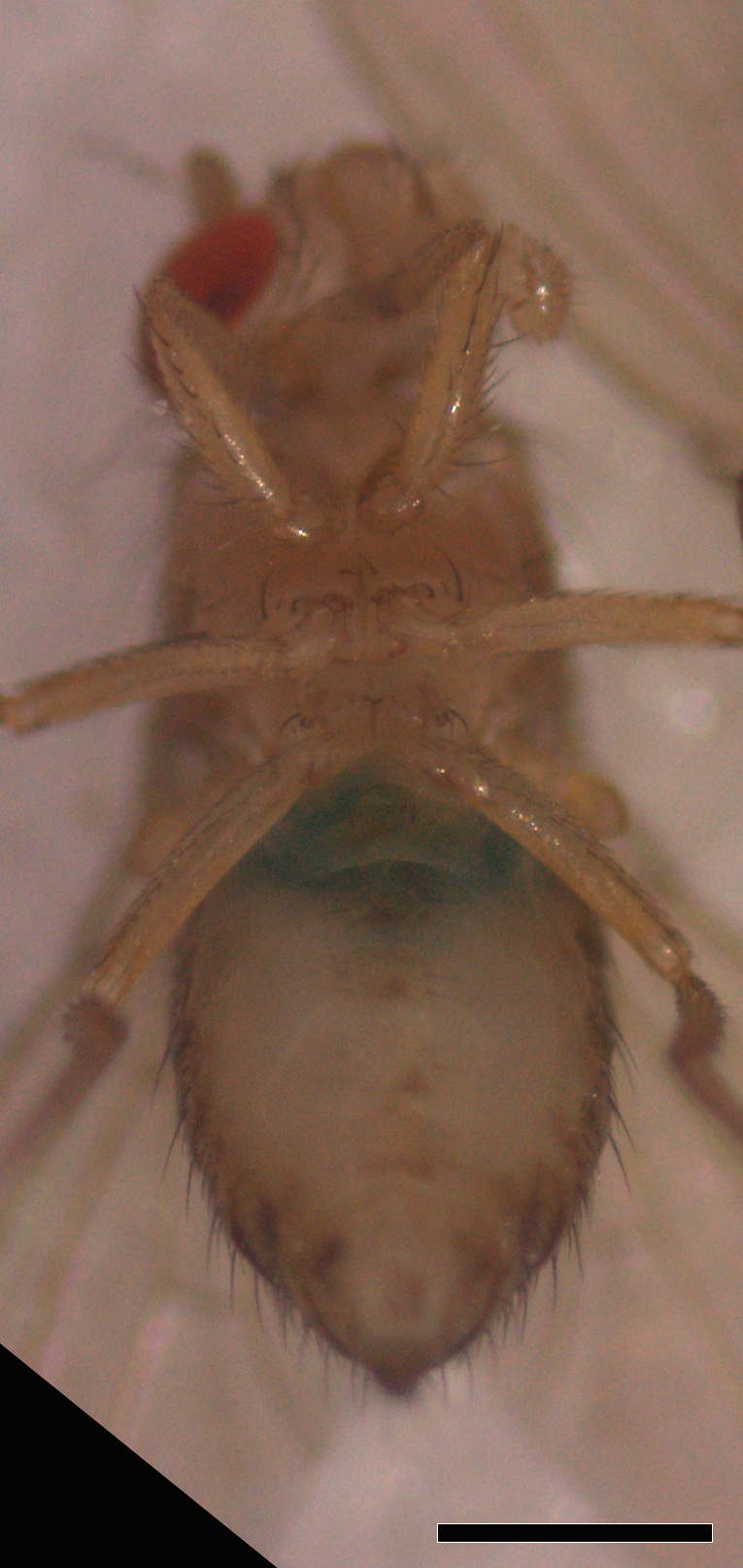

Supplement: Supplementary file 12 — Figure EV2 Source Data [file 44319_2026_701_MOESM12_ESM.zip › EV2/Fig. EV2G/How-mcherryRNAi.tif]

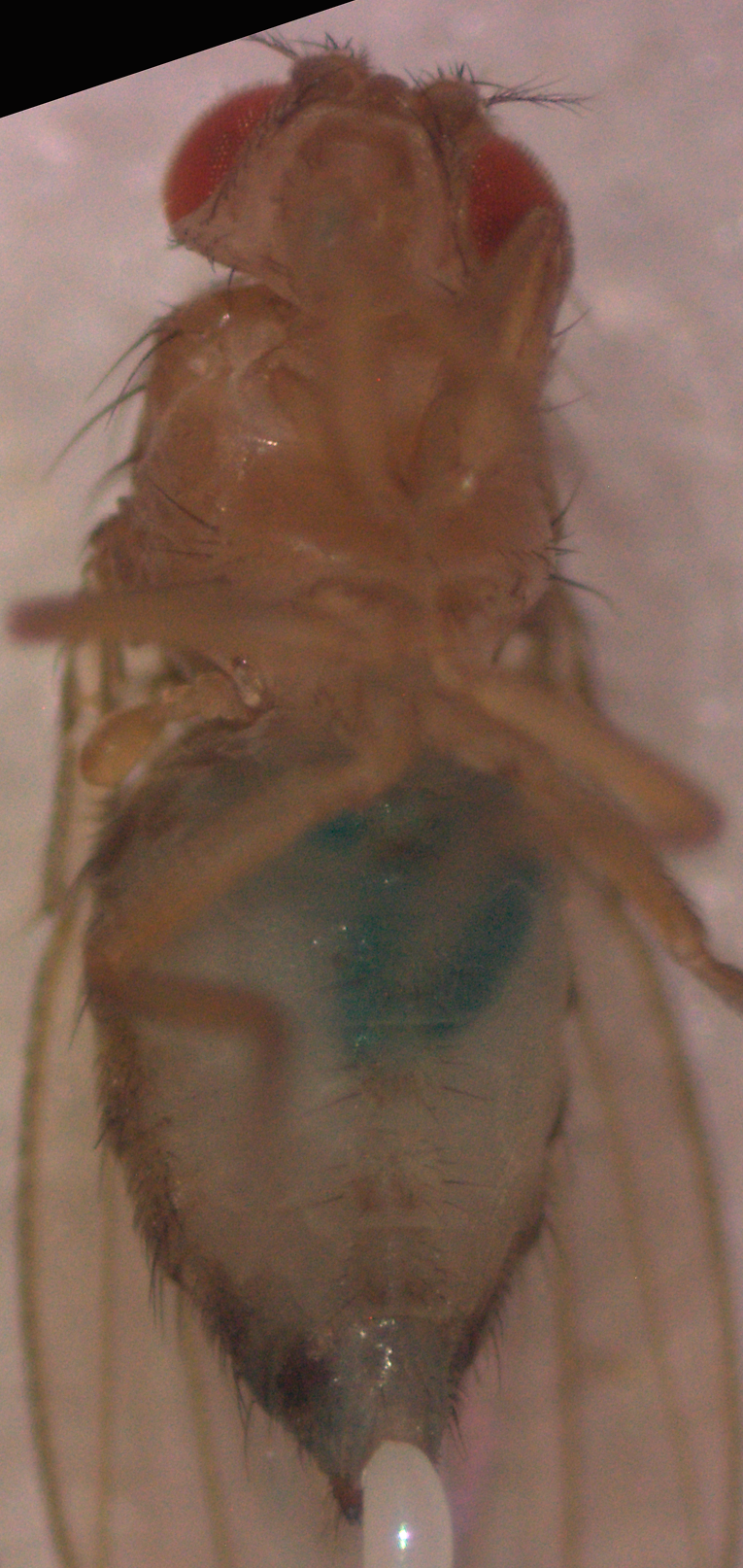

Supplement: Supplementary file 12 — Figure EV2 Source Data [file 44319_2026_701_MOESM12_ESM.zip › EV2/Fig. EV2G/How-Uasp-dwdr4RNAi.tif]

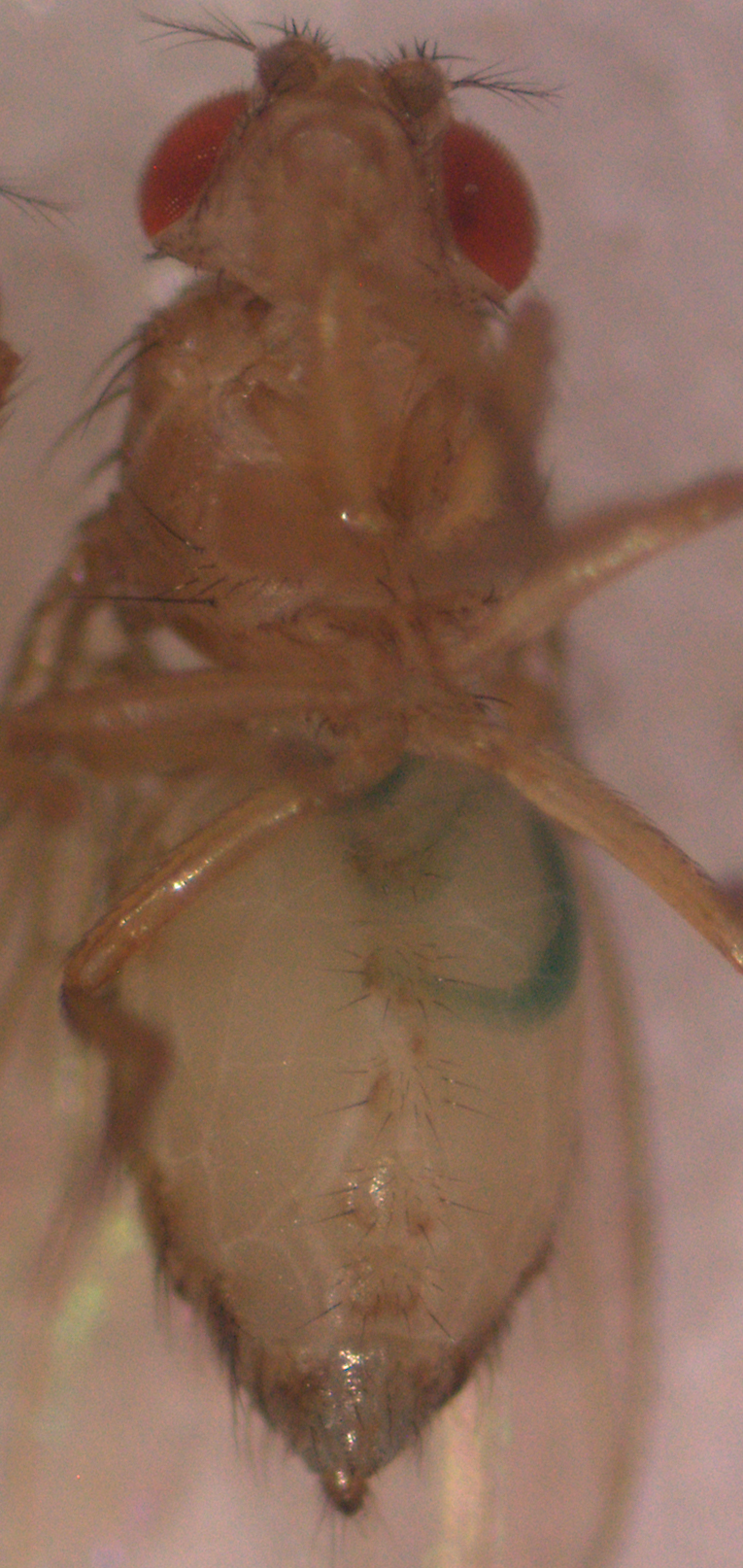

Supplement: Supplementary file 12 — Figure EV2 Source Data [file 44319_2026_701_MOESM12_ESM.zip › EV2/Fig. EV2G/How-Uast-dwdr4RNAi.tif]

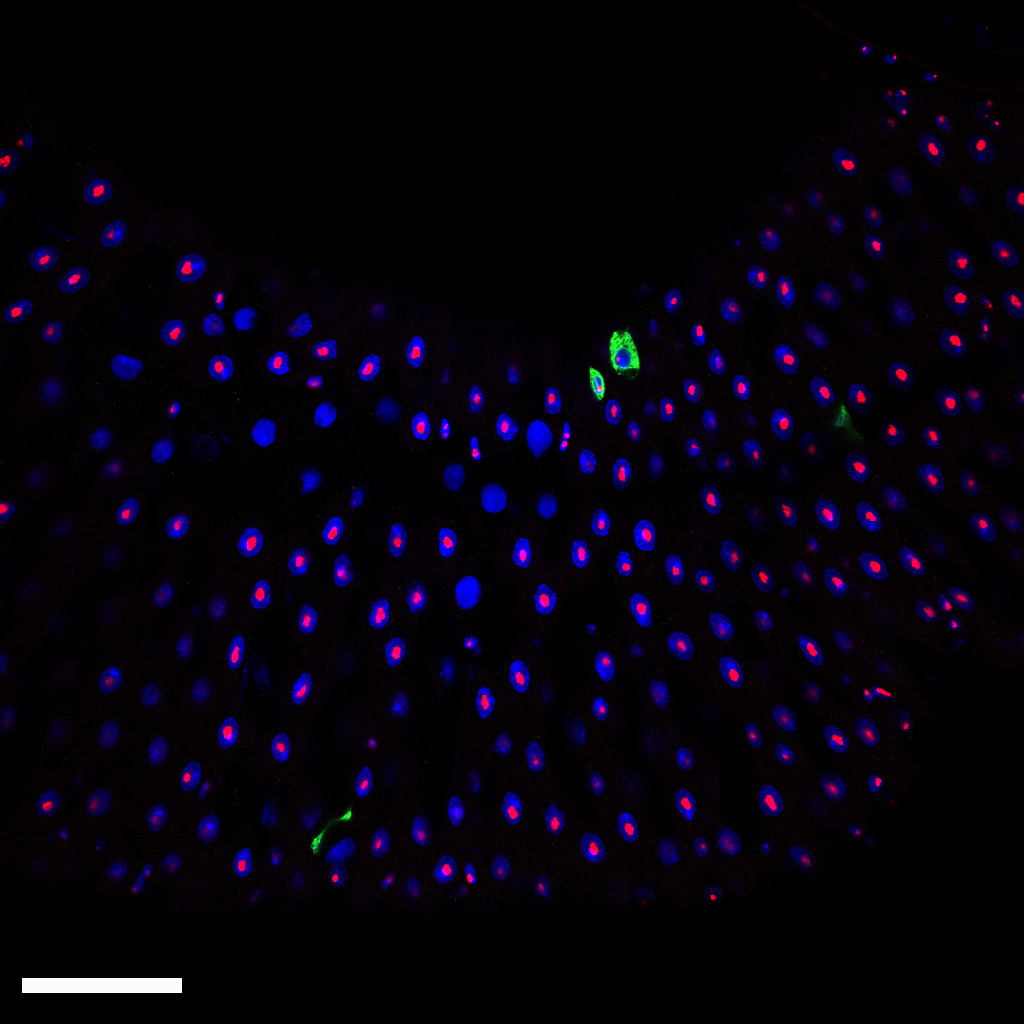

Supplement: Supplementary file 13 — Figure EV3 Source Data [file 44319_2026_701_MOESM13_ESM.zip › EV3/Fig. EV3A-A'/FRT19A_GFP+Fibrillarin+DAPI.tif]

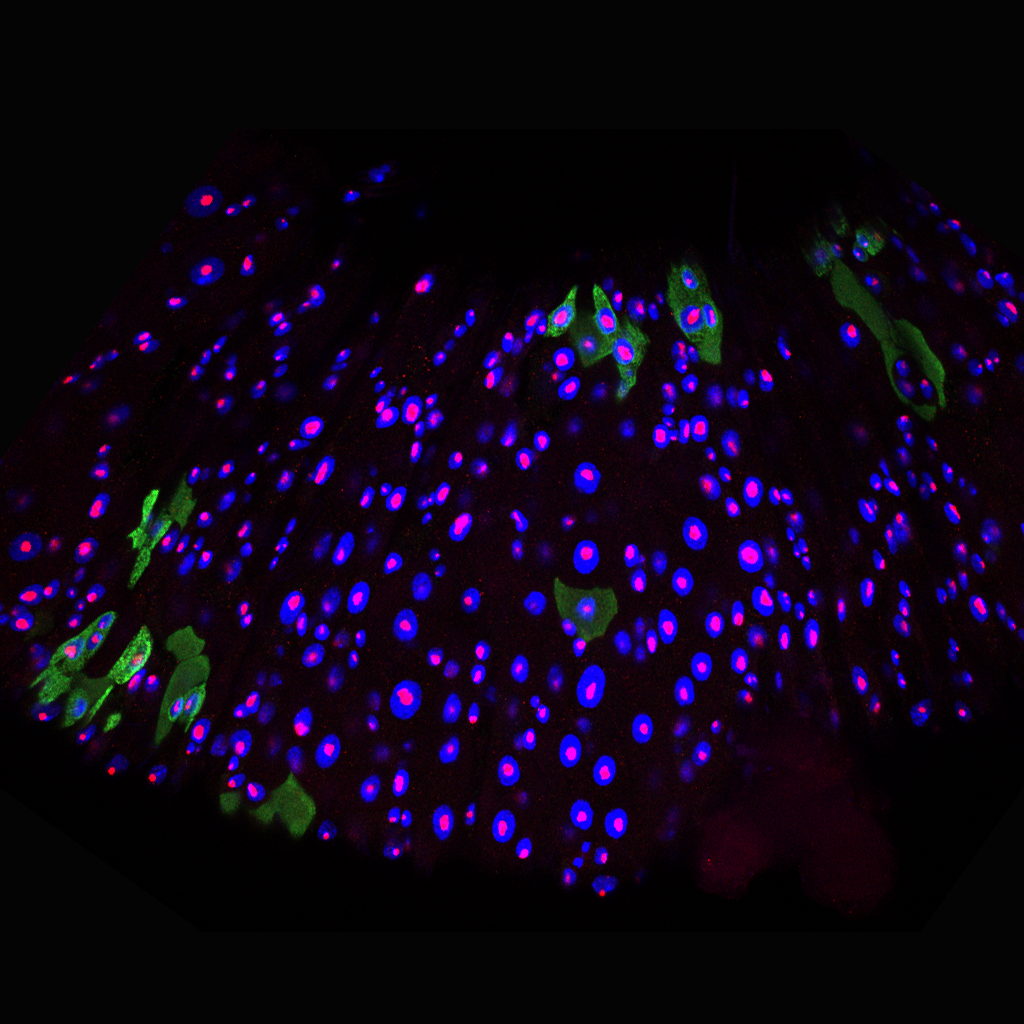

Supplement: Supplementary file 13 — Figure EV3 Source Data [file 44319_2026_701_MOESM13_ESM.zip › EV3/Fig. EV3A-A'/wh7FRT19A_GFP+Fibrillarin +DAPI.tif]

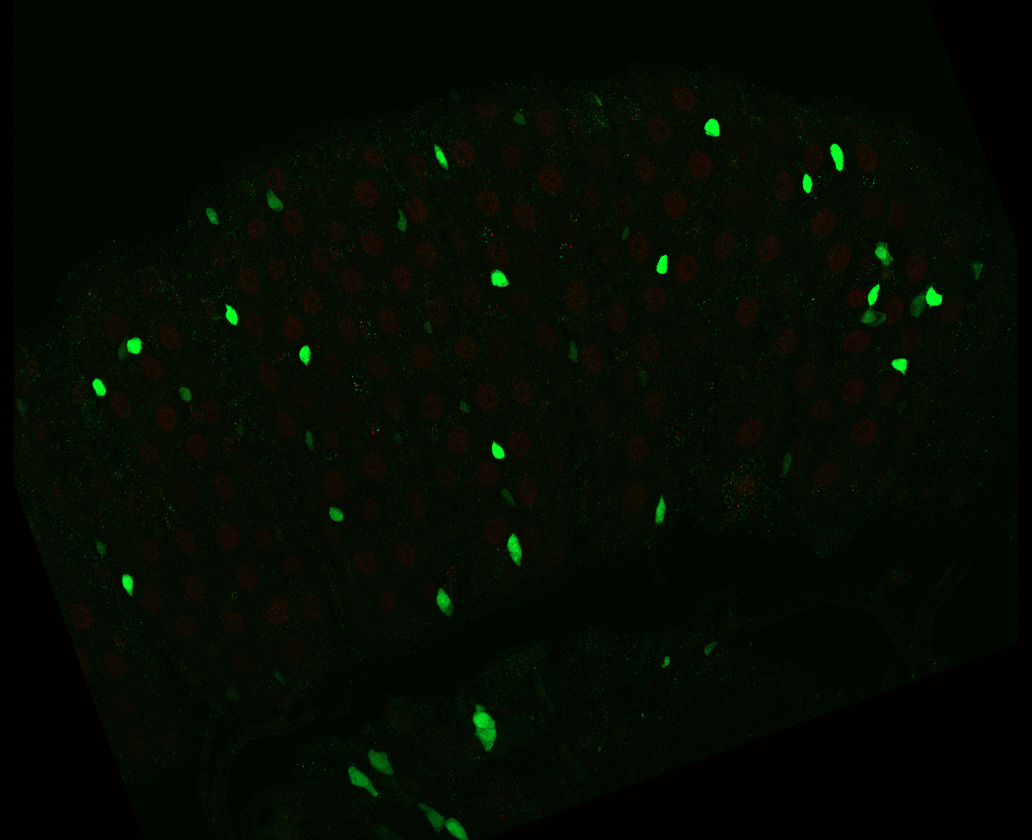

Supplement: Supplementary file 13 — Figure EV3 Source Data [file 44319_2026_701_MOESM13_ESM.zip › EV3/Fig. EV3B-B'/esgt-dwdr4RNAi_GFP+phh3_With Rapamycin.tif]

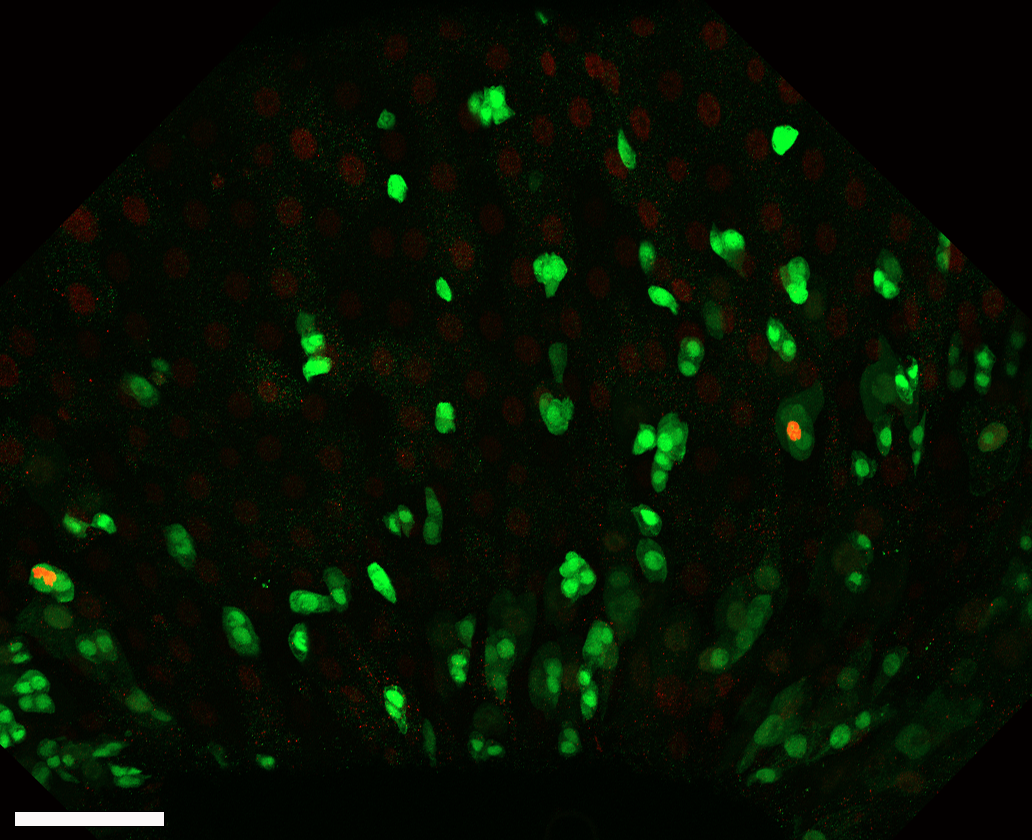

Supplement: Supplementary file 13 — Figure EV3 Source Data [file 44319_2026_701_MOESM13_ESM.zip › EV3/Fig. EV3B-B'/esgts-dwdr4RNAi_GFP+phh3_without Rapamycin.tif]

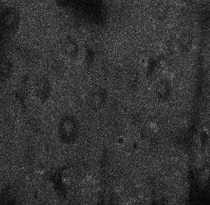

Supplement: Supplementary file 14 — Figure EV4 Source Data [file 44319_2026_701_MOESM14_ESM.zip › EV4/Fig. EV4B-B'/esg-mcD8GFP_DHE_cut.tif]

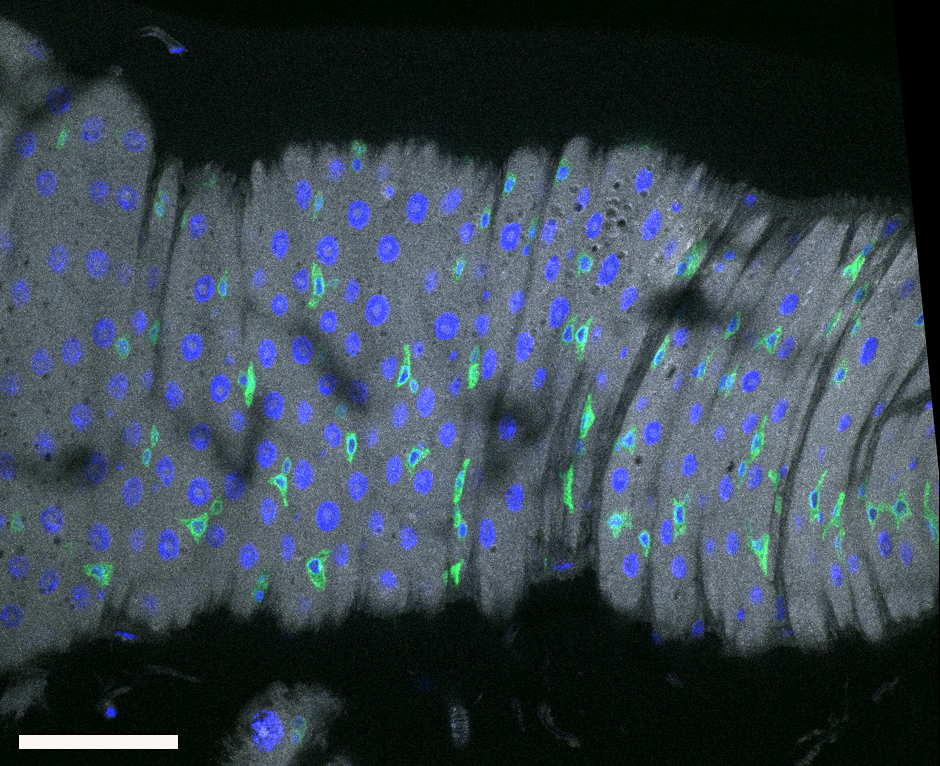

Supplement: Supplementary file 14 — Figure EV4 Source Data [file 44319_2026_701_MOESM14_ESM.zip › EV4/Fig. EV4B-B'/esg-mcD8gfp_GFP+DHE+DAPI.tif]

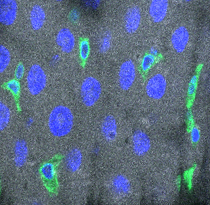

Supplement: Supplementary file 14 — Figure EV4 Source Data [file 44319_2026_701_MOESM14_ESM.zip › EV4/Fig. EV4B-B'/esg-mcD8GFP_GFP+DHE+DAPI_cut.tif]

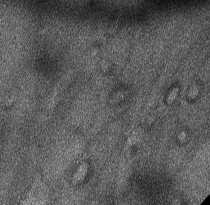

Supplement: Supplementary file 14 — Figure EV4 Source Data [file 44319_2026_701_MOESM14_ESM.zip › EV4/Fig. EV4B-B'/wh7;esg-mcD8GFP_DHE_cut.tif]

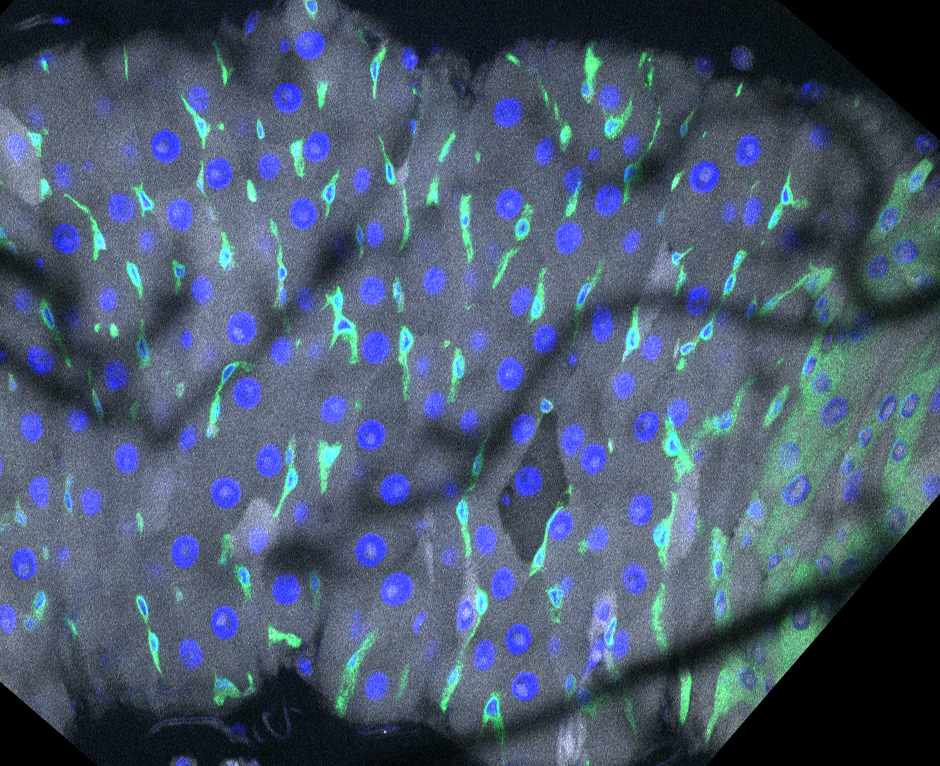

Supplement: Supplementary file 14 — Figure EV4 Source Data [file 44319_2026_701_MOESM14_ESM.zip › EV4/Fig. EV4B-B'/wh7;esg-mcD8GFP_GFP+DHE+DAPI.tif]

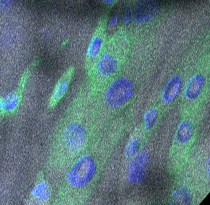

Supplement: Supplementary file 14 — Figure EV4 Source Data [file 44319_2026_701_MOESM14_ESM.zip › EV4/Fig. EV4B-B'/wh7;esg-mcD8GFP_GFP+DHE+DAPI_cut.tif]
