# Supplementary figures and images for: Wdr4 regulates ribosome biogenesis and intestinal homeostasis via let-7 (part 2 of 2)
Source: EMBO Rep. 2026 Feb 9;27(8):1870–903. doi: 10.1038/s44319-026-00701-y (PMC13121520; doi:10.1038/s44319-026-00701-y)

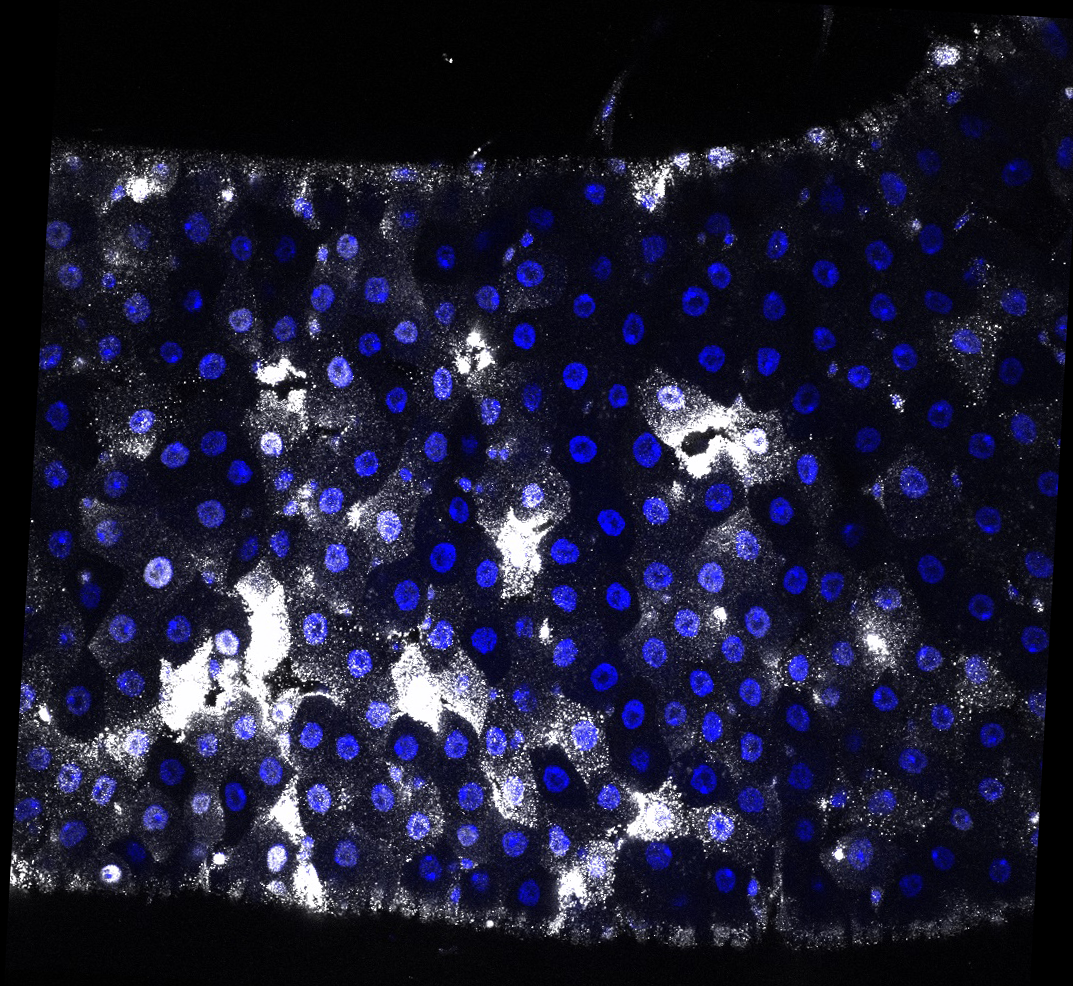

Supplement: Supplementary file 14 — Figure EV4 Source Data [file 44319_2026_701_MOESM14_ESM.zip › EV4/Fig. EV4C/wh7_LacZ+DAPI.tif]

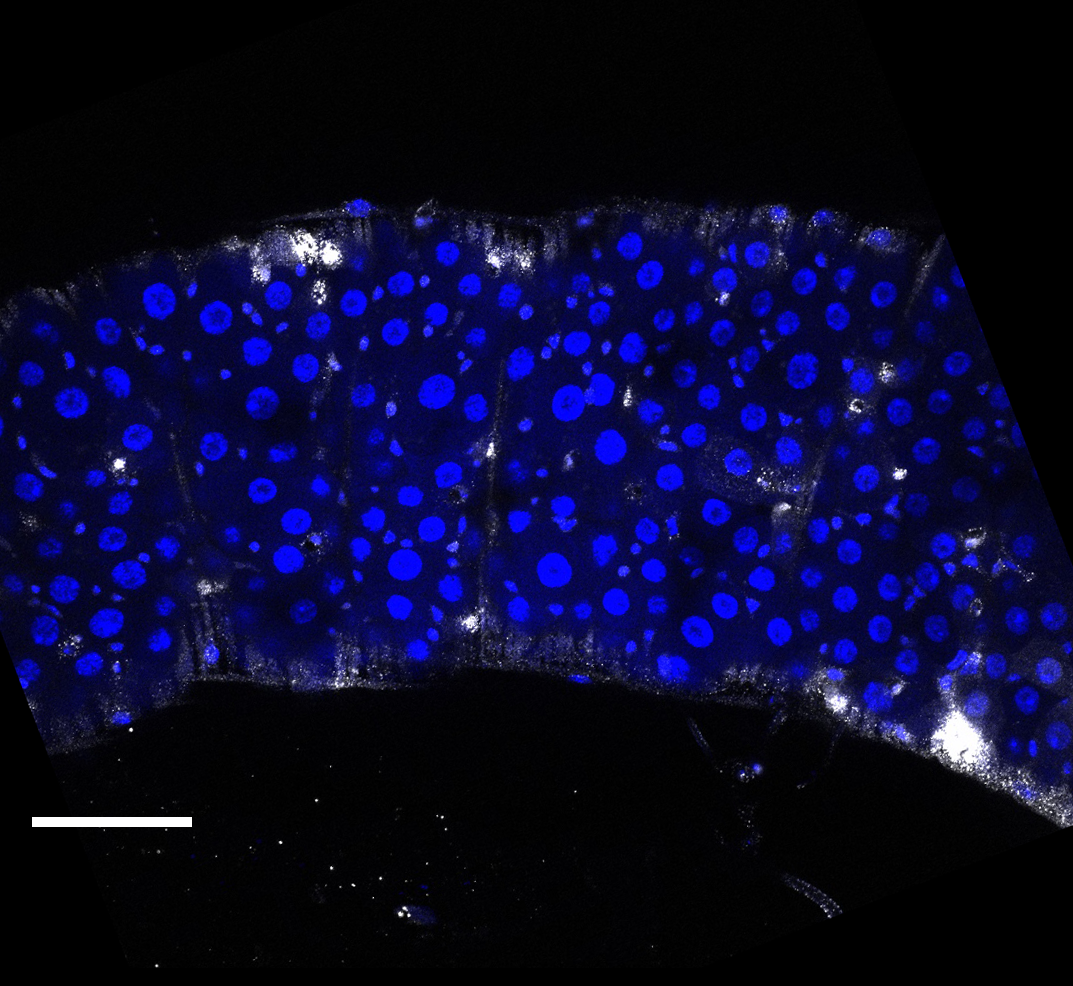

Supplement: Supplementary file 14 — Figure EV4 Source Data [file 44319_2026_701_MOESM14_ESM.zip › EV4/Fig. EV4C/WT_LacZ+DAPI.tif]

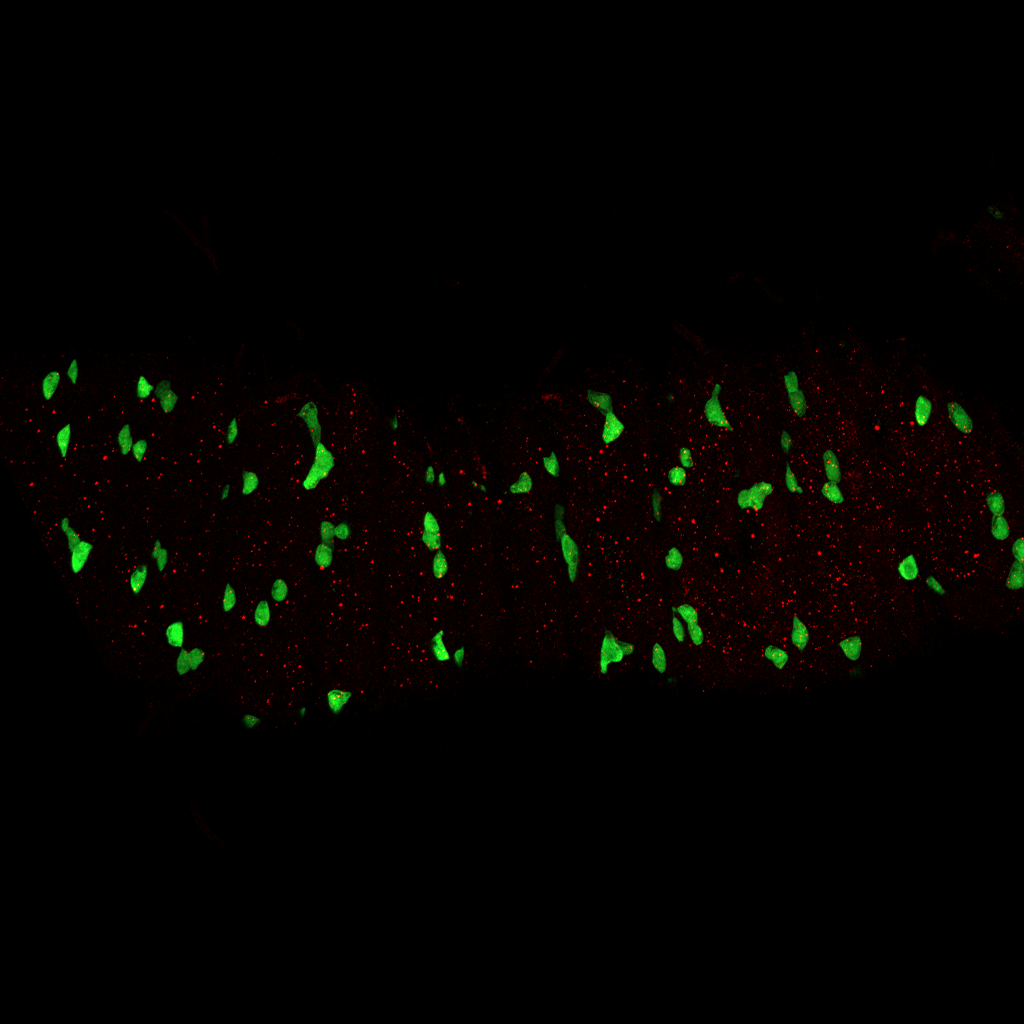

Supplement: Supplementary file 14 — Figure EV4 Source Data [file 44319_2026_701_MOESM14_ESM.zip › EV4/Fig. EV4D-D'/esgts-dwdr4RNAi;CataGFP+pJNK_c1-2.tif]

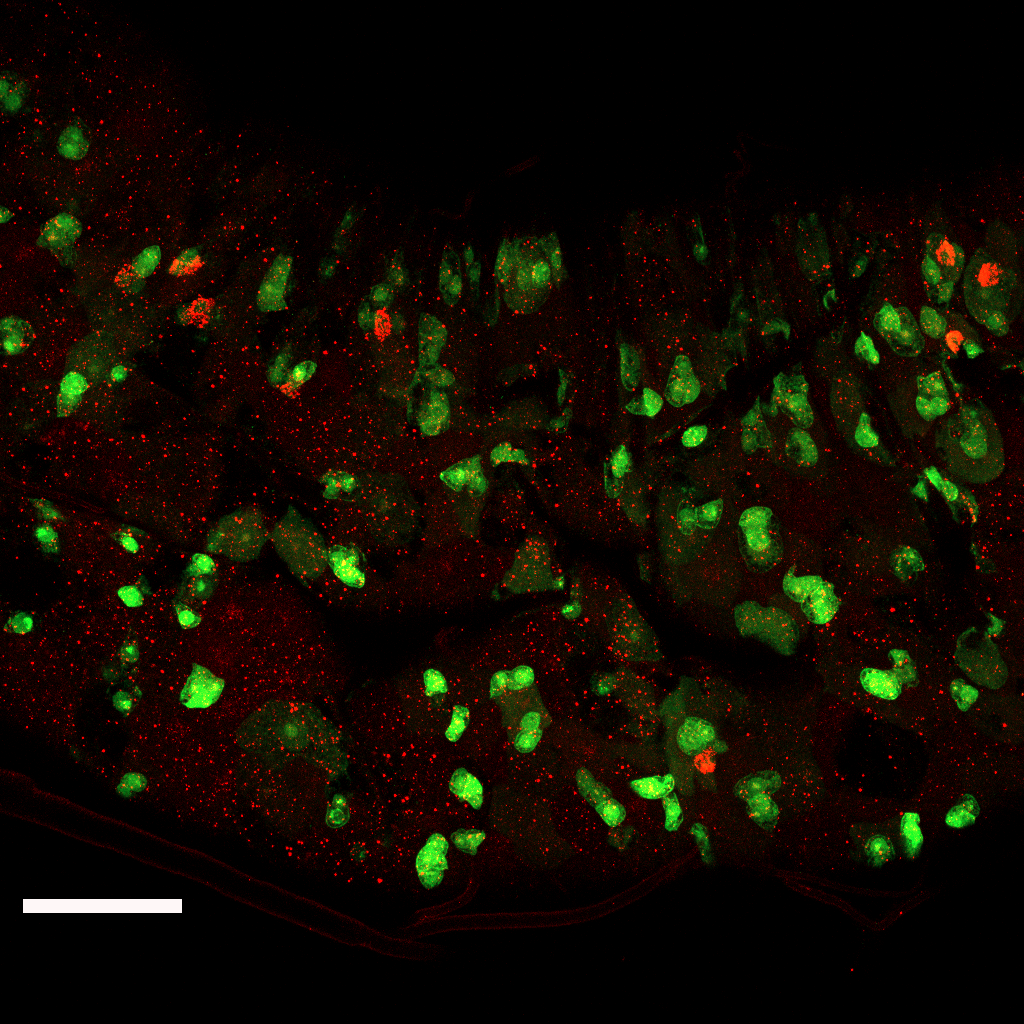

Supplement: Supplementary file 14 — Figure EV4 Source Data [file 44319_2026_701_MOESM14_ESM.zip › EV4/Fig. EV4D-D'/esgts-dwdr4RNAi_GFP+pJNK.tif]

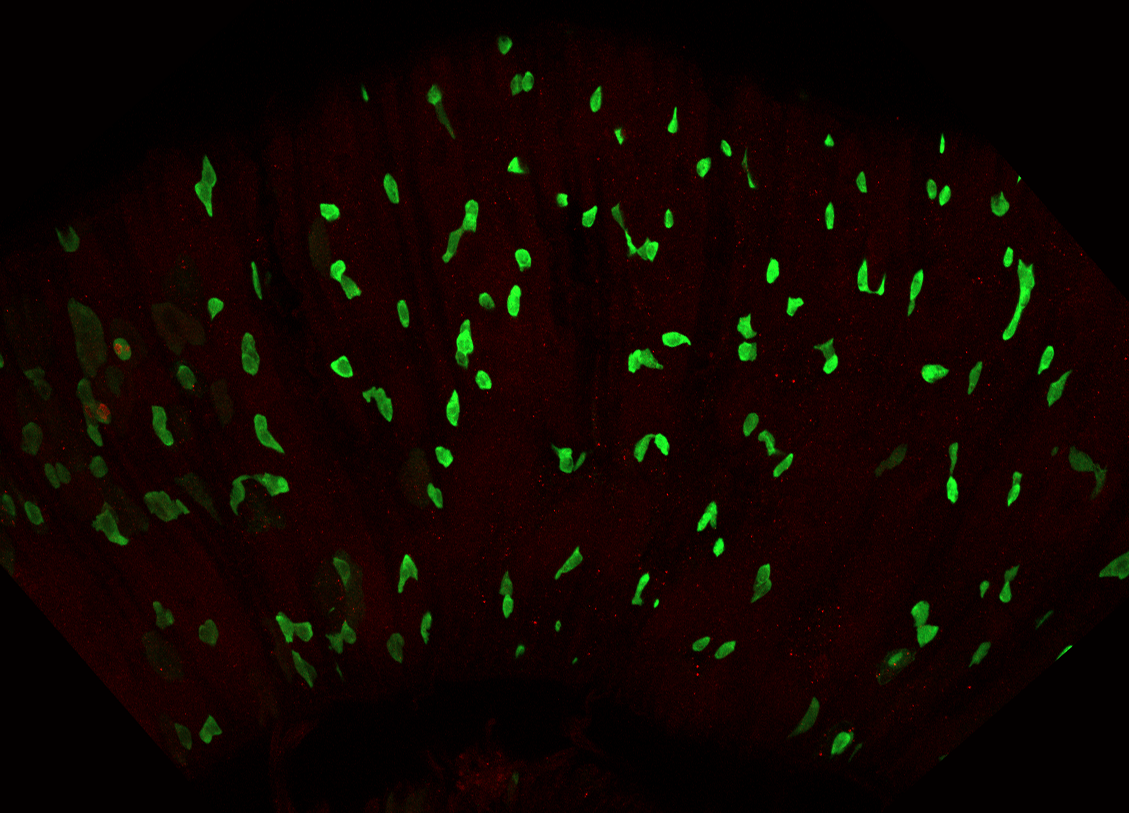

Supplement: Supplementary file 14 — Figure EV4 Source Data [file 44319_2026_701_MOESM14_ESM.zip › EV4/Fig. EV4E-E'/esgts-dwdr4RNAi;Catalase_GFP+phh3.tif]

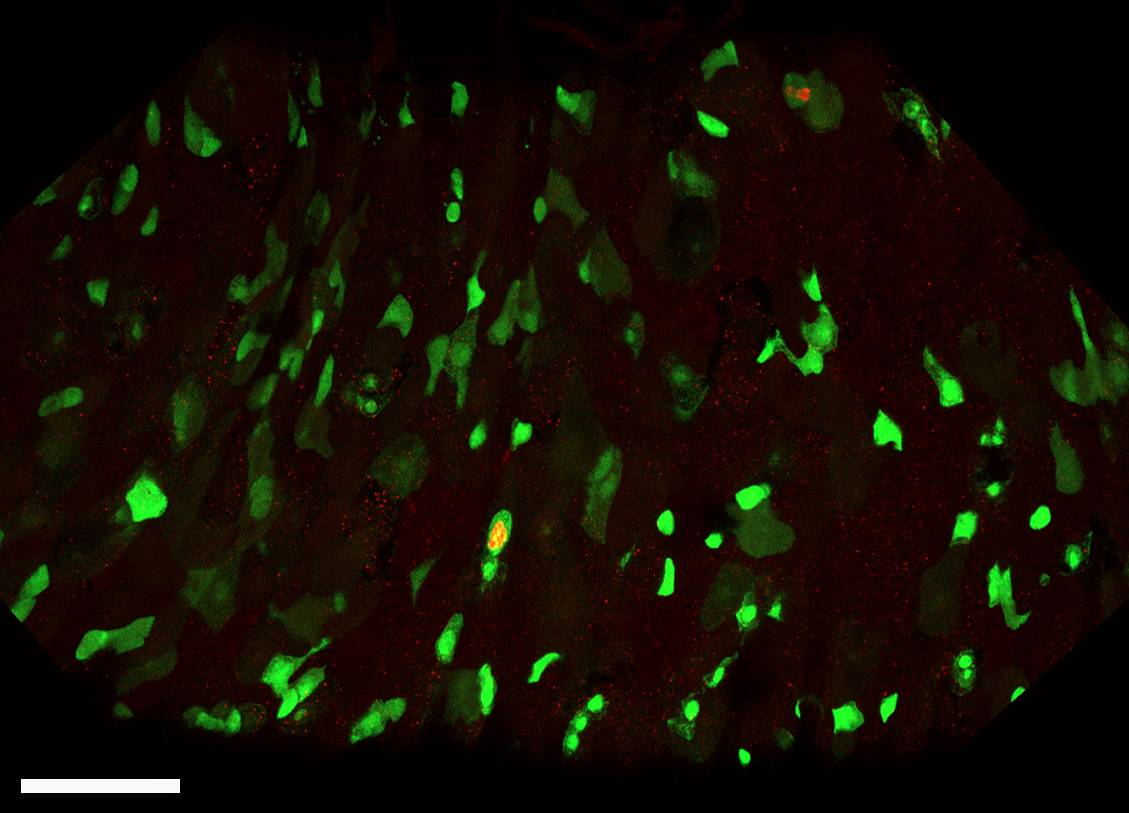

Supplement: Supplementary file 14 — Figure EV4 Source Data [file 44319_2026_701_MOESM14_ESM.zip › EV4/Fig. EV4E-E'/esgts-dwdr4RNAi_GFP+phh3.tif]

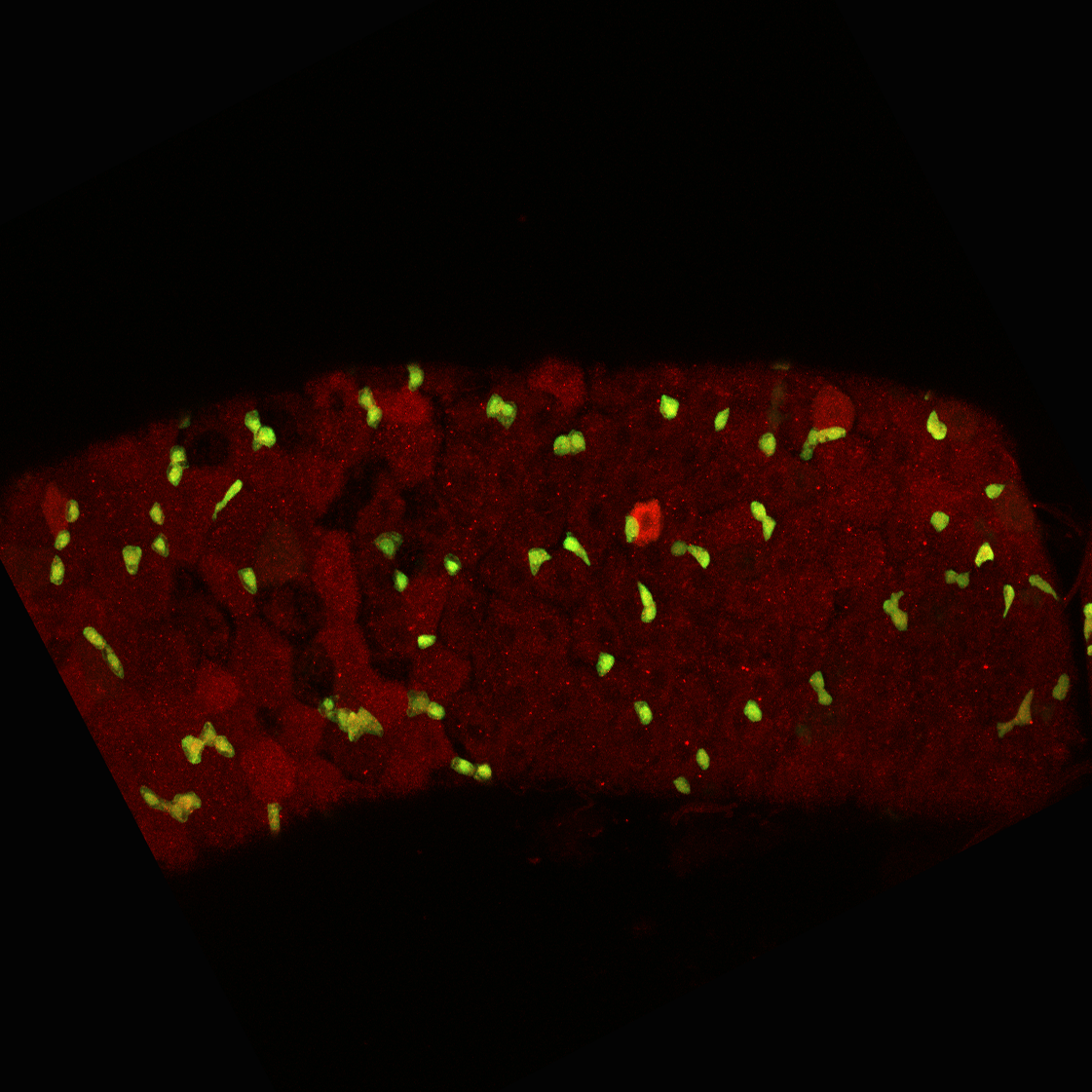

Supplement: Supplementary file 14 — Figure EV4 Source Data [file 44319_2026_701_MOESM14_ESM.zip › EV4/Fig. EV4F-F'/dmyc4;esgts-dwdr4RNAi_GFP+p4ebp.tif]

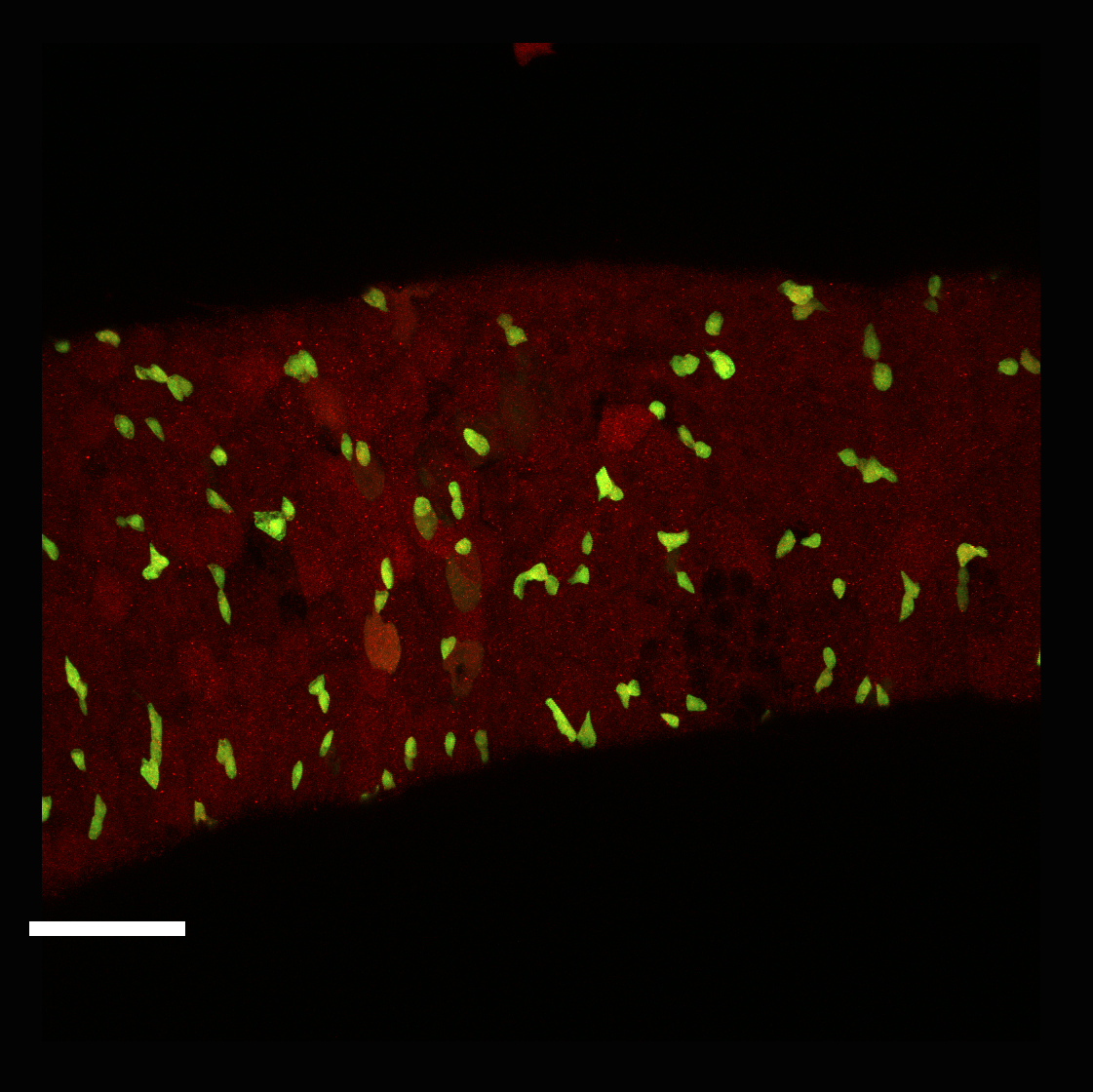

Supplement: Supplementary file 14 — Figure EV4 Source Data [file 44319_2026_701_MOESM14_ESM.zip › EV4/Fig. EV4F-F'/esgts-dwdr4RNAi_GFP+p4ebp.tif]

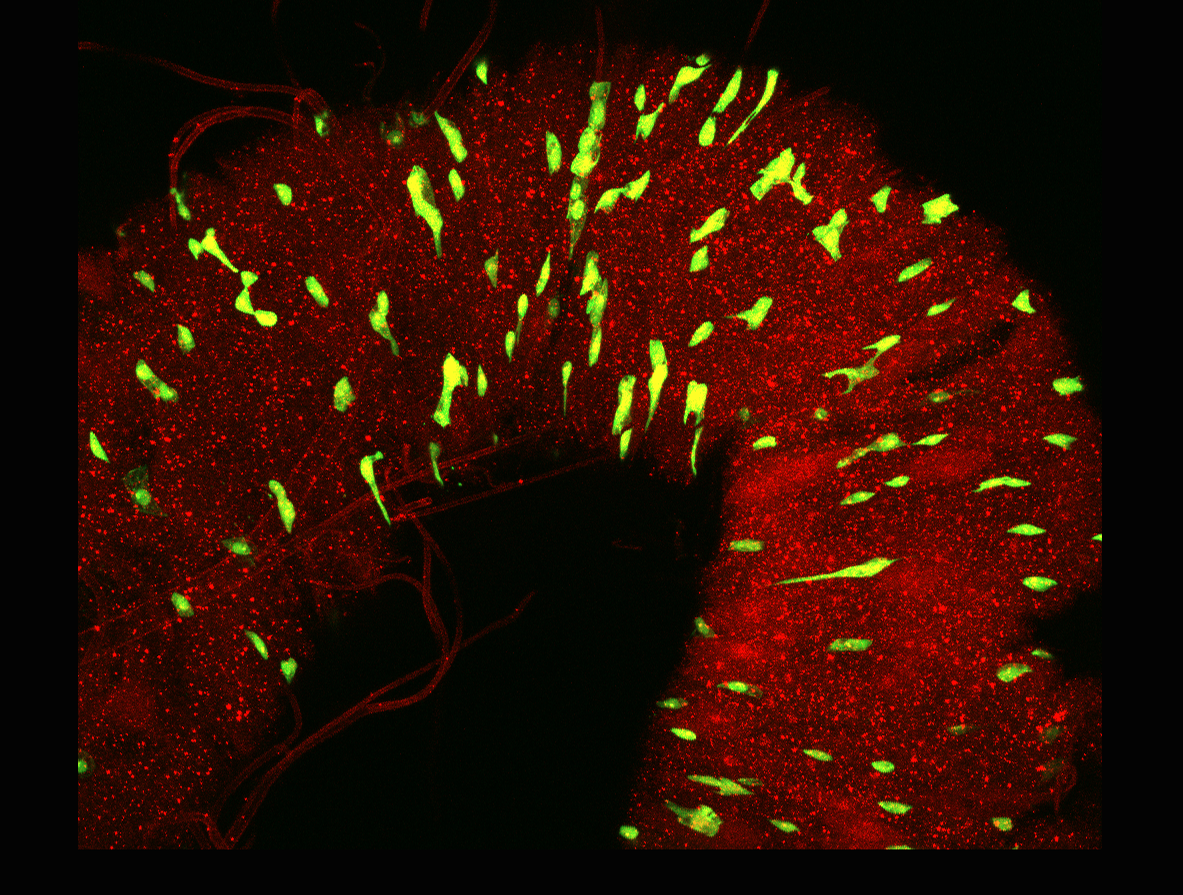

Supplement: Supplementary file 14 — Figure EV4 Source Data [file 44319_2026_701_MOESM14_ESM.zip › EV4/Fig. EV4G-G'/dmyc4;esgts-dwdr4RNAi_GFP+pJNK.tif]

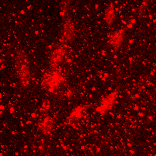

Supplement: Supplementary file 14 — Figure EV4 Source Data [file 44319_2026_701_MOESM14_ESM.zip › EV4/Fig. EV4G-G'/dmyc4;esgts-dwdr4RNAi_pJNK cut.tif]

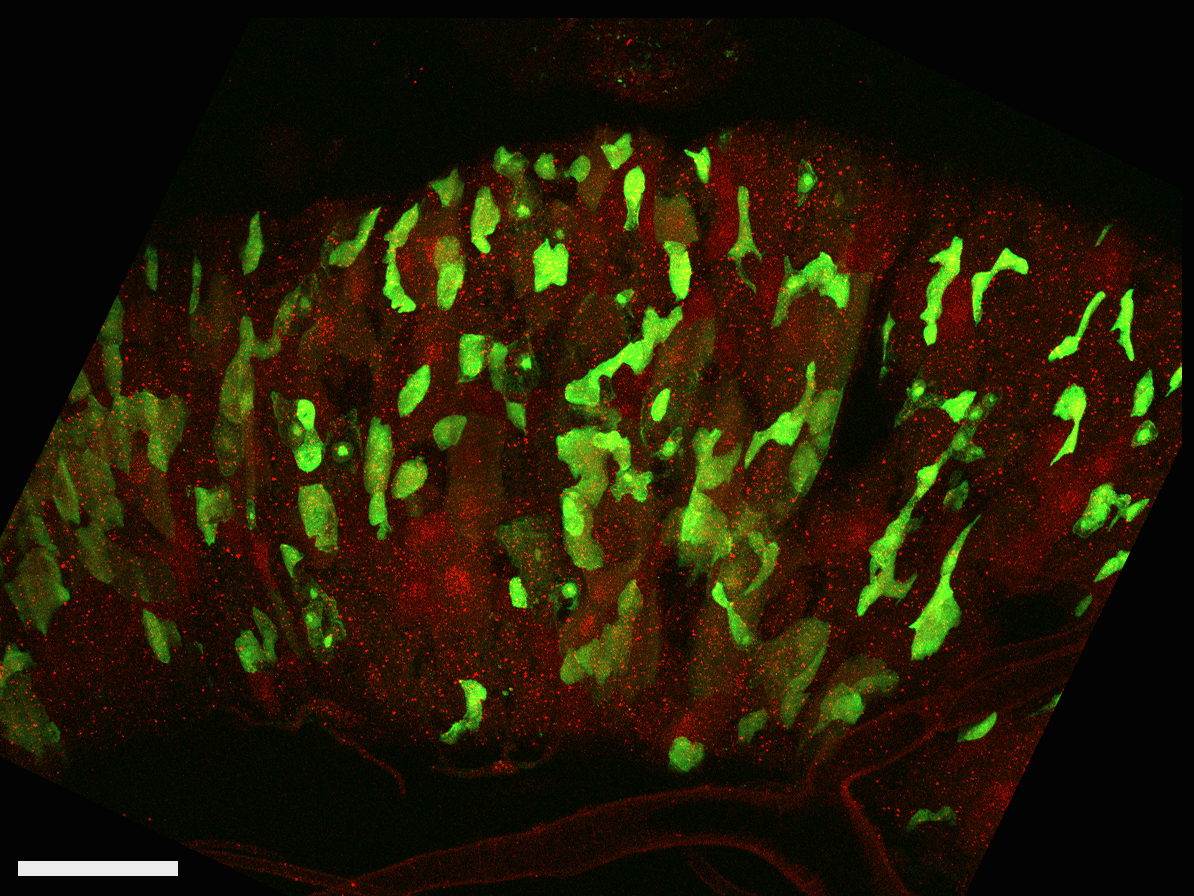

Supplement: Supplementary file 14 — Figure EV4 Source Data [file 44319_2026_701_MOESM14_ESM.zip › EV4/Fig. EV4G-G'/esgts-dwdr4RNAi_GFP+pJNK.tif]

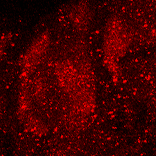

Supplement: Supplementary file 14 — Figure EV4 Source Data [file 44319_2026_701_MOESM14_ESM.zip › EV4/Fig. EV4G-G'/esgts-dwdr4RNAi_pJNK_cut.tif]

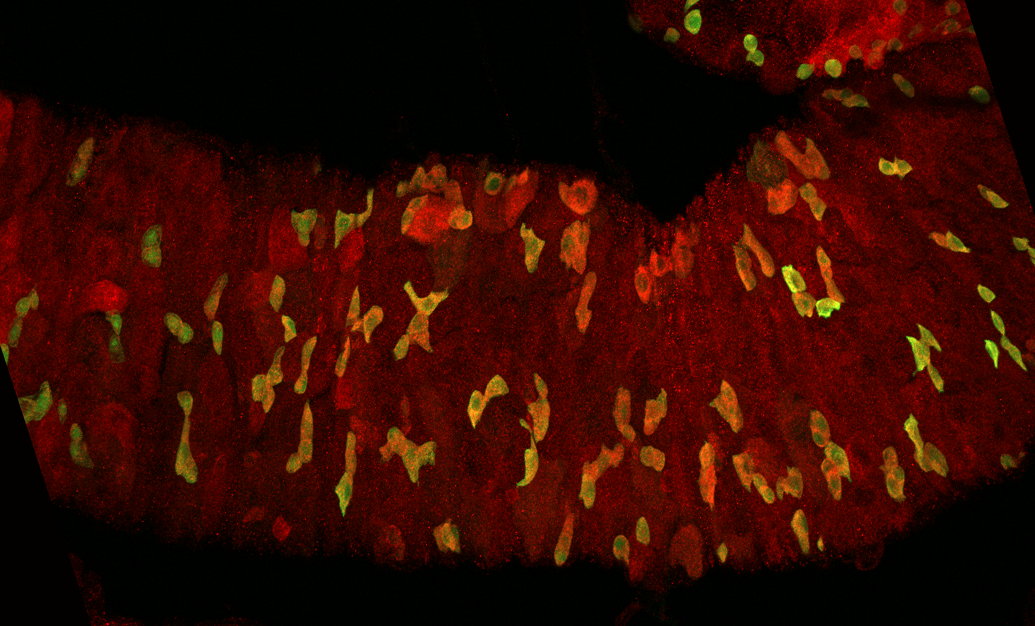

Supplement: Supplementary file 15 — Figure EV5 Source Data [file 44319_2026_701_MOESM15_ESM.zip › EV5/Fig. EV5A-A'/esgts-dwdr4RNAi_GFP+m7G.tif]

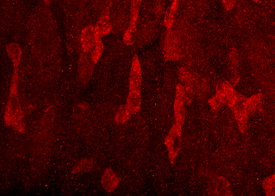

Supplement: Supplementary file 15 — Figure EV5 Source Data [file 44319_2026_701_MOESM15_ESM.zip › EV5/Fig. EV5A-A'/esgts-dwdr4RNAi_m7G_cut.tif]

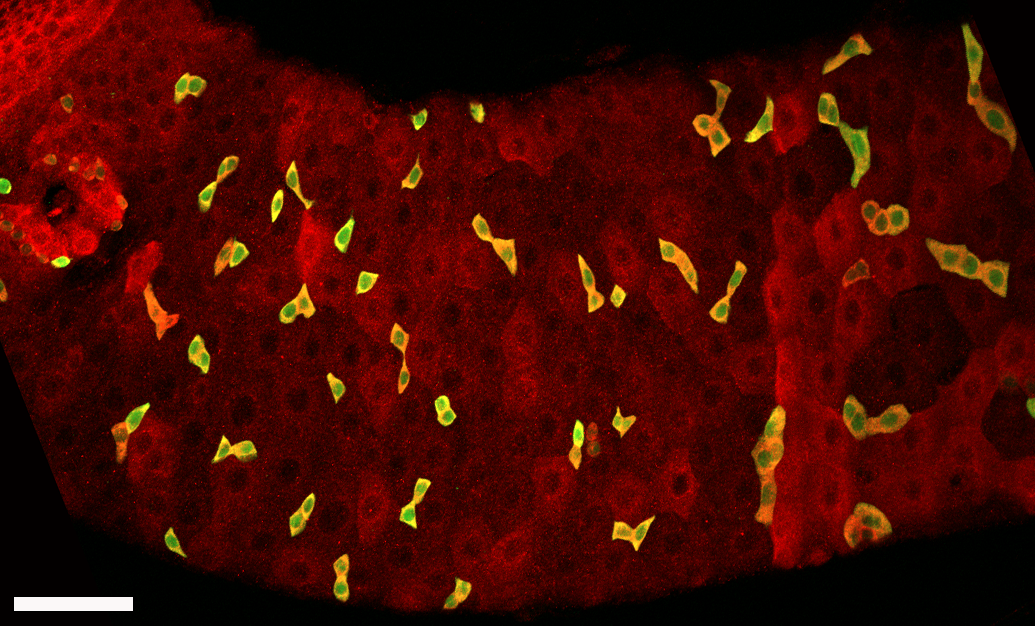

Supplement: Supplementary file 15 — Figure EV5 Source Data [file 44319_2026_701_MOESM15_ESM.zip › EV5/Fig. EV5A-A'/esgts-mcherryRNAi_GFP+m7G.tif]

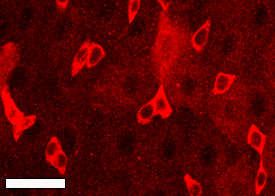

Supplement: Supplementary file 15 — Figure EV5 Source Data [file 44319_2026_701_MOESM15_ESM.zip › EV5/Fig. EV5A-A'/esgts-mcherryRNAi_GFP+m7G_cut.tif]

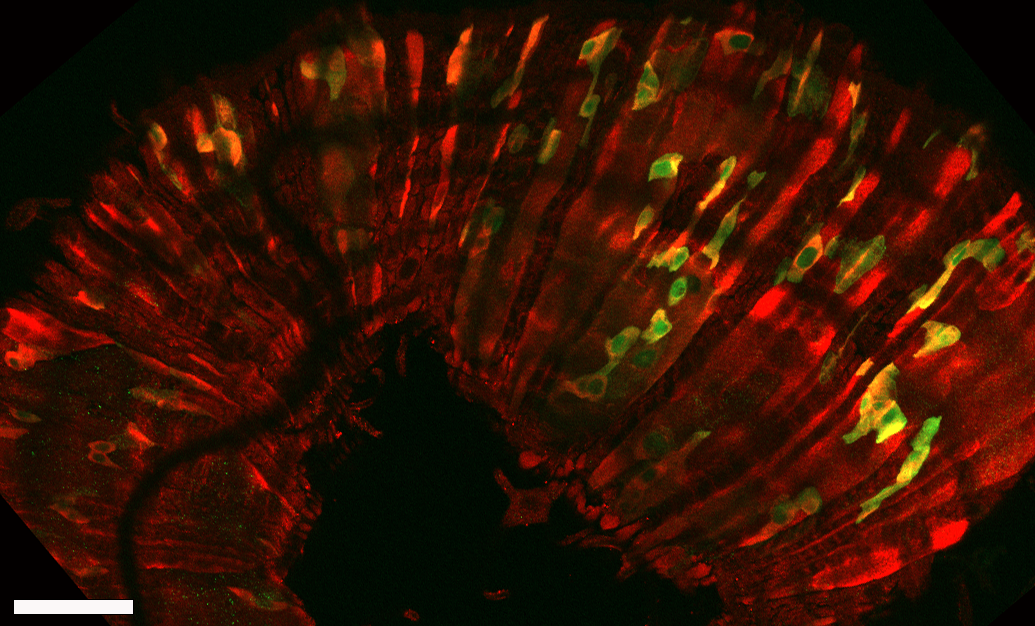

Supplement: Supplementary file 15 — Figure EV5 Source Data [file 44319_2026_701_MOESM15_ESM.zip › EV5/Fig. EV5B-B'/esgts-mcherryRNAi_m7G+GFP.tif]

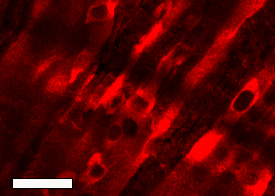

Supplement: Supplementary file 15 — Figure EV5 Source Data [file 44319_2026_701_MOESM15_ESM.zip › EV5/Fig. EV5B-B'/esgts-mcherryRNAi_m7G_cut.tif]

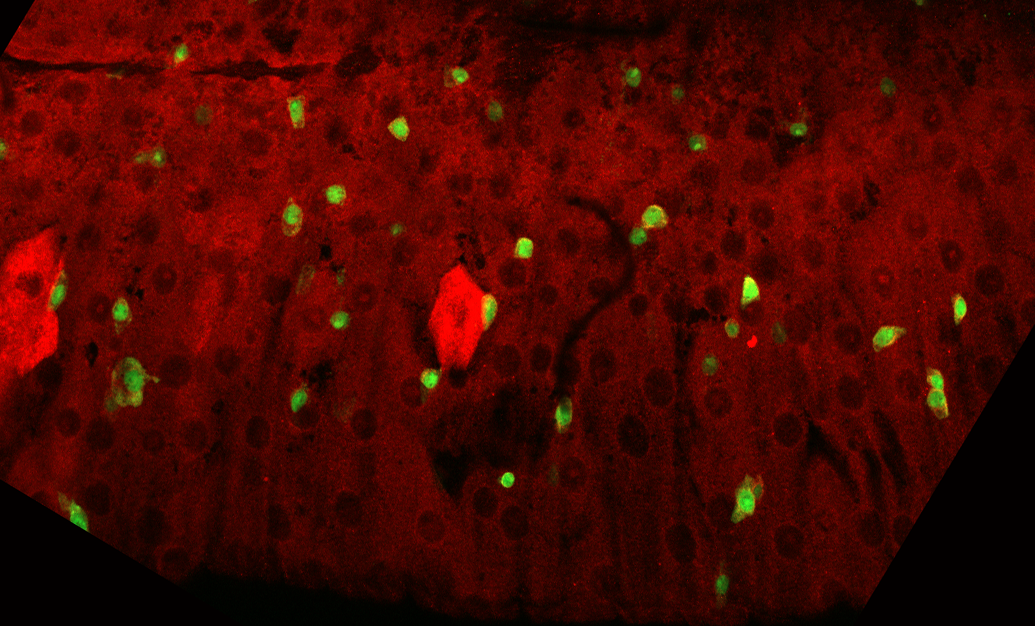

Supplement: Supplementary file 15 — Figure EV5 Source Data [file 44319_2026_701_MOESM15_ESM.zip › EV5/Fig. EV5B-B'/esgts_Mettl1RNAi_GFP+m7G.tif]

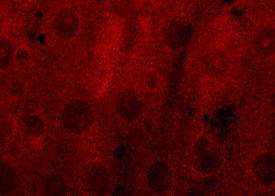

Supplement: Supplementary file 15 — Figure EV5 Source Data [file 44319_2026_701_MOESM15_ESM.zip › EV5/Fig. EV5B-B'/esgts_Mettl1RNAi_m7G_cut.tif]

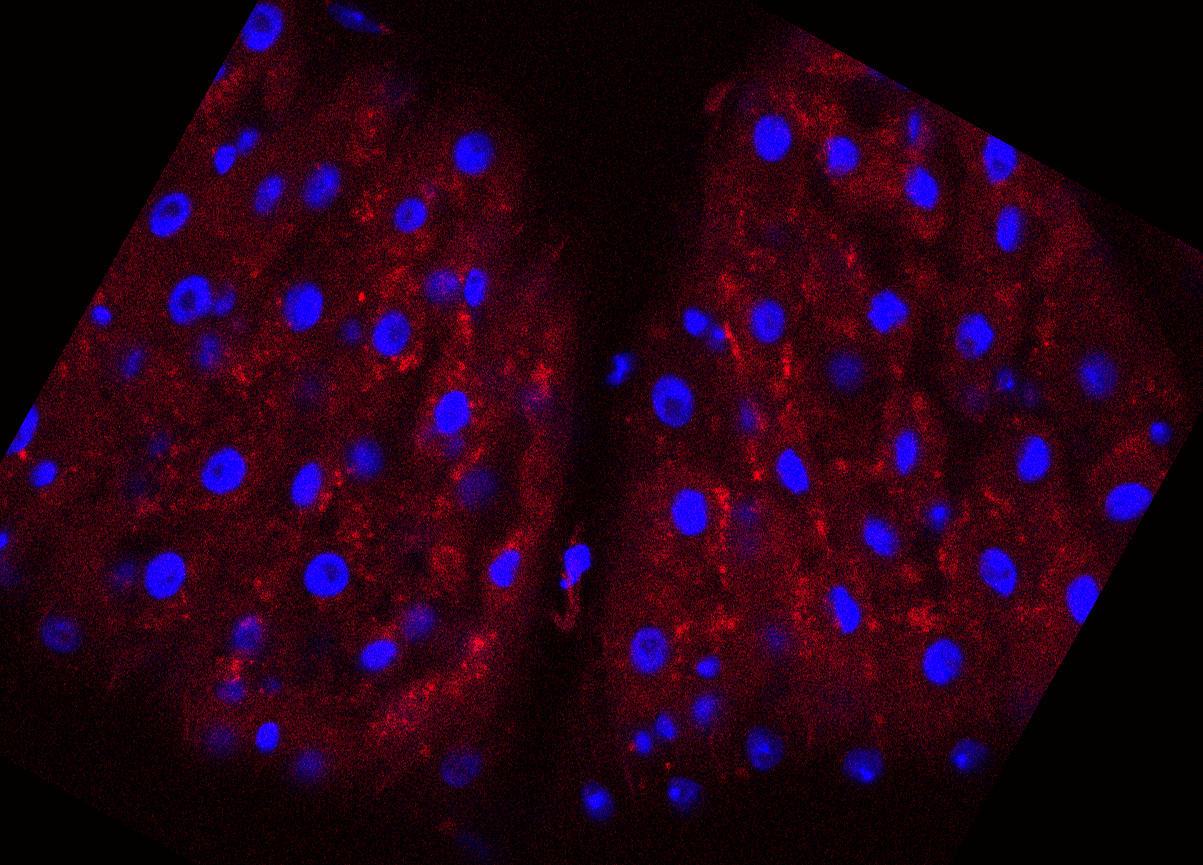

Supplement: Supplementary file 15 — Figure EV5 Source Data [file 44319_2026_701_MOESM15_ESM.zip › EV5/Fig. EV5C-C'/dwdr4-gfp;esg-dmettl1-V5; experimental.tif]

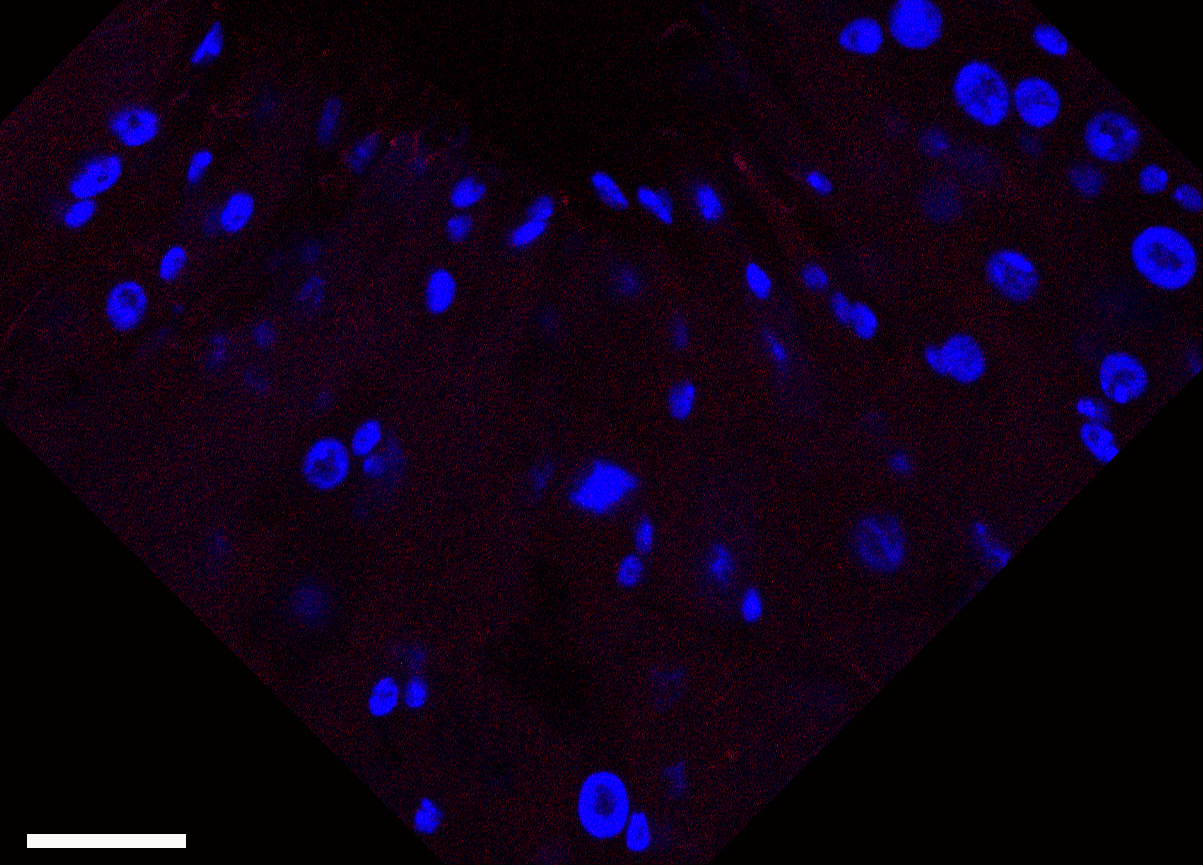

Supplement: Supplementary file 15 — Figure EV5 Source Data [file 44319_2026_701_MOESM15_ESM.zip › EV5/Fig. EV5C-C'/dwdr4-gfp;esg-dmettl1-v5; neg ctrl.tif]

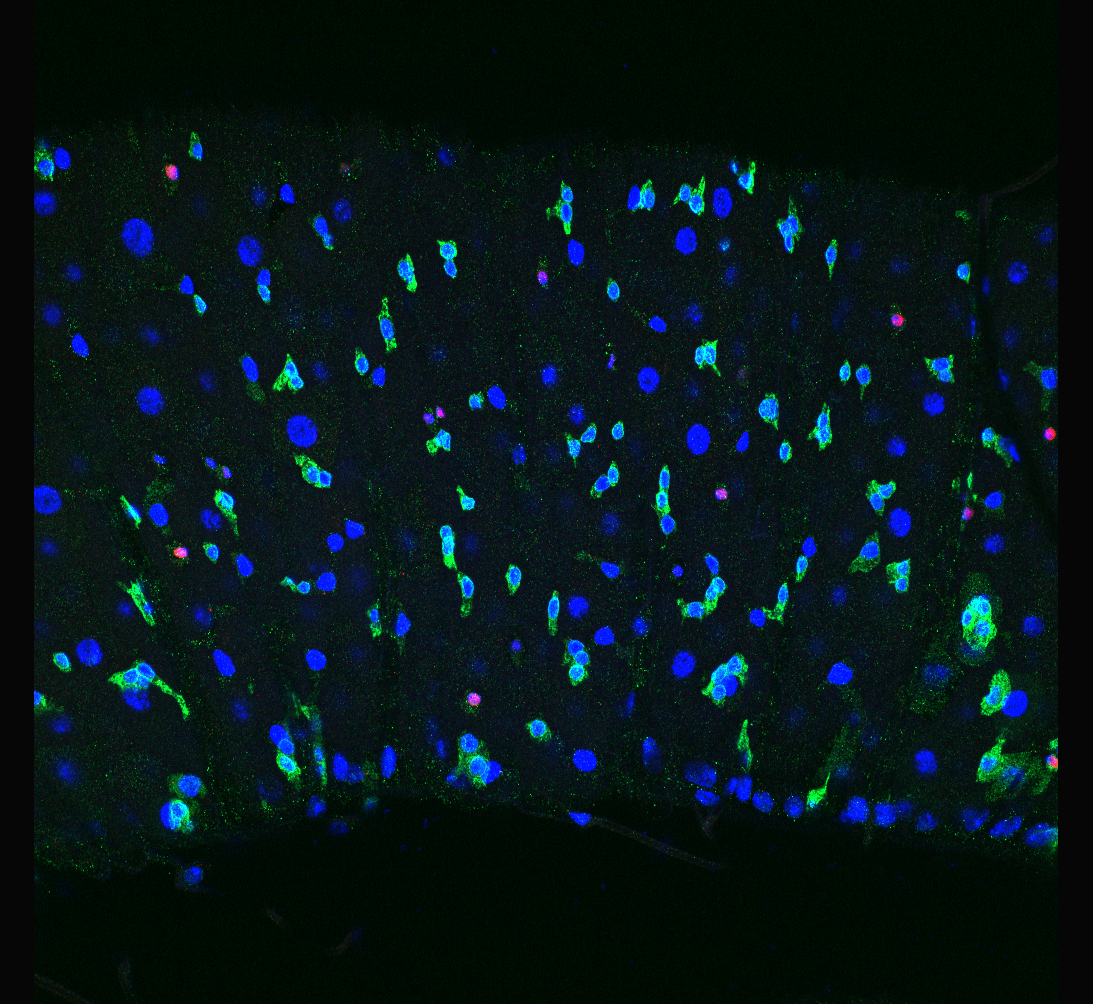

Supplement: Supplementary file 15 — Figure EV5 Source Data [file 44319_2026_701_MOESM15_ESM.zip › EV5/Fig. EV5D-D'/wh7;esg-hWDR4_GFP+Pros+DAPI.tif]

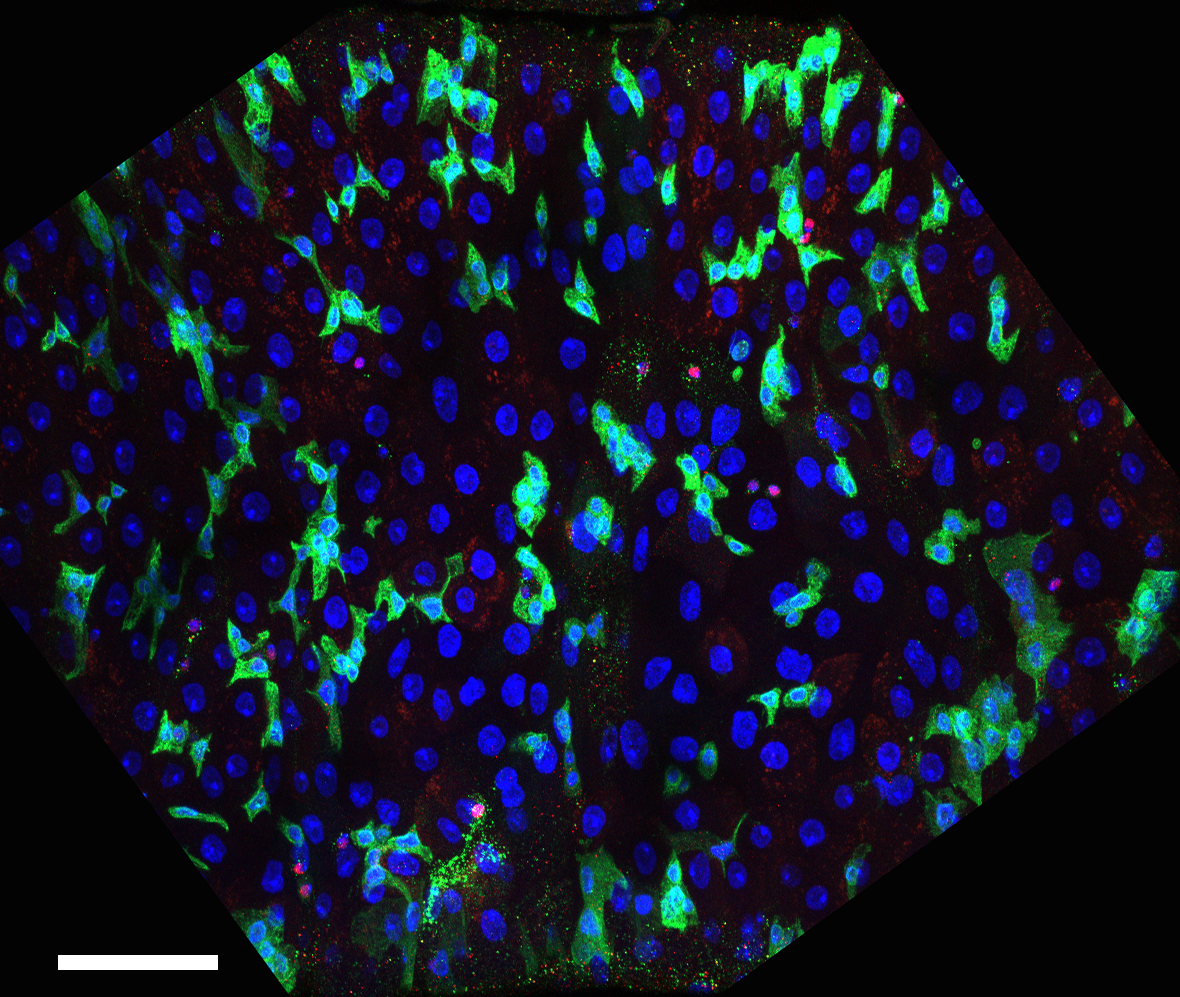

Supplement: Supplementary file 15 — Figure EV5 Source Data [file 44319_2026_701_MOESM15_ESM.zip › EV5/Fig. EV5D-D'/wh7;esg-mcdGFP_GFP+Pros+DAPI.tif]

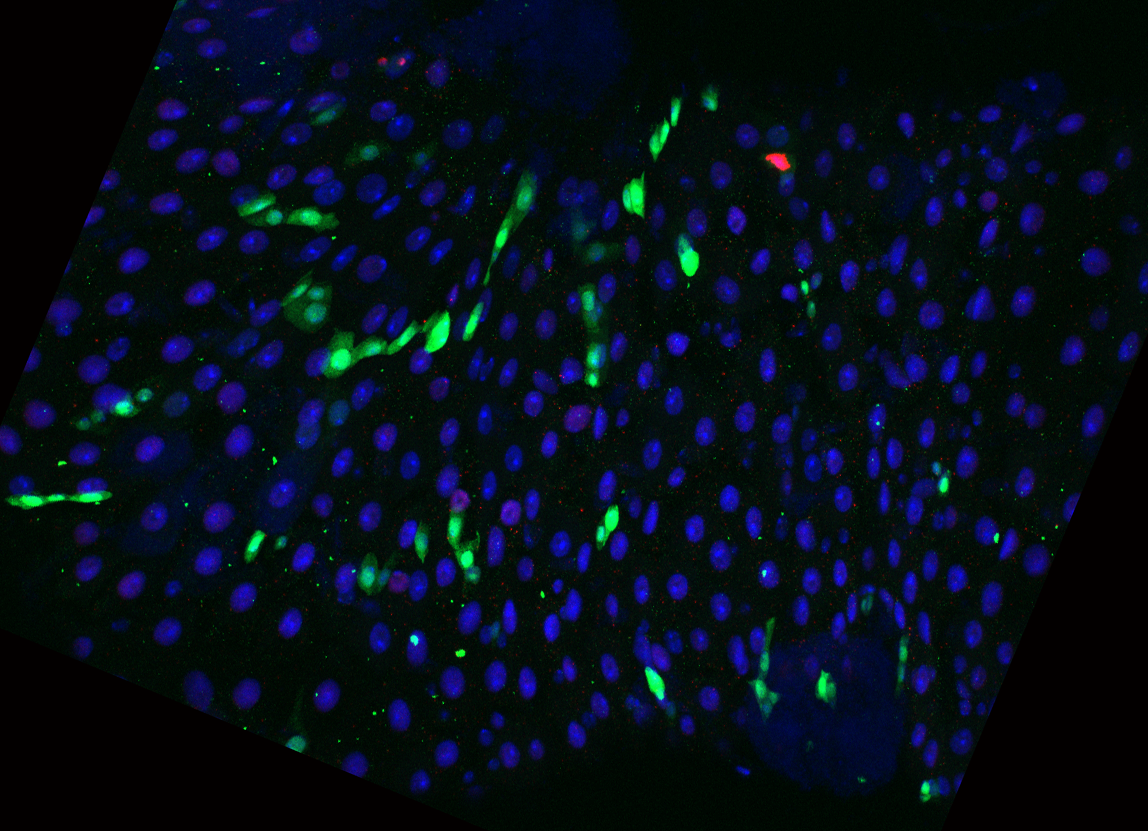

Supplement: Supplementary file 15 — Figure EV5 Source Data [file 44319_2026_701_MOESM15_ESM.zip › EV5/Fig. EV5F-F'/Dlts-dmeetl1RNAi;hMETTL1_gfp+phh3+DAPI.tif]

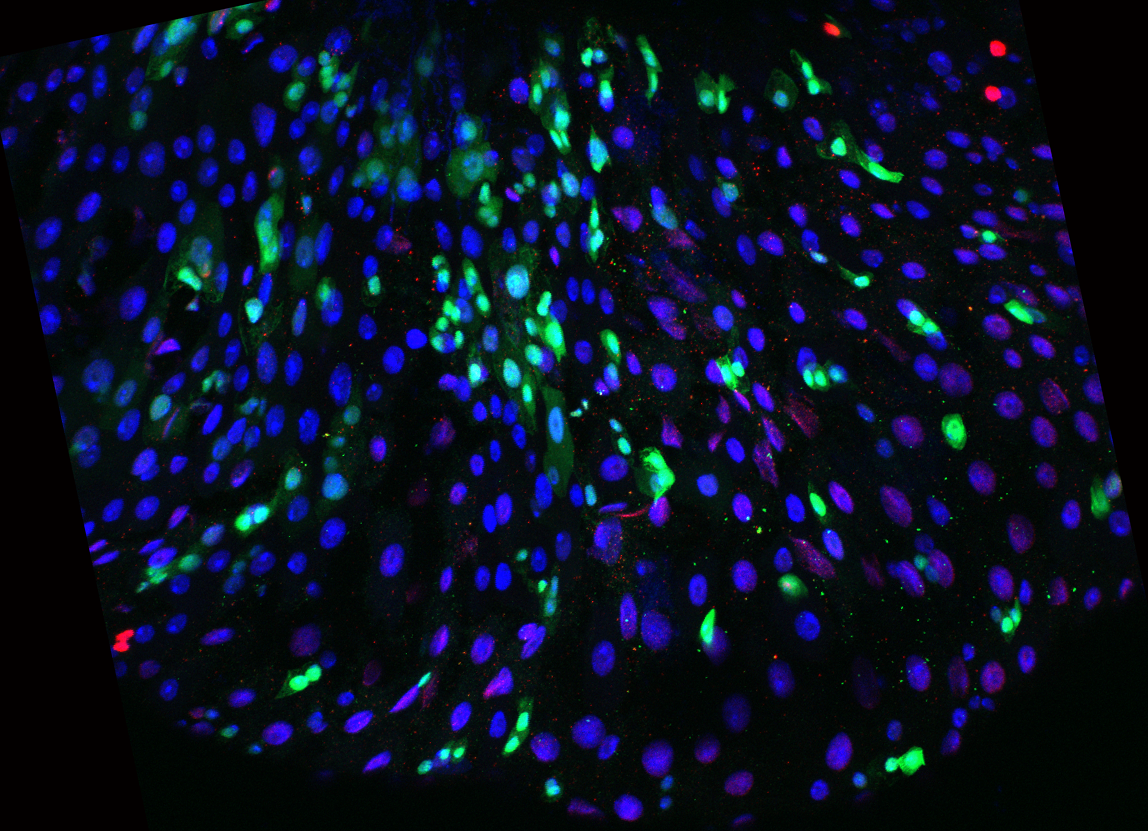

Supplement: Supplementary file 15 — Figure EV5 Source Data [file 44319_2026_701_MOESM15_ESM.zip › EV5/Fig. EV5F-F'/Dlts-dmettl1RNAi;hMETTL1CD_GFP+phh3+DAPI.tif]

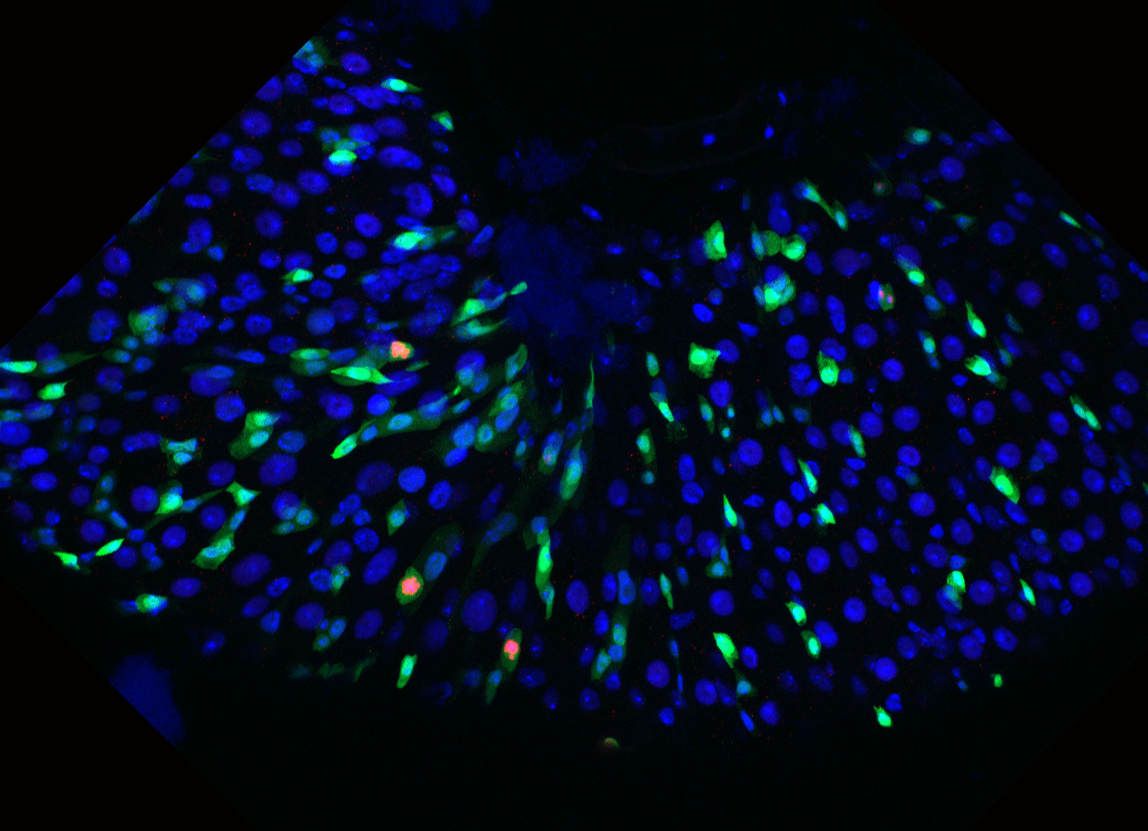

Supplement: Supplementary file 15 — Figure EV5 Source Data [file 44319_2026_701_MOESM15_ESM.zip › EV5/Fig. EV5F-F'/Dlts-dmettl1RNAi_gfp+phh3+DAPI.tif]

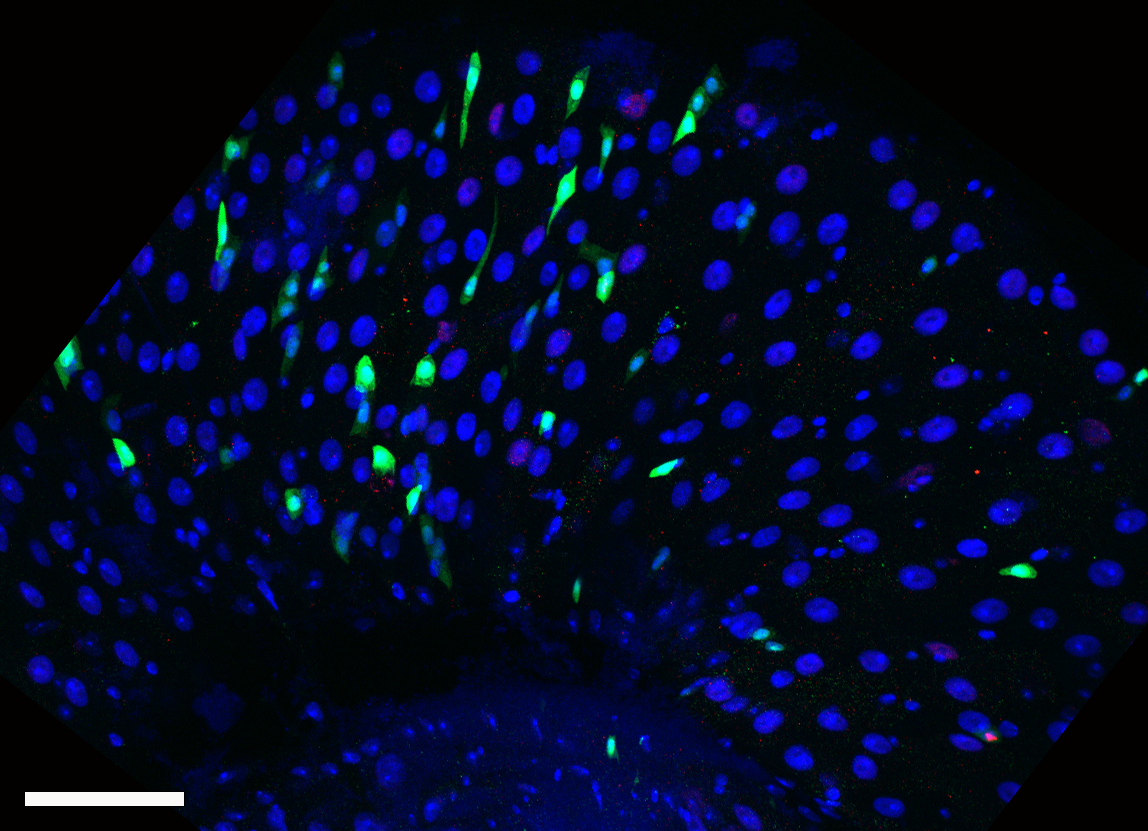

Supplement: Supplementary file 15 — Figure EV5 Source Data [file 44319_2026_701_MOESM15_ESM.zip › EV5/Fig. EV5F-F'/Dlts-mcherryRNAi_gfp+phh3+DAPI.tif]

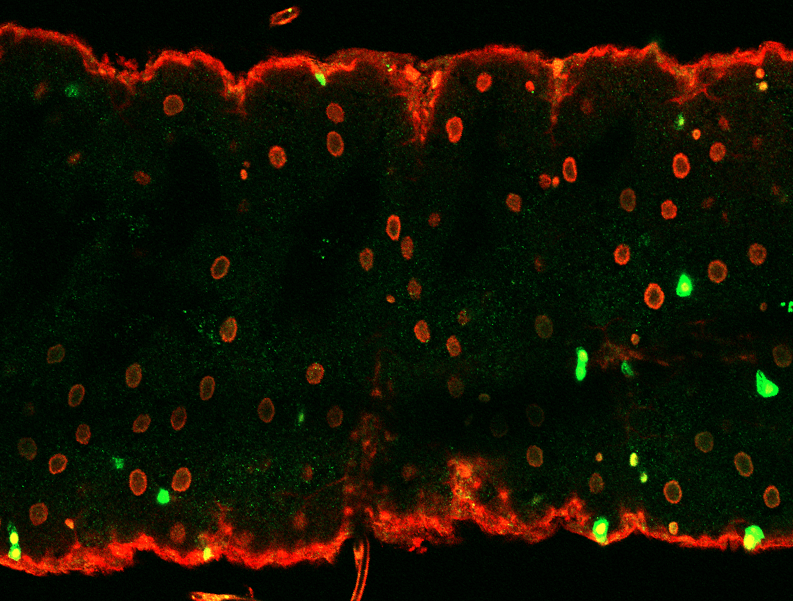

Supplement: Supplementary file 16 — Figure EV6 Source Data [file 44319_2026_701_MOESM16_ESM.zip › EV6/Fig. EV6B/Dlts-dwdr4RNAi_lacZ+GFP.tif]

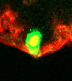

Supplement: Supplementary file 16 — Figure EV6 Source Data [file 44319_2026_701_MOESM16_ESM.zip › EV6/Fig. EV6B/Dlts-dwdr4RNAi_lacZ+GFP_cut.tif]

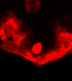

Supplement: Supplementary file 16 — Figure EV6 Source Data [file 44319_2026_701_MOESM16_ESM.zip › EV6/Fig. EV6B/Dlts-dwdr4RNAi_lacZ_cut.tif]

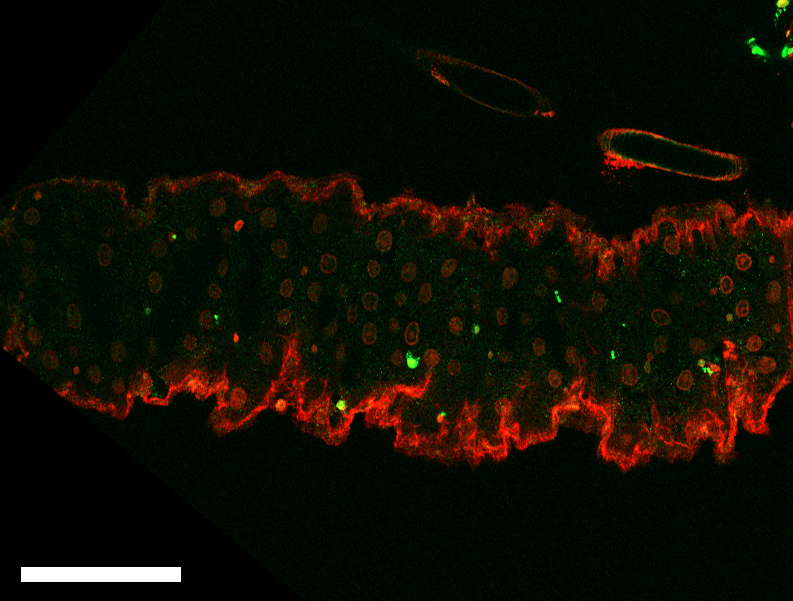

Supplement: Supplementary file 16 — Figure EV6 Source Data [file 44319_2026_701_MOESM16_ESM.zip › EV6/Fig. EV6B/Dlts-mcherryRNAi_GFP+lacZ.tif]

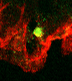

Supplement: Supplementary file 16 — Figure EV6 Source Data [file 44319_2026_701_MOESM16_ESM.zip › EV6/Fig. EV6B/Dlts-mcherryRNAi_GFP+lacZ_cut.tif]

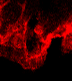

Supplement: Supplementary file 16 — Figure EV6 Source Data [file 44319_2026_701_MOESM16_ESM.zip › EV6/Fig. EV6B/Dlts-mcherryRNAi_lacZ_cut.tif]

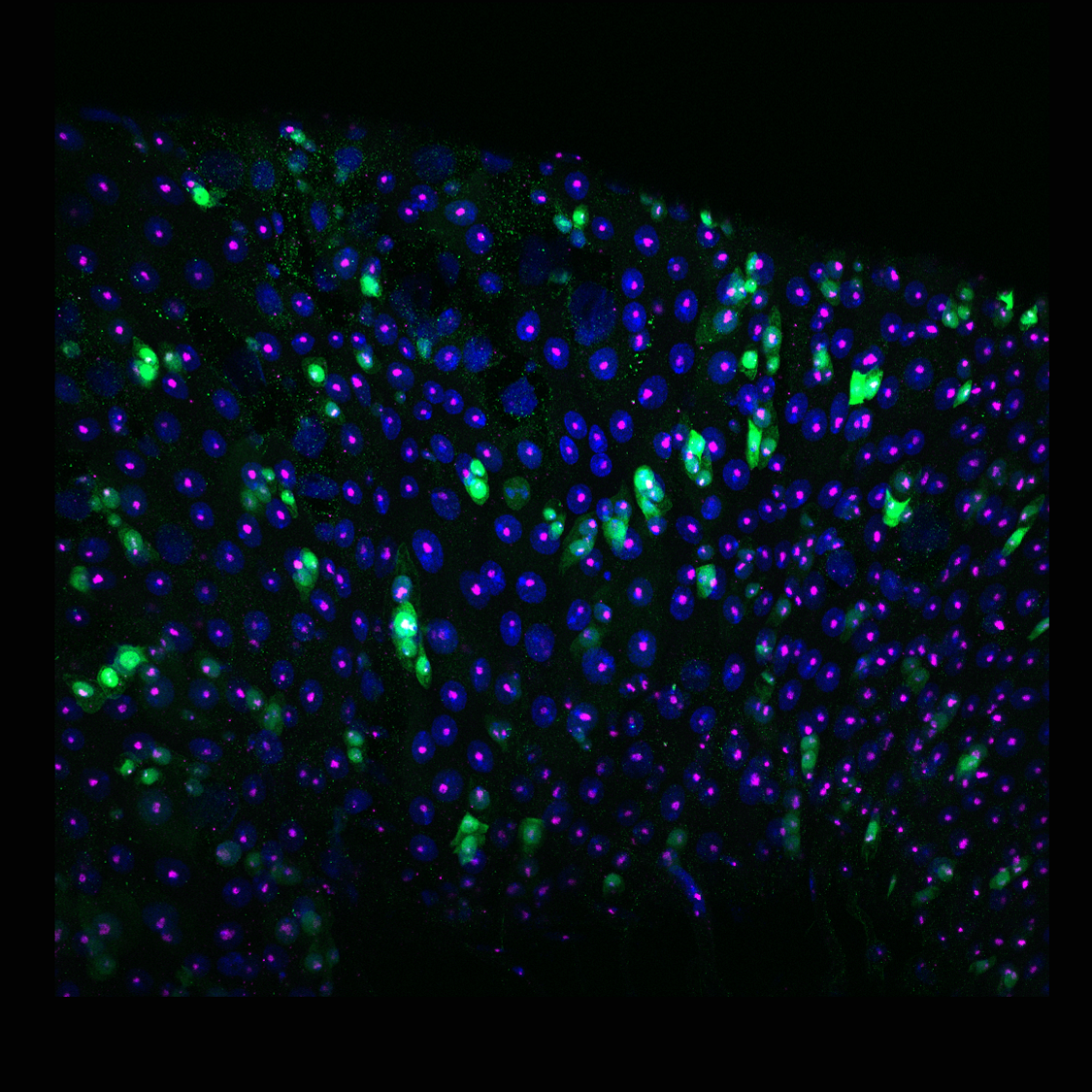

Supplement: Supplementary file 16 — Figure EV6 Source Data [file 44319_2026_701_MOESM16_ESM.zip › EV6/Fig. EV6C-C'/Dlts-let7-decoy_GFP+Fibrillarin+DAPI.tif]

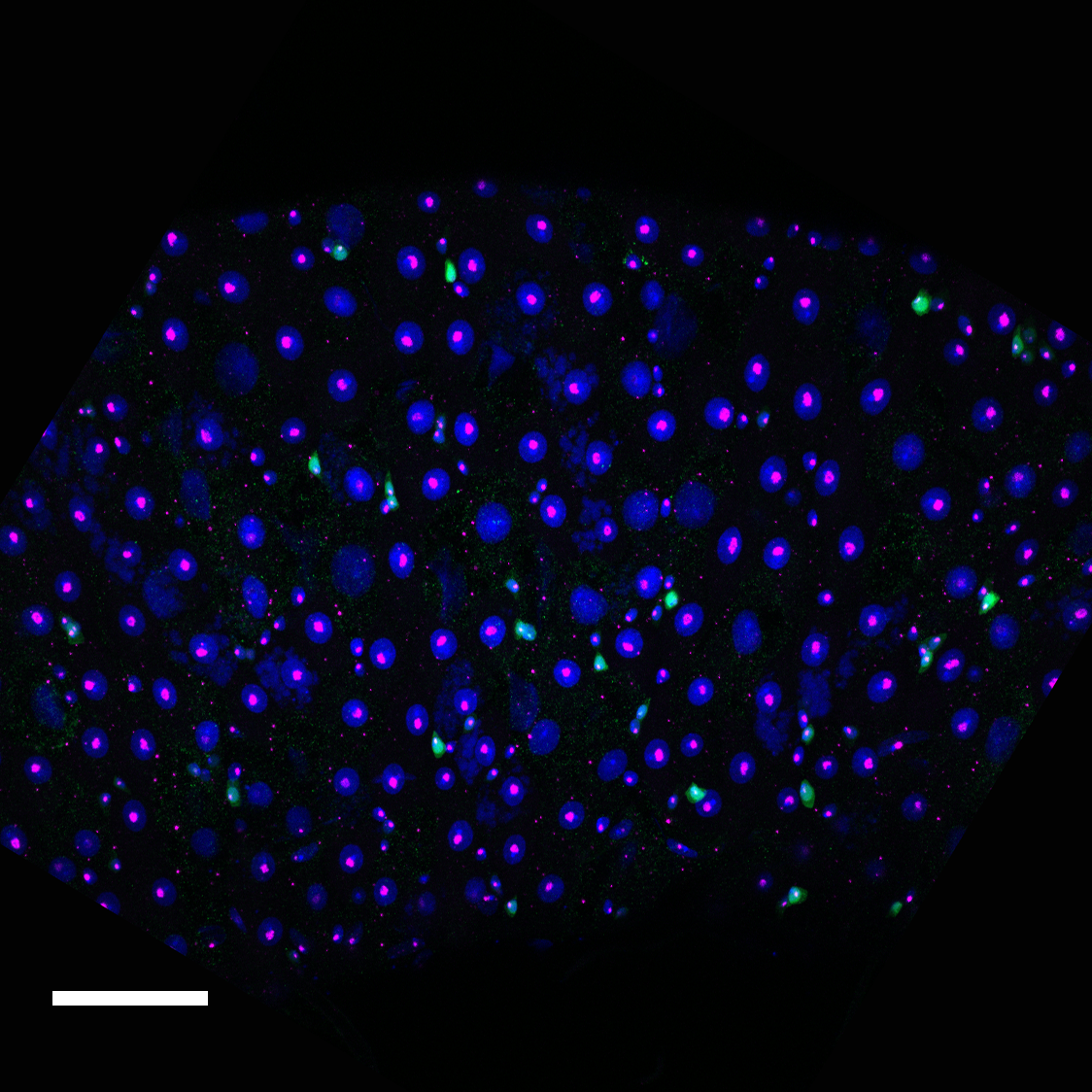

Supplement: Supplementary file 16 — Figure EV6 Source Data [file 44319_2026_701_MOESM16_ESM.zip › EV6/Fig. EV6C-C'/Dlts-mcherryRNAi_GFP+Fibrillarin+DAPI.tif]
