# Supplementary material for: Wdr4 regulates ribosome biogenesis and intestinal homeostasis via let-7
Source: EMBO Rep. 2026 Feb 9;27(8):1870–903. doi: 10.1038/s44319-026-00701-y (PMC13121520; doi:10.1038/s44319-026-00701-y)
Supplement: Supplementary file 17 — Expanded View Figures [file 44319_2026_701_MOESM17_ESM.pdf]

Expanded View Figures

Figure EV1. Loss of dWdr4 shortens lifespan, impairs locomotor activity, and disrupts gut function in flies.

(**A**, **B**) *Drosophila dwdr4* mutant (*wh*^{7}) female (**A**) and male flies (**B**) show shortened lifespans compared to wild-type (WT) controls. Lifespan defects were rescued by introducing a copy of *dwdr4-gfp* ($N = 80$ flies). (**C**, **D**) Climbing assays reveal reduced locomotor activity in *wh*^{7} mutant female (**C**) and male flies (**D**) compared to WT control. Climbing performance was partially improved in females ($N = 17$ flies) and completely rescued in males ($N = 15$ flies) by *dwdr4-gfp* expression. (**E**) dWdr4 loss causes the accumulation of lipid droplets. WT ($N = 7$) and *wh7* mutant ($N = 9$) guts with Bodipy (green, oil droplets), Phalloidin (magenta, F-actin), and DAPI (blue, nuclei). The fraction of gut sections with the pattern shown is indicated. (**F**) Expression differences in genes involved in lipid metabolism and gut digestion between the *wh*^{7} and WT female guts. Fold change of $\log_2$ values ($P < 0.05$) is indicated by the color indicator. Each dot in (**C**, **D**) represents one fly. Scale bar in (**E**) is 50 µm. Bars represent the mean; error bars indicate standard deviation (SD). Statistically significant differences in (**A**, **B**) were identified by the Log-rank test, and in (**C**, **D**) were identified by the unpaired Student's *t* test. Statistical analysis in (**F**) was performed on two biological replicates. Lifespan analysis in (**A**, **B**) showed significant differences between WT and *wh*^{7}, *wh*^{7} and *wh*^{7}*;dwdr4-gfp*, and between WT and *wh*^{7}*;dwdr4-gfp* (***$P = < 0.0001$). Climbing assay in (**C**, **D**) showed significant differences between WT and *wh*^{7} (***$P = < 0.0001$), *wh*^{7} and *wh*^{7}*;dwdr4-gfp* (**$P = 0.0014$), and between WT and *wh*^{7}*;dwdr4-gfp* (***$P = 0.0004$) in females, but in males, no differences were observed between WT and *wh*^{7}*;dwdr4-gfp* (ns, $P = 0.621$). *N*, number of midguts, unless otherwise stated. Source data are available online for this figure.

 © The Author(s)

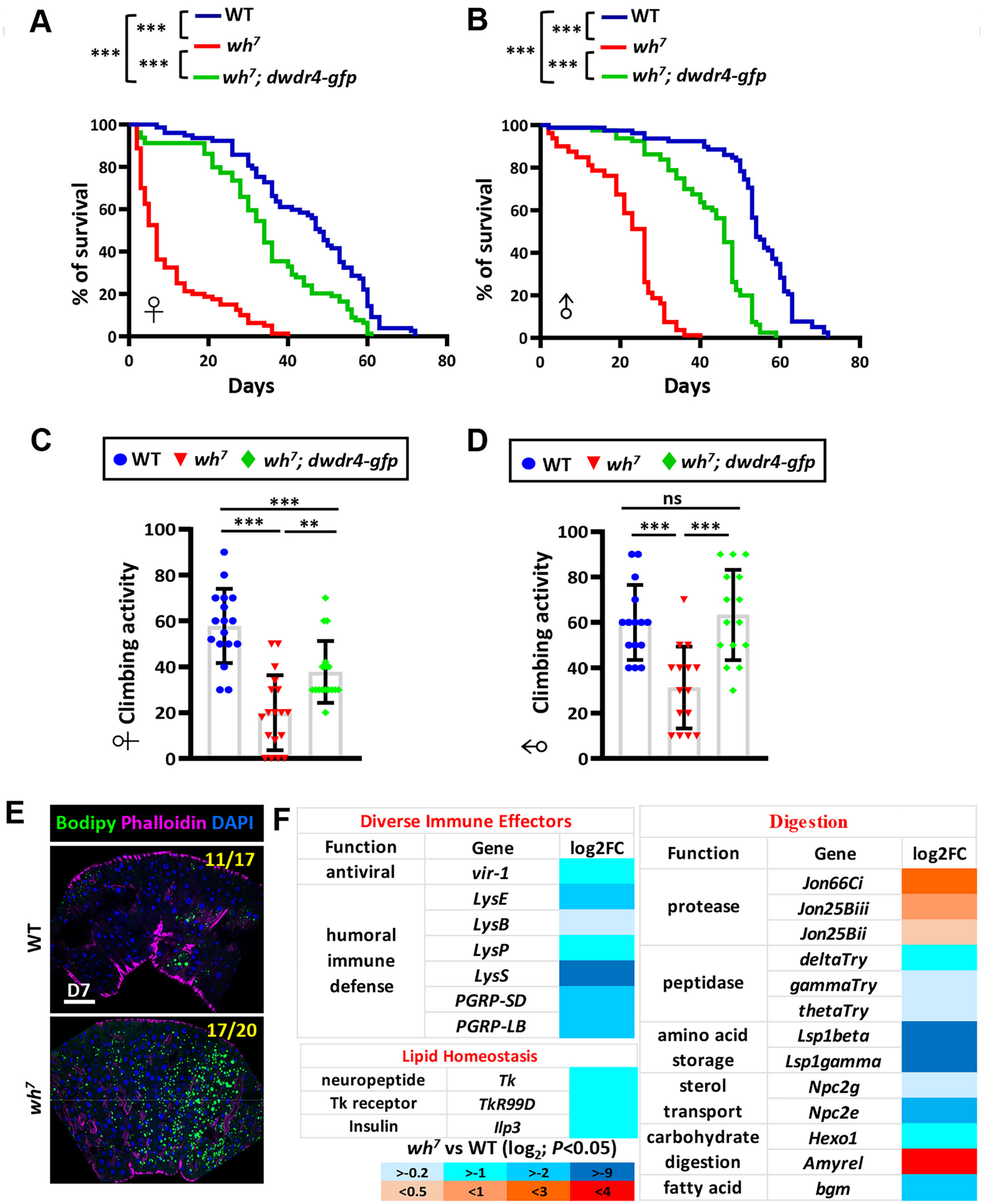
A
WT
wh7
wh7; dwdr4-gfp
***
% of survival
Days
B
WT
wh7
wh7; dwdr4-gfp
***
% of survival
Days
C
WT wh7 wh7; dwdr4-gfp
***
**
Climbing activity
D
WT wh7 wh7; dwdr4-gfp
ns
***
Climbing activity
E
Bodipy Phalloidin DAPI
11/17
WT
D7
17/20
wh7
F
Diverse Immune Effectors
Function Gene log2FC
antiviral vir-1
humoral immune defense LysE LysB LysP LysS PGRP-SD PGRP-LB
Lipid Homeostasis
neuropeptide Tk
Tk receptor TkR99D
Insulin Ilp3
wh7 vs WT (log2; P<0.05)
>-0.2 >-1 >-2 >-9
<0.5 <1 <3 <4
Digestion
Function Gene log2FC
protease Jon66Ci Jon25Biii Jon25Bii
peptidase deltaTry gammaTry thetaTry
amino acid storage Lsp1beta Lsp1gamma
sterol transport Npc2g Npc2e
carbohydrate digestion Hexo1 Amyrel
fatty acid bgm

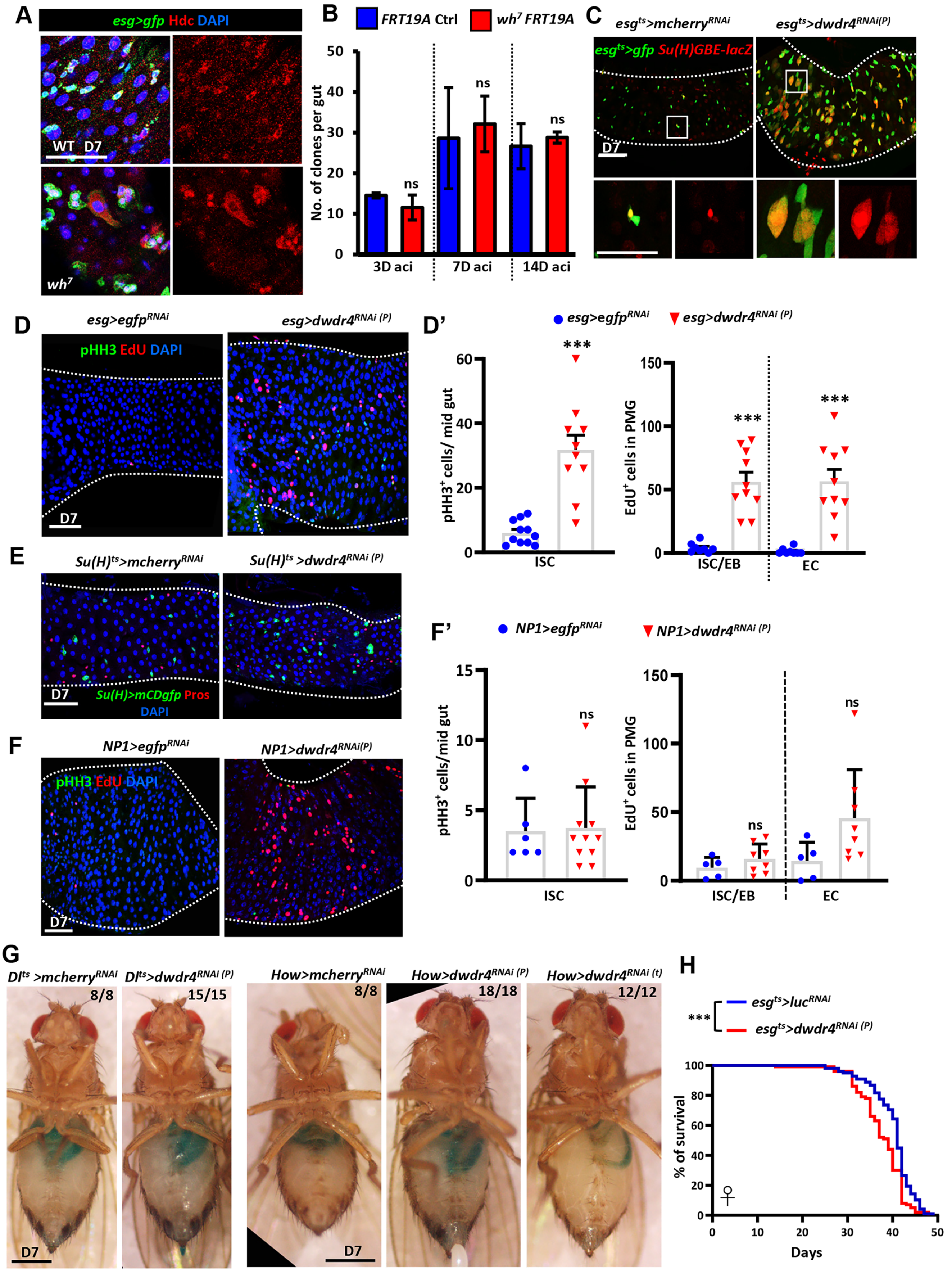
A
esg>gfp Hdc DAPI
WT D7
wh7
B
FRT19A Ctrl
wh7 FRT19A
No. of clones per gut
3D aci
7D aci
14D aci
ns
C
esgts>mcherryRNAi
esgts>dwdr4RNAi(P)
esgts>gfp Su(H)GBE-lacZ
D7
D
esg>egfpRNAi
esg>dwdr4RNAi (P)
pHH3 EdU DAPI
D7
D'
pHH3+ cells/ mid gut
EdU+ cells in PMG
ISC
ISC/EB
EC
***
E
Su(H)ts>mcherryRNAi
Su(H)ts >dwdr4RNAi (P)
Su(H)>mCDgfp Pros
DAPI
F
NP1>egfpRNAi
NP1>dwdr4RNAi(P)
F'
NP1>egfpRNAi
NP1>dwdr4RNAi (P)
pHH3+ cells/mid gut
G
Dlts >mcherryRNAi
Dlts>dwdr4RNAi (P)
How>mcherryRNAi
How>dwdr4RNAi (P)
How>dwdr4RNAi (t)
8/8
15/15
18/18
12/12
H
esgts>lucRNAi
esgts>dwdr4RNAi (P)
% of survival
Days

Figure EV2. Loss of dWdr4 in ISCs promotes their proliferation and shortens lifespan but does not compromise intestinal epithelial integrity.

(**A**) Posterior midguts (PMGs) from wild-type (WT) and *dwdr4* mutant (wh^7) females expressing *esg>gfp* (green, ISCs, EBs), stained for Hdc (red, ISCs/EBs) and DAPI (blue, nuclei). In the WT control PMG ($N = 10$), the ISC-EB two-cell cluster has both cells expressing Hdc, whereas in the *dwdr4* mutant (wh^7) PMG ($N = 13$), Hdc expression was seen in all *esg>gfp* cell clusters as well as in misdifferentiated ECs. (**B**) Number (no.) of clones per midgut in *FRT19A* control (ctrl) (3D: $N = 27$, 7D: $N = 30$, 14D: $N = 34$) and wh^7*FRT19A* heterozygous mutant guts (3D: $N = 29$, 7D: $N = 28$, 14D: $N = 28$) at 3, 7, and 14 days after clone induction (aci). (**C**) esg^{ts}>$dwdr4^{RNAi(P)}$ PMG ($N = 7$) shows expansion of ISCs/EBs (green, labeled with esg^{ts}>*gfp*) with some ISCs/EBs also expressing *Su(H)-lacZ* (red, a Notch signaling reporter used as an EB marker). In the esg^{ts}>$mcherry^{RNAi}$ control PMG ($N = 5$), the ISC-EB two-cell cluster has only one *Su(H)-lacZ*-positive cell. Enlarged views of the white boxed regions are shown below. (**D**, **D′**) *esg*>$dwdr4^{RNAi(p)}$ PMG ($N = 10$) shows increased mitotic ISCs (PHH3$^+$, green), proliferating ISCs/EBs (EdU$^+$ with the small nuclei, red), and endoreplicating ECs (EdU$^+$ with the large nucleus, red), as compared to the control (*esg*>$egfp^{RNAi}$) ($N = 8$). (**D′**) Number of PHH3$^+$ ISCs per midgut (left) and number of EdU$^+$ ISCs/EBs and ECs per PMG (right) for the indicated genotypes. (**E**) Knockdown of *dwdr4* in EBs ($Su(H)^{ts}$>$dwdr4^{RNAi(P)}$) ($N = 22$) causes no obvious change in the EB population ($Su(H)^{ts}$>*mcD8gfp*, green) compared to $Su(H)^{ts}$>$mcherry^{RNAi}$ control PMGs ($N = 20$). Pros, red, pEEs/EEs. (**F**, **F′**) Knockdown of *dwdr4* in ECs (*NP1*>$dwdr4^{RNAi(P)}$) ($N = 11$) does not increase mitotic ISCs (PHH3$^+$, green) or proliferating ISCs/EBs (EdU$^+$) compared to the control (*NP1*>$egfp^{RNAi}$) ($N = 6$). Still, it slightly increases endoreplicating ECs (EdU$^+$ with the large nucleus, red) compared to the control. DAPI, blue. (**F′**) Number of PHH3$^+$ ISCs per midgut (left) and number of EdU$^+$ ISCs/EBs and ECs per PMG (right) for the indicated genotypes. (**G**) Dl^{ts}>$dwdr4^{RNAi(P)}$, *how*>$dwdr4^{RNAi(P)}$, and *how*>$dwdr4^{RNAi(t)}$ female flies do not show the leaky gut phenotype, as revealed by the smurf assay. Fractions of flies with the pattern shown are indicated. (**H**) Female flies carrying esg^{ts}>$dwdr4^{RNAi(P)}$ have a shortened lifespan compared to control (esg^{ts}>*LucRNAi*) ($N = 100$ flies). Each dot in (**D′**, **F′**) represents one midgut. The scale bar in (**A**) and enlarged views of (**C**) are shown at 25 µm; (**C**, **D**, **E**, **F**) are 50 µm, and in (**G**) is 0.5 mm. White dashed lines in (**C**, **D**, **E**, **F**) outline the edge of the gut. Bars represent the mean; error bars indicate standard deviation (SD). Statistically significant differences in (**B**) were identified by the unpaired Student's *t* test, in (**D′**, **F′**) by the non-parametric Mann–Whitney *U* test, and in H by the Log-rank test. (**B**) No differences were observed between *FRT19A* ctrl and wh^7 *FRT19A* in clones per gut after 3D aci (ns, $P = 0.23$), 7D aci (ns, $P = 0.69$), and 14D aci (ns, $P = 0.57$). (**D′**) PHH3$^+$ cells were significantly increased in *esg*>$dwdr4^{RNAi(P)}$ compared with *esg*>$egfp^{RNAi}$ (***$P = < 0.0001$); EdU$^+$ ISC/EB (***$P = < 0.0001$) and EC cells (***$P = < 0.0001$) showed significant increase. (**F′**) no differences in PHH3$^+$ cells were observed in *NP1*>$dwdr4^{RNAi(P)}$ compared with *NP1*>$egfp^{RNAi}$ (ns, $P = 0.9$); EdU$^+$ ISC/EB (ns, $P = 0.3$) showed no difference; whereas in EC cells (ns, $P = 0.6$) increased slightly. Lifespan analysis in (**H**) showed a significant reduction in esg^{ts}>$dwdr4^{RNAi(P)}$ compared with *esg*>$egfp^{RNAi}$ (***$P = 0.0003$). *N*, number of midguts; unless otherwise stated. Source data are available online for this figure.

© The Author(s)

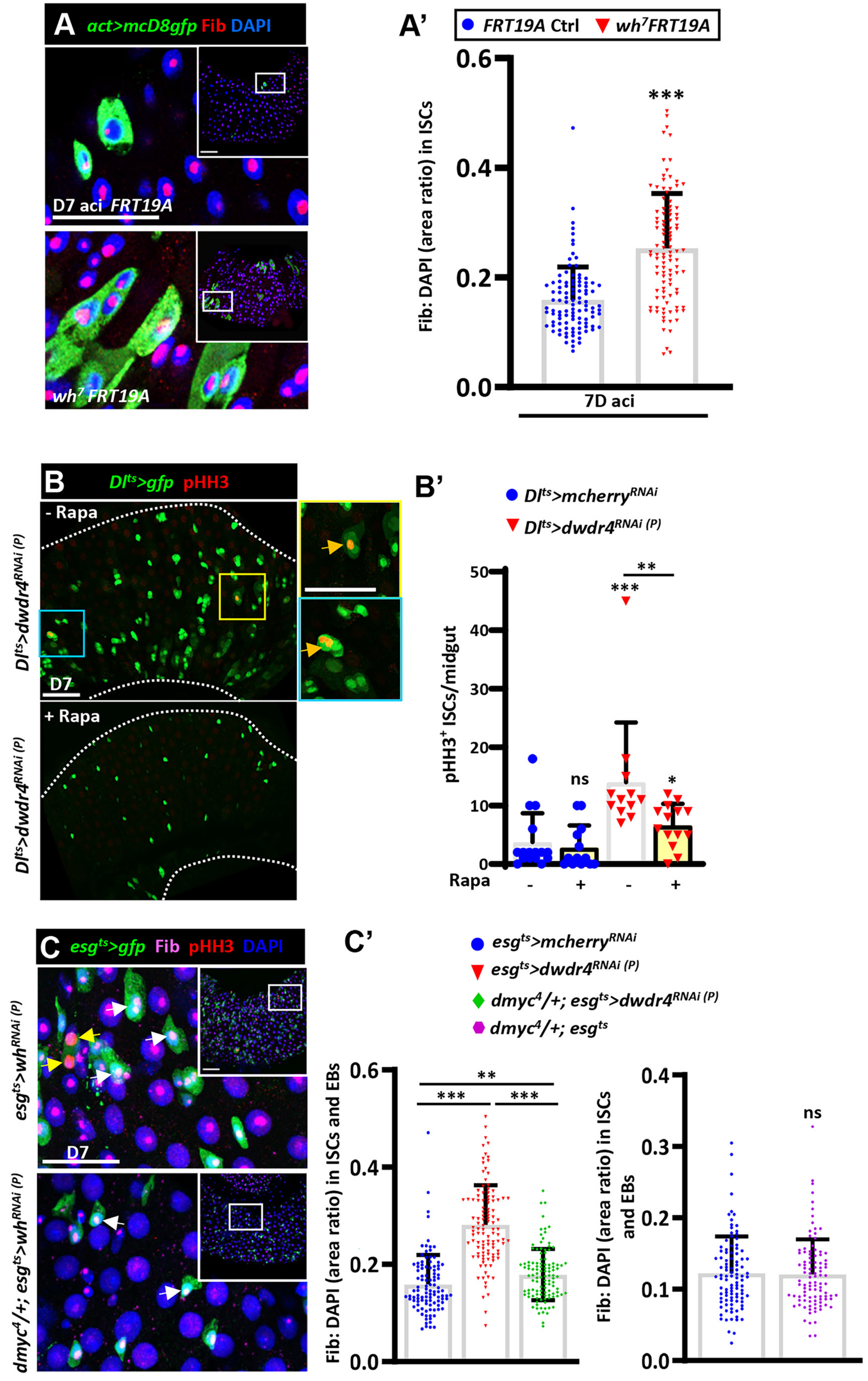
A act>mcD8gfp Fib DAPI
D7 aci FRT19A
wh7 FRT19A
A'
FRT19A Ctrl
wh7FRT19A
Fib: DAPI (area ratio) in ISCs
***
0.0
0.2
0.4
0.6
7D aci
B Dlts>gfp pHH3
- Rapa
+ Rapa
D7
Dlts>dwdr4RNAi (P)
B'
Dlts>mcherryRNAi
Dlts>dwdr4RNAi (P)
pHH3+ ISCs/midgut
**
***
ns
*
Rapa
C esgts>gfp Fib pHH3 DAPI
esgts>whRNAi (P)
dmyc4/+; esgts>whRNAi (P)
C'
esgts>mcherryRNAi
esgts>dwdr4RNAi (P)
dmyc4/+; esgts>dwdr4RNAi (P)
dmyc4/+; esgts
Fib: DAPI (area ratio) in ISCs and EBs
ns

Figure EV3. Wdr4 regulates ISC proliferation via TOR and dMyc.

(**A**, **A′**) dWdr4 cell-autonomously controls ribosome biogenesis in ISCs/EBs. (**A**) Mosaic *FRT19A* control ($N = 11$) and *wh⁷FRT19A* heterozygous mutant posterior midguts (PMGs) ($N = 10$) ($n = 103$ ISC/EB cells) with *act>mCD8gfp* (green, mutant cells), Fibrillarin (Fib) (red, nucleoli), and DAPI (blue, nuclei) labels. (**A′**) Quantification of nucleolar (Fib) to nuclear (DAPI) area ratio in GFP-positive cells with small nuclei in the indicated genotypes, at 7 days after clone induction (aci). (**B**, **B′**) Rapamycin treatment suppresses ISC expansion and overproliferation caused by dWdr4 depletion. (**B**) PMGs from Dl^{ts}*>dwdr4*$^{RNAi(P)}$ flies expressing Dl^{ts}*>gfp* (green, ISCs) with or without rapamycin (rapa) treatment, stained for PHH3 (red, mitosis marker). Enlarged views of the boxed regions are shown on the right. (**B′**) Quantification of PHH3$^+$ ISCs per midgut for the Dl^{ts}*>dwdr4*$^{RNAi(P)}$ (dH_2O: $N = 12$ midguts; Rapa: $N = 14$) and Dl^{ts}*>mcherry*^{RNAi} (dH_2O: $N = 15$; Rapa: $N = 13$). (**C**, **C′**) Removal of a copy of dMyc suppresses elevated ribosome biogenesis induced by dWdr4 depletion. (**C**) PMGs from esg^{ts}*>dwdr4*$^{RNAi(P)}$ and *dmyc*^{4}*/+; esg*^{ts}*>dwdr4*$^{RNAi(P)}$ females bearing esg^{ts}*>gfp* (green, ISCs/EBs), stained for PHH3 (red, mitosis marker) and Fibrillarin (Fib) (magenta, nucleoli) and DAPI (blue, nuclei). These images are also shown in Fig. 5F. (**C′**) Quantification of nucleolar (Fib) to nuclear (DAPI) area ratio in ISCs/EBs in the esg^{ts}*>mcherry*$^{RNAi(P)}$ ($N = 14$), esg^{ts}*>dwdr4*$^{RNAi(P)}$ ($N = 8$), esg^{ts}*>dwdr4*$^{RNAi(P)}$ with *dmyc*^{4} mutant ($N = 10$), and *dmyc*^{4} mutant alone ($N = 16$) (111 ISC/EB cells) flies. Yellow arrows in (**B**, **C**) indicate representative mitotic ISCs, and white arrows mark representative ISC or EB nucleoli. Insets in (**B**) show enlargements of boxed regions; (**A**, **C**) show enlargements of boxed regions within insets. Each dot in (**A′**, **C′**) represents a single ISC/EBs, and each dot in (**B′**) represents one midgut. The low magnification and enlarged views of (**A**, **C**) are shown at 50 and 25 µm; (**B**) is 50 µm. White dashed lines in (**B**) outline the edge of the gut. Bars represent the mean; error bars indicate standard deviation (SD). Statistically significant differences in (**A′**, **B′**, **C′**) were identified by the non-parametric Mann–Whitney *U* test. (**A′**) The nucleolar size differed in *FRT19A* and *wh⁷FRT19A* (***$P = < 0.0001$). (**B′**) PHH3$^+$ cells were unchanged by rapamycin in Dl^{ts}*>mcherry*^{RNAi} (ns, $P = 0.32$), but differed between Dl^{ts}*>mcherry*^{RNAi} and Dl^{ts}*>dwdr4*$^{RNAi(P)}$ with (*$P = 0.03$) and without rapamycin (***$P = < 0.0001$); in Dl^{ts}*>dwdr4*$^{RNAi(P)}$, rapamycin significantly reduced PHH3$^+$ cells (**$P = 0.002$). (**C′**, left graph) The nucleolar size showed differences in esg^{ts}*>dwdr4*$^{RNAi(P)}$ compared to esg^{ts}*>mcherry*$^{RNAi(P)}$ without *dmyc*^{4} (***$P = < 0.0001$) and with *dmyc*^{4} (**$P = 0.0012$); nucleolar size differed significantly between esg^{ts}*>dwdr4*$^{RNAi(P)}$ with and without *dmyc*^{4} (***$P = < 0.0001$); whereas *dmyc*^{4} alone had no effects (ns, $P = 0.75$) (right graph). *N*, number of midguts; *n*, number of ISCs or ISC/EBs analyzed, unless otherwise stated. Source data are available online for this figure.

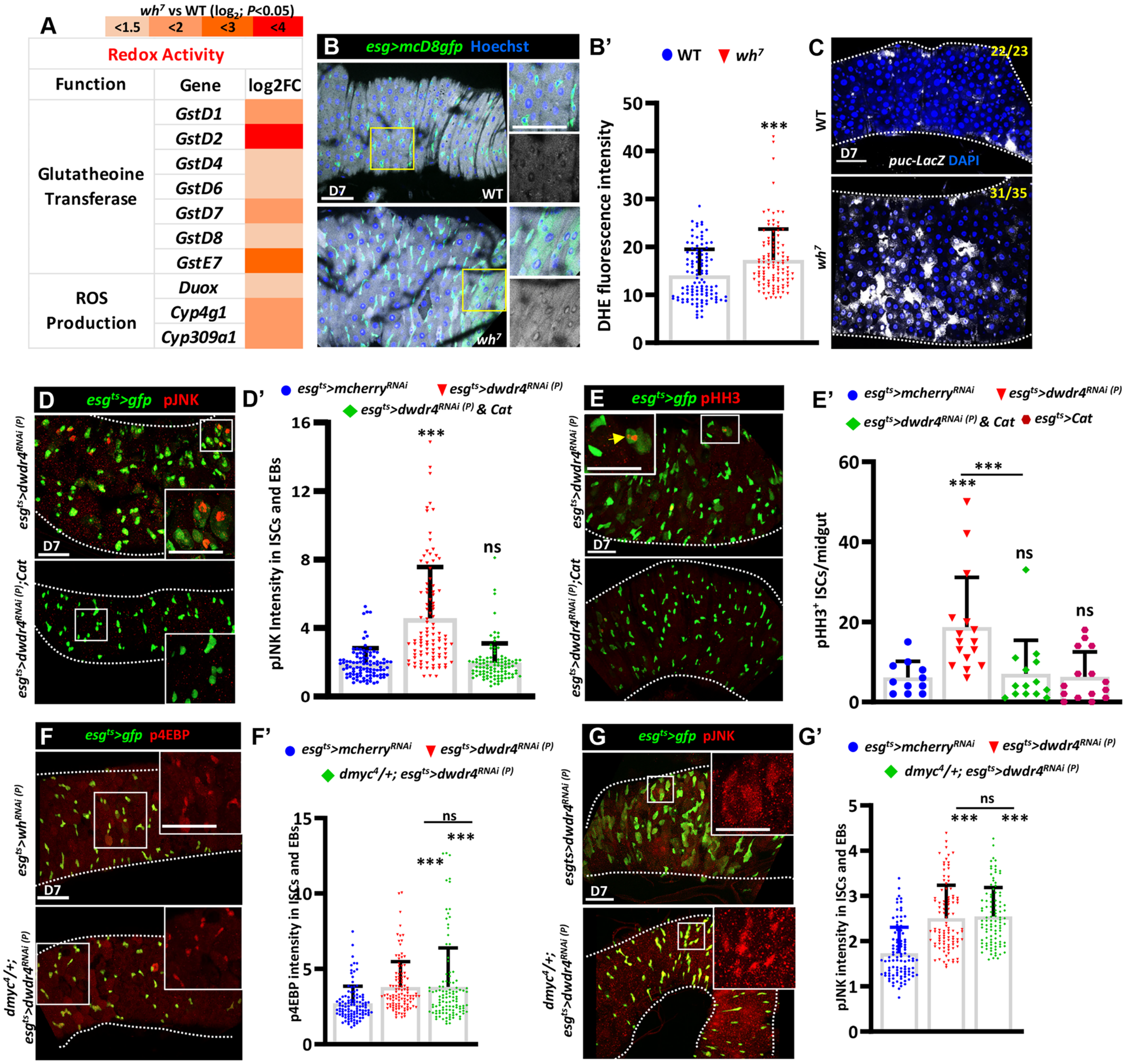
A
wh7 vs WT (log2; P<0.05)
<1.5 <2 <3 <4
Redox Activity
Function Gene log2FC
Glutatheoine Transferase
GstD1
GstD2
GstD4
GstD6
GstD7
GstD8
GstE7
ROS Production
Duox
Cyp4g1
Cyp309a1
B esg>mcD8gfp Hoechst
D7
WT
wh7
B'
WT wh7
DHE fluorescence intensity
***
C
22/23
31/35
WT
wh7
D7
puc-LacZ DAPI
D esgts>gfp pJNK
esgts>dwdr4RNAi (P)
esgts>dwdr4RNAi (P);Cat
D'
esgts>mcherryRNAi
esgts>dwdr4RNAi (P)
esgts>dwdr4RNAi (P) & Cat
pJNK intensity in ISCs and EBs
ns
E esgts>gfp pHH3
E'
esgts>Cat
pHH3+ ISCs/midgut
F esgts>gfp p4EBP
esgts>whRNAi (P)
dmyc4/+; esgts>dwdr4RNAi (P)
F'
p4EBP intensity in ISCs and EBs
G esgts>gfp pJNK
esgts>dwdr4RNAi (P)
G'

Figure EV4. Loss of Wdr4 in the gut increases expression of genes involved in stress and redox responses as well as JNK signaling.

(**A**) RNA-seq analysis results show that the expression levels of genes involved in redox activity and stress are increased in the guts of *dwdr4* (*wh*^{7}) mutant female guts. Fold change of log_2 values ($P < 0.05$) is indicated by color. Statistical analysis was performed on two biological replicates. (**B**, **B′**) *wh*^{7} mutant PMG bearing *esg>mCD8gfp* ($N = 15$) (green, ISCs/EBs) show increased ROS levels compared to WT controls ($N = 16$), ($n = 110$ ISC/EB cells), as detected by DHE staining (gray). (**B′**) Quantification of DHE fluorescence intensity. DAPI (bule) label nuclei. (**C**) *wh*^{7} mutant PMG shows increased JNK signaling compared to controls ($N = 7$), as revealed by *puc-lacZ* (gray, a JNK signaling reporter). DAPI, blue. Fractions of gut sections with the shown pattern are indicated. (**D**, **E′**) Overexpression of Catalases (Cat) suppresses elevated pJNK and ISC expansion and overproliferation induced by dWdr4 depletion. (**D**) PMGs from the indicated genotypes bearing *esg*^{ts}*>gfp* (green, ISCs/EBs), stained for pJNK (Red, JNK signaling activity). (**D′**) Quantification of pJNK intensity in the *esg*^{ts}*>mcherry*$^{RNAi(P)}$ ($N = 11$), *esg*^{ts}*>dwdr4*$^{RNAi(P)}$ ($N = 16$), and *esg*^{ts}*>dwdr4*$^{RNAi(P)}$ with *cat* overexpression ($N = 16$) ($n = 100$ ISC/EB cells) flies. (**E**) PMGs from the indicated genotypes bearing *esg*^{ts}*>gfp* (green), stained for PHH3 (red, mitotic marker). (**E′**) Quantification of PHH3^{+} ISCs per midgut for the *esg*^{ts}*>mcherry*$^{RNAi(P)}$ ($N = 11$), *esg*^{ts}*>dwdr4*$^{RNAi(P)}$ ($N = 16$), *esg*^{ts}*>dwdr4*$^{RNAi(P)}$ with *cat*^{OE} ($N = 14$), and *cat*^{OE} alone ($N = 15$) flies. (**F**, **G**) dMyc acts downstream of TOR and JNK signaling in ISCs for ISC homeostasis. (**F**, **F′**) Loss of one *dmyc* copy in *esg*^{ts}*>dwdr4*$^{RNAi(P)}$ posterior midguts (PMGs) fails to suppress ISC/EB expansion (*esg*^{ts}*>gfp*, green) or the increased TOR signaling (Red, TOR signaling activity). (**F′**) Quantification of average p4EBP intensity in the *esg*^{ts}*>mcherry*$^{RNAi(P)}$ ($N = 11$), *esg*^{ts}*>dwdr4*$^{RNAi(P)}$ ($N = 12$), *esg*^{ts}*>dwdr4*$^{RNAi(P)}$ with *dmyc*^{4} mutant ($N = 10$) ($n = 110$ ISC/EB cells) flies. (**G**, **G′**) Removal of one copy of *dmyc* in *esg*^{ts}*>dwdr4*$^{RNAi(P)}$ PMGs does not suppress the increased JNK signaling (Red, JNK signaling activity). (**G′**) Quantification of average pJNK intensity in the *esg*^{ts}*>mcherry*$^{RNAi(P)}$ ($N = 9$), *esg*^{ts}*>dwdr4*$^{RNAi(P)}$ ($N = 16$), *esg*^{ts}*>dwdr4*$^{RNAi(P)}$ with *dmyc*^{4} mutant ($N = 8$) ($n = 110$ ISC/EB cells) flies. Insets in (**B**, **D**, **E**, **F**, **G**) show enlarged views of the boxed regions. Each dot in (**B′**, **D′**, **F′**, **G′**) represents a single ISC or EB, and each dot in (**E′**) represents one midgut. The image and insets of (**B**, **D–G**) are shown at 50 and 25 µm. White dashed lines in (**C–G**) outline the edge of the gut. Bars represent the mean; error bars indicate standard deviation (SD). Statistically significant differences in (**B′–G′**) were identified by the non-parametric Mann–Whitney *U* test. (**B′**) The DHE signals increased in *wh*^{7} compared to WT (***$P = < 0.0001$). (**D′**) The pJNK intensity showed differences in *esg*^{ts}*>dwdr4*$^{RNAi(P)}$ compared to *esg*^{ts}*>mcherry*$^{RNAi(P)}$ without *cat*^{OE} (***$P = < 0.0001$), but not with *cat*^{OE} (ns, $P = 0.7$). (**E′**) The PHH3^{+} cells showed differences in *esg*^{ts}*>dwdr4*$^{RNAi(P)}$ compared to *esg*^{ts}*>mcherry*$^{RNAi(P)}$ without *cat*^{OE} (***$P = 0.0002$), but not with *cat*^{OE} (ns, $P = 0.67$); PHH3^{+} cells differed between *esg*^{ts}*>dwdr4*$^{RNAi(P)}$ with and without *cat*^{OE} (***$P = 0.0003$); whereas *cat*^{OE} alone had no effects (ns, $P = 0.58$). (**F′**, **G′**) p4EBP and pJNK levels showed differences in *esg*^{ts}*>dwdr4*$^{RNAi(P)}$ compared to *esg*^{ts}*>mcherry*$^{RNAi(P)}$ without *dmyc*^{4} and with *dmyc*^{4} (***$P = < 0.0001$); p4EBP and pJNK levels did not differ between *esg*^{ts}*>dwdr4*$^{RNAi(P)}$ with and without *dmyc*^{4} (ns, $P = 0.52$ in **F′**, 0.42 in **G′**). *N*, number of midguts; *n*, number of ISCs or ISC/EBs analyzed, unless otherwise stated. Source data are available online for this figure.

© The Author(s)

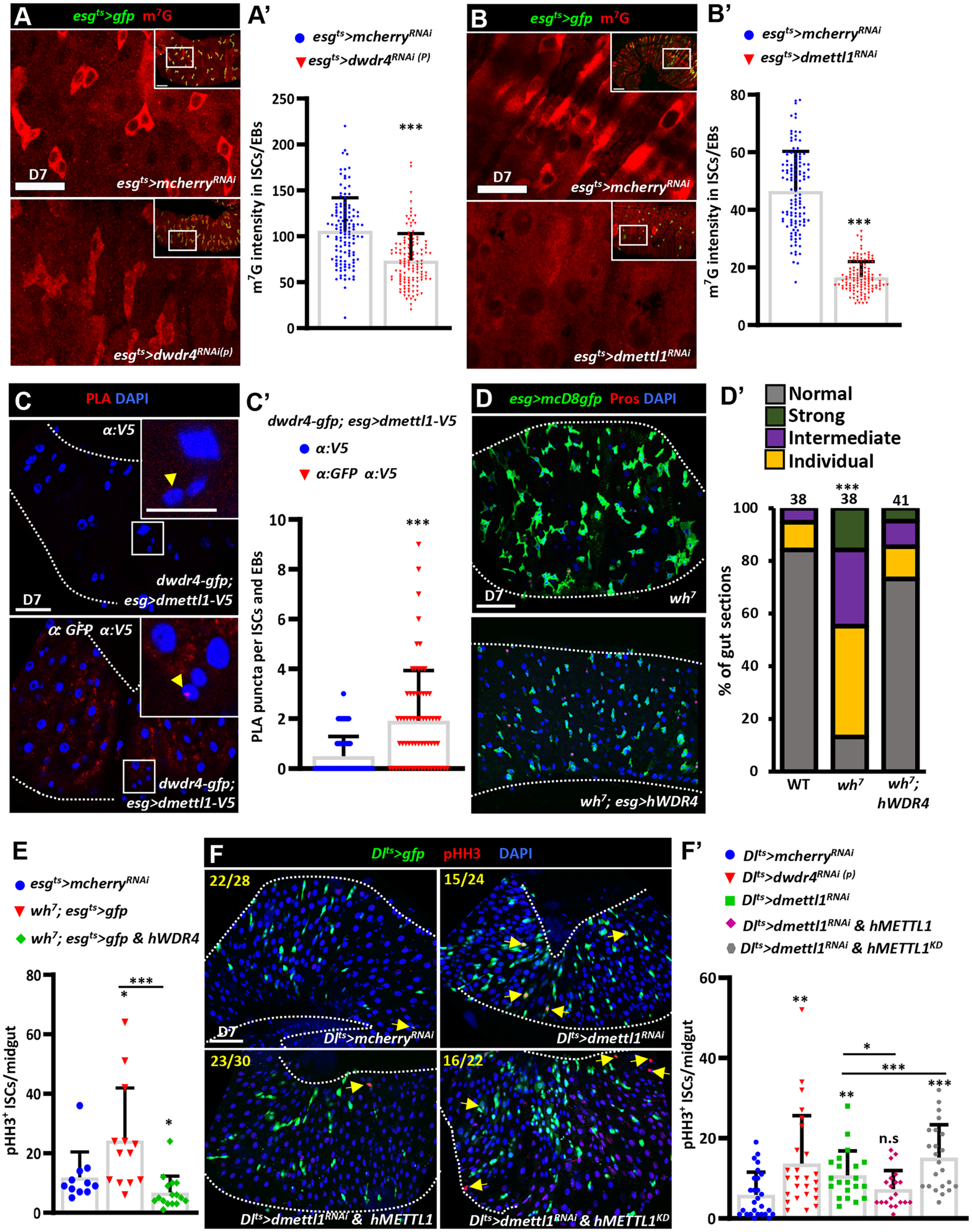
A
esgts>gfp m7G
D7
esgts>mcherryRNAi
esgts>dwdr4RNAi(p)
A'
esgts>mcherryRNAi
esgts>dwdr4RNAi (P)
m7G intensity in ISCs/EBs
***
B
esgts>gfp m7G
D7
esgts>mcherryRNAi
esgts>dmettl1RNAi
B'
esgts>mcherryRNAi
esgts>dmettl1RNAi
m7G intensity in ISCs/EBs
***
C
PLA DAPI
α:V5
D7
dwdr4-gfp; esg>dmettl1-V5
α: GFP α:V5
dwdr4-gfp; esg>dmettl1-V5
C'
dwdr4-gfp; esg>dmettl1-V5
α:V5
α:GFP α:V5
PLA puncta per ISCs and EBs
***
D
esg>mcD8gfp Pros DAPI
D7
wh7
wh7; esg>hWDR4
D'
Normal
Strong
Intermediate
Individual
% of gut sections
38
***
38
41
WT
wh7
wh7; hWDR4
E
esgts>mcherryRNAi
wh7; esgts>gfp
wh7; esgts>gfp & hWDR4
pHH3+ ISCs/midgut
***
*
*
F
Dlts>gfp pHH3 DAPI
22/28
15/24
23/30
16/22
D7
Dlts>mcherryRNAi
Dlts>dmettl1RNAi
Dlts>dmettl1RNAi & hMETTL1
Dlts>dmettl1RNAi & hMETTL1KD
F'
Dlts>mcherryRNAi
Dlts>dwdr4RNAi (p)
Dlts>dmettl1RNAi
Dlts>dmettl1RNAi & hMETTL1
Dlts>dmettl1RNAi & hMETTL1KD
pHH3+ ISCs/midgut
**
*
***
**
n.s
***

© The Author(s)

Figure EV5. The *Drosophila* Wdr4-Mettl1 complex mediates m^{7}G modification to maintain intestinal homeostasis in a conserved manner.

(**A**, **B′**) dWdr4 and dMettl1 control m^{7}G levels in ISCs/EBs. (**A**, **B**) Posterior midguts (PMGs) from *esg^{ts}>dwdr4$^{RNAi(P)}$* (**A**) and *esg^{ts}>dmettl1^{RNAi}* ($N = 16$) (**B**) females show decreased m^{7}G modification (red) in ISCs/EBs (*esg^{ts}>gfp*, green) as compared to control (*esg^{ts}>mcherry^{RNAi}*) ($N = 12$) ($n = 125$ ISC/EB cells). (**A′**, **B′**) Quantification of average m^{7}G fluorescence intensity in ISCs/EBs in the indicated genotypes. (**C**) dWdr4 interacts with dMettl1 in ISCs/EBs, as revealed by PLA assay (red punctate) using anti-GFP and anti-V5 antibodies in PMGs from *dwdr4-gfp; esg>dmettl1-V5* females. *dmettl1*-V5 is expressed in ISCs/EBs under the control of *esg-GAL4*, while *dwdr4-gfp* is expressed in all intestinal cells. PMGs of the same genotype exposed only to anti-V5 antibody served as a negative control. The arrowhead in C indicates the PLA puncta. DAPI (blue) labels nuclei. (**C′**) Quantification of PLA puncta number in the *dwdr4-gfp; esg>dmettl1-V5* females with and without anti-GFP antibody ($N = 8$, $n = 65$ ISC/EB cells). (**D**) Overexpression of human WDR4 in ISCs/EBs of *dwdr4* mutant (*wh^7*) ISCs/EBs (labeled by *esg>mCD8gfp*, green) suppresses ISC/EB expansion. Pros, red. DAPI, blue. The *wh^7* mutant image was obtained together with Fig. 2A and is shown here for comparison with the human gene rescue. (**D′**) Quantification of ISC/EB expansion phenotypes categorized and color-coded as Fig. 2A. The number of gut sections analyzed ($N = 9$) per genotype is shown above each bar. (**E**) Overexpression of human WDR4 in *dwdr4* mutant ISCs/EBs (*esg^{ts}>gfp*) decreases mitotic ISCs (PHH3$^+$) in midguts. Number of PHH3$^+$ cells per midgut in the *esg^{ts}>mcherry$^{RNAi(P)}$* ($N = 11$), *wh^7* mutant with *esg>gfp* ($N = 13$), *wh^7* mutant with *esg>gfp* and human WDR4 overexpression ($N = 16$) flies. (**F**) Overexpression of human METTL1, but not the catalytic-dead mutant (hMETTL1^{KD}) in ISCs (labeled by *Dl^{ts}>gfp*, green) of *dmettl1^{RNAi}* knockdown PMGs suppresses the increased ISC expansion and proliferation. Yellow arrows in E indicate the representative mitotic ISCs. Fractions of gut sections with the ISC/EB pattern shown are indicated. (**F′**) Quantification of PHH3$^+$ ISC number per midgut in the *Dl^{ts}>mcherry$^{RNAi(P)}$* ($N = 27$), *Dl^{ts}>dwdr4$^{RNAi(P)}$* ($N = 26$), *Dl^{ts}>dmettl1^{RNAi}* ($N = 21$), with human METTL1 ($N = 21$), and hMETTL1^{KD} ($N = 23$) flies. DAPI, blue. Insets in (**C**) show enlarged views of the white square in each panel; (**A**, **B**) show enlarged views of the square regions in the insets. Each dot in (**A′**, **B′**, **C′**) represents a single ISC or EB, and each dot in (**E′**, **F′**) represents one midgut. The low magnification and enlarged views of (**A**, **B**, **C**) are shown at 50 and 25 μm; (**D**, **F**) are 50 μm. White dashed lines in (**C**, **D**, **F**) outline the edge of the gut. Bars represent the mean; error bars indicate standard deviation (SD). Statistically significant differences in (**A′**, **B′**) were identified by unpaired Student's *t* test, in (**C′**, **E**, **F′**) were identified by non-parametric Mann–Whitney *U* test, and in (**D′**) were analyzed by Chi-square. (**A′**, **B′**) m7G intensity decreased in *esg^{ts}>dwdr4$^{RNAi(P)}$* and *esg^{ts}>dmettl1^{RNAi}* compared to *esg^{ts}>mcherry^{RNAi}* (***$P = < 0.0001$). (**C′**) The ***$P = < 0.0001$. (**D′**) The ***$P = 0.001$. (**E**) The PHH3$^+$ cells showed differences in *wh^7* compared to WT without *hWDR4* (*$P = 0.017$) and with *hWDR4* (*$P = 0.013$); PHH3$^+$ cells differed between *esg^{ts}>dwdr4$^{RNAi(P)}$* with and without *hWDR4* (***$P = < 0.0001$). (**F′**) The PHH3$^+$ cells showed differences in *Dl^{ts}>dwdr4$^{RNAi(P)}$* (**$P = 0.002$), *Dl^{ts}>dmettl1^{RNAi}* (**$P = 0.004$), *Dl^{ts}>dmettl1^{RNAi}* with *hMETTL1^{CD}* (***$P = < 0.0001$) compared to *Dl^{ts}>mcherry$^{RNAi(P)}$*; but no difference in *Dl^{ts}>dmettl1^{RNAi}* with *hMETTL1* (ns, $P = 0.16$). PHH3$^+$ cells differed in *Dl^{ts}>dmettl1^{RNAi}* with and without *hMETTL1* (*$P = 0.04$), but not with *hMETTL1^{CD}* (*$P = 0.0006$). *N*, number of midguts; *n*, number of ISCs or ISC/EBs analyzed, unless otherwise stated. Source data are available online for this figure.

© The Author(s)

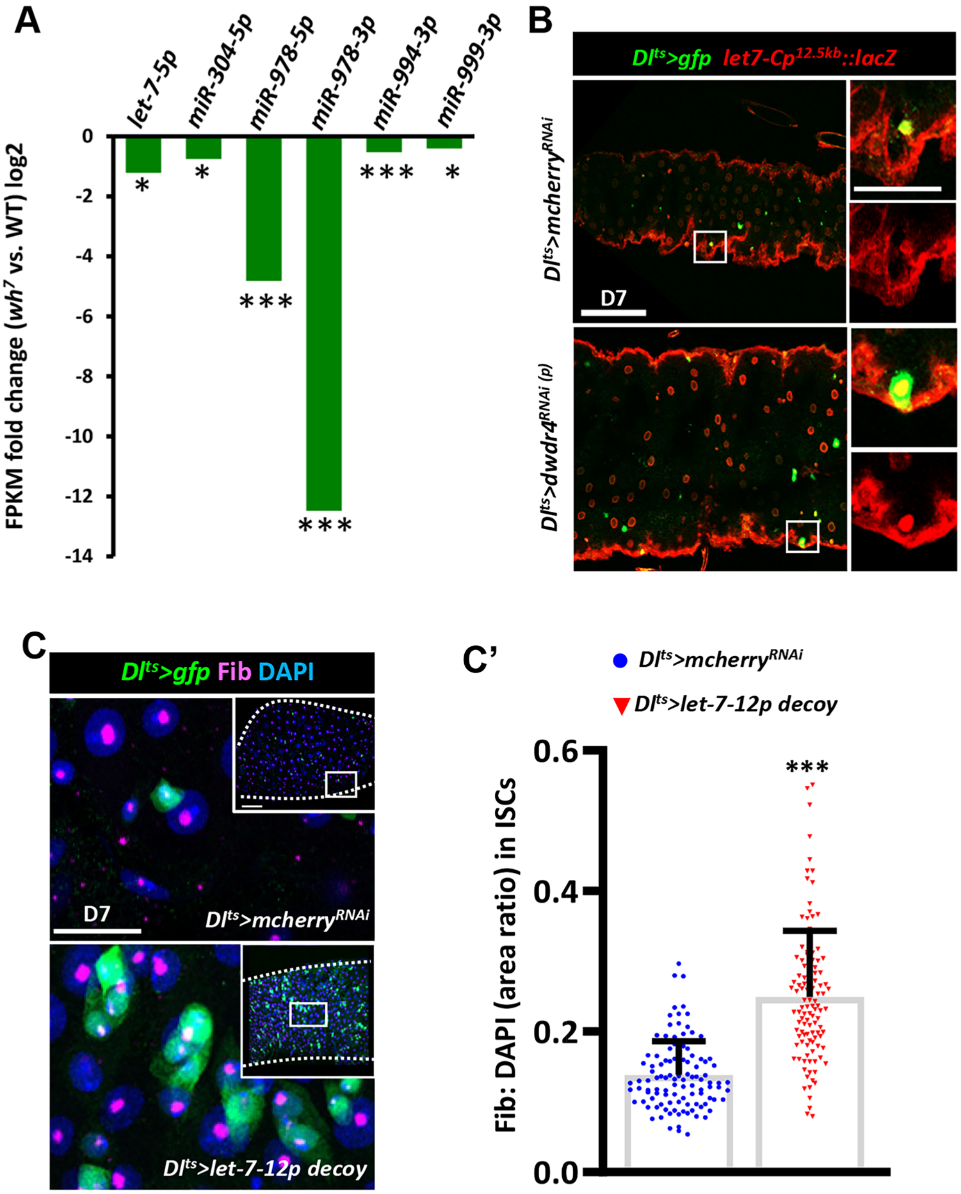

Figure EV6. dWdr4 does not control *let-7* transcription and *let-7* disruption phenocopies *dwdr4* mutant PMGs.

(**A**) Fold changes in gene expression according to RNA-seq (fragments per kilobase of transcript per million mapped reads [FPKM]) in wdr4 mutant (*wh*^{7}) vs. wild-type (WT) ovaries; $\log_2$ values are shown. Statistical analysis was performed on two biological replicates. *$P < 0.05$; ***$P < 0.001$. (**B**) Posterior midgut (PMG) from *Dl*^{ts}*>dwdr4*$^{RNAi(P)}$ ($N = 7$), females bearing *Dl*^{ts}*>gfp* (ISCs, green) show no obvious change in the expression of *let7-Cp*$^{12.5Kb}$*::lacZ* (red, transcriptional reporter of *let-7*) as compared to control (*Dl*^{ts}*>mcherry*^{RNAi}) ($N = 6$). (**C**) Disruption of *let-7* function (*Dl*^{ts}*>let-7-12p decoy*) ($N = 11$) increases the size of Fibrillarin (Fib) (magenta, nucleoli) in ISCs (labeled by *Dl*^{ts}*>gfp*, green) as compared to control (*Dl*^{ts}*>mcherry*^{RNAi}) ($N = 9$) ($n = 110$ ISC cells). DAPI (blue) marks nuclei. (**C′**) Quantification of nucleolar (Fib) to nuclear (DAPI) area ratio in ISCs in the indicated genotypes. Insets in (**B**) show enlarged views of the boxed region in each panel; (**C**) shows enlarged views of the boxed region in the insets. Each dot in (**C′**) represents a single ISC. The low magnification and enlarged views of (**B, C**) are shown at 50 and 25 µm. White dashed lines in (**C**) outline the edge of the gut. Bars represent the mean; error bars indicate standard deviation (SD). Statistically significant differences in (**A**) were identified by unpaired Student's *t* test, and in (**C′**) were by non-parametric Mann–Whitney *U* test. (**C′**) the nucleolar size is increased in *Dl*^{ts}*>let-7-12p decoy* compared to *Dl*^{ts}*>mcherry*^{RNAi} (***$P = < 0.0001$). *N*, number of midguts; *n*, number of ISCs or ISC/EBs analyzed, unless otherwise stated. Source data are available online for this figure.

 © The Author(s)
